# Augmenting hematoma-scavenging capacity of innate immune cells by CDNF reduces brain injury and promotes functional recovery after intracerebral hemorrhage

**DOI:** 10.1038/s41419-022-05520-2

**Published:** 2023-02-15

**Authors:** Kuan-Yin Tseng, Vassilis Stratoulias, Wei-Fen Hu, Jui-Sheng Wu, Vicki Wang, Yuan-Hao Chen, Anna Seelbach, Henri J. Huttunen, Natalia Kulesskaya, Cheng-Yoong Pang, Jian-Liang Chou, Maria Lindahl, Mart Saarma, Li-Chuan Huang, Mikko Airavaara, Hock-Kean Liew

**Affiliations:** 1 Department of Neurological Surgery, Tri-Service General Hospital and National Defense Medical Center, No.325, 2nd Sec., Cheng-Kung Road, Nei-Hu District, Taipei City, 114 Taiwan Taiwan, ROC; 2 Neuroscience Center, HiLIFE, Haartmaninkatu 8, FI-00014, University of Helsinki, Helsinki, Finland; 3 PhD Program in Pharmacology and Toxicology, Tzu Chi University, No.701, Sec.3, Zhong-Yang Road, 970 Hualien County, Hualien, Taiwan; 4 Herantis Pharma Ltd, Plc, Bertel Jungin aukio 1, FI-02600 Espoo, Finland; 5 Institute of Medical Sciences, Tzu Chi University, No.701, Sec.3, Zhong-Yang Road, 970 Hualien County, Hualien, Taiwan; 6 Neuro-Medical Scientific Center, Hualien Tzu Chi Hospital, Buddhist Tzu Chi Medical Foundation, No.707, Sec.3, Zhong-Yang Road, 970 Hualien County, Hualien, Taiwan; 7 Department of Medical Research, Hualien Tzu Chi Hospital, Buddhist Tzu Chi Medical Foundation, No.707, Sec.3, Zhong-Yang Road, 970 Hualien County, Hualien, Taiwan; 8 Graduate Institute of Medical Sciences, National Defense Medical Center, Taipei, Taiwan; 9 Institute of Biotechnology, HiLIFE, Viikinkaari 5D, FI-00014, University of Helsinki, Helsinki, Finland; 10 Department of Medical Imaging, Hualien Tzu Chi Hospital, Buddhist Tzu Chi Medical Foundation, No.707, Sec.3, Zhong-Yang Road, 970 Hualien County, Hualien, Taiwan; 11 Faculty of Pharmacy, Viikinkaari 5E, FI-00014, University of Helsinki, Helsinki, Finland

**Keywords:** Neuroimmunology, Stroke, Neurotrophic factors

## Abstract

During intracerebral hemorrhage (ICH), hematoma formation at the site of blood vessel damage results in local mechanical injury. Subsequently, erythrocytes lyse to release hemoglobin and heme, which act as neurotoxins and induce inflammation and secondary brain injury, resulting in severe neurological deficits. Accelerating hematoma resorption and mitigating hematoma-induced brain edema by modulating immune cells has potential as a novel therapeutic strategy for functional recovery after ICH. Here, we show that intracerebroventricular administration of recombinant human cerebral dopamine neurotrophic factor (rhCDNF) accelerates hemorrhagic lesion resolution, reduces peri-focal edema, and improves neurological outcomes in an animal model of collagenase-induced ICH. We demonstrate that CDNF acts on microglia/macrophages in the hemorrhagic striatum by promoting scavenger receptor expression, enhancing erythrophagocytosis and increasing anti-inflammatory mediators while suppressing the production of pro-inflammatory cytokines. Administration of rhCDNF results in upregulation of the Nrf2-HO-1 pathway, but alleviation of oxidative stress and unfolded protein responses in the perihematomal area. Finally, we demonstrate that intravenous delivery of rhCDNF has beneficial effects in an animal model of ICH and that systemic application promotes scavenging by the brain’s myeloid cells for the treatment of ICH.

## Introduction

Intracerebral hemorrhage (ICH) is a devastating neurological disease with a high mortality rate and severe neurological deficits in those who survive. It is caused by spontaneous rupture of intracranial vessels or hemorrhagic transformation of cerebral infarction. The latter frequently occurs as a major complication of thrombolytic therapy for acute ischemic stroke [1]. Rapid accumulation of blood within the brain parenchyma leads to mechanical pressure and perihematomal blood flow reduction that results in primary brain damage. However, only a quarter of ICH-related neurological deterioration occurs within the first 24 h, emphasizing the relevance of secondary brain injury in the development of ICH. During secondary brain injury, blood cells lyse, generating neurotoxic products (e.g. hemoglobin/heme/iron) and reactive oxygen species (ROS) [2]. ROS accumulation interrupts reduction-oxidation (redox) balancing leading to oxidative stress and induces the PERK/eIF2α/ATF4 stress response, which contribute to global protein synthesis inhibition and adaptive ATF4-mediated gene expression [3]. However, overwhelming production of ROS may disrupt redox homeostasis of the endoplasmic reticulum (ER) and trigger apoptotic signaling upon ER response dysfunction. Impaired redox capacity and prolonged ER stress activate inflammatory signaling cascades through the interaction between unfolded protein response (UPR) components and canonical cytokine-regulated transcription factors [4]. Subsequently, resting microglia are activated to release more cytokines and chemokines, which recruit potentially damaging monocytes/macrophages from the periphery into the ICH-affected brain [5]. Taken together, a complex sequence of parallel and sequential deleterious mechanisms, including inflammation, oxidative stress, and terminal UPR pathways, cause more neuronal cell death, accelerate the formation of edema around hematomas, amplify blood-brain barrier (BBB) disruption and finally result in long-term functional impairment. Current therapeutic strategies for ICH address reducing hematoma expansion and preventing medical complications [6]. However, there are no medical therapies or surgical interventions that significantly decrease peri-hematoma edema and improve human functional outcomes after ICH.

Scavenger cells, including resident microglia and infiltrating macrophages, respond quickly to brain injuries such as ICH, and engulf foreign substances and cell debris [7]. While microglia/macrophages play an essential role in removing the hematoma and clearing debris, they are a source of ongoing inflammation [8]. Activated microglia/macrophages could exacerbate brain damage by eliciting inflammatory properties’ expression [5]. However, to date, anti-inflammatory agents have failed to show beneficial effects in hemorrhagic stroke clinical trials. These disappointing outcomes may be due to suppression of microglia/macrophages, thereby also removing the normal defensive functions of the CNS [9]. It is worth noting that well-timed inflammatory reactions serve many neuroprotective functions, whereas overwhelming or chronic inflammation is more likely to result in deterioration of functional outcomes [10]. Therefore, experimental stroke therapies should be modified from blanketed microglia/macrophage suppression towards a more fine-tuned adaption of the balance between protective and cytotoxic microglia/macrophage phenotypes. Phagocyte activation inevitably releases pro-inflammatory cytokines and cytotoxic materials during hematoma resolution, detrimental to neighboring cells, and leading to secondary brain injury. Promoting phagocytosis dynamically and appropriately may limit the toxic effects of persistent blood-lysed products on surrounding tissue [11]. This paradigm may be necessary for recovery after ICH. Recent studies demonstrate that enhancing hematoma-scavenging functions speeds up hematoma clearance, mitigates oxidative stress, harmonizes inflammatory responses, and promotes functional recovery after ICH [12]. Therefore, scavenger receptors, expressed on microglia/macrophages, astrocytes, or endothelial cells, play essential roles in the process of hematoma resolution and immune modulation.

Cerebral dopamine neurotrophic factor (CDNF) and mesencephalic astrocyte-derived neurotrophic factor (MANF) are evolutionarily-conserved proteins, forming a family with pleiotropic effects [13]. Increasing evidence indicates that CDNF and MANF, when applied as extracellular proteins or delivered by viral vectors, can protect and repair various cell types in vivo [14]. Although CDNF and MANF can be secreted by cells, they are primarily retained intracellularly in the ER lumen [15]. In line with their ER localization, CDNF and MANF are upregulated on UPR and counteract ER stress in different disease models [16]. In vitro data indicate that CDNF expression is induced by ER stress in cultured neurons, and that CDNF expression improves neuronal viability by upregulating several proteins involved in UPR signaling, including GRP78, ATF4, ATF6, and XBP1, while reducing activation of ER stress-responsive apoptotic proteins, such as CHOP [17]. In parallel to direct cytoprotection, CDNF and MANF potentiate immune modulation in vivo, acting on stem cells and promoting regeneration [18, 19]. Furthermore, increasing number of studies indicate that CDNF can directly attenuate the production of proinflammatory cytokines in vitro by regulating activation of myeloid cells under pathological stress [20, 21]. However, they do not elucidate whether this effect is a consequence of protecting microglia against injuries or a mechanism involving dynamic balance between pro-inflammatory and anti-inflammatory microglial states. In addition, it remains to be determined whether inhibition of immune activation by CDNF is associated with modulation of microglia/macrophage phenotypes in vivo.

In this study, we first characterized the time course of endogenous CDNF expression within the peri-hematoma area in a rat model of hemorrhagic stroke. Further, by assessing the lesioned volume and transcriptomic profile from control and CDNF knockout mice exposed to a hemorrhagic insult, we demonstrate that endogenous CDNF reduced hematoma accumulation possibly through early induction of a heme-catalyzing enzyme, HO-1. Importantly, we show that post-ICH delivery of CDNF accelerates hematoma resolution, mitigates secondary brain injury, and improves functional recovery. CDNF also upregulates the Nrf2-Heme Oxygenase-1 axis and dynamically enhances CD36 and CD163 expression, potential endogenous scavenger receptors on microglia/macrophages, to enhance erythrophagocytosis in the hemorrhagic striatum. Simultaneously, amelioration of oxidative stress and UPR by CDNF remodels the peri-hematoma milieu from a pro-inflammatory microenvironment to a pro-reparative one. Finally, intravenous therapy with rhCDNF post-ICH reduces striatal injury and neurological deficits, probably via modulating brain myeloid cell’s scavenging function. This available route of administration makes CDNF a potential candidate for development into a clinically therapeutic regimen.

## Materials and methods

### Study design

In all experiments, animals were randomly assigned to treatment groups using a random number code before their initial acclimation on behavior tasks. The investigator carried out surgery and behavior tests in a blinded manner. Animals were excluded only if mortality occurred during surgery, before surgery, or before treatment administration (<5% of animals). Endpoints were determined by time post-ICH induction and were 24 h, 72 h, or 7 days, or for humane reasons defined by institutional guidelines.

Our initial studies were characterizing CDNF protein and expression in the peri-hematoma tissue over 7 days post-ICH. The CDNF knockout (*Cdnf*^−/−^) mice subjected to collagenase induced-ICH were used to determine the role of endogenous CDNF in ICH outcomes.

We showed that exogenous administration of fluorescent-labeled rhCDNF penetrates and is distributed to striatal parenchyma to exert its function in ICH rats. The neuroprotection study with rhCDNF was designed to see whether delayed i.c.v. administration after ICH restores ICH-induced behavioral deficits in rats (a controlled laboratory experiment with a prespecified hypothesis), as measured by mNSS and cylinder test. None of the animals that survived the surgeries were excluded, and there were no outliers removed. The gross distribution of hemorrhagic lesions was determined by morphometric measurement and T2-weighted images of MRI. Brain edema and BBB disruption were evaluated by signal intensity of T2-weighted images of MRI and Evans blue assay, respectively. Apoptotic cells were measured by a TUNEL assay.

The neuroprotective effects of rhCDNF might mediate immune modulation via accelerating cleanup of blood components and mitigate hematoma-induced secondary brain injury. Considering that the value E, the degree of freedom of analysis of variance (ANOVA), should lie between 10 and 20, a separate set of animals, treated with saline or rhCDNF (*n* = 8–10 per group), was used in the assessment of lesioned volume and behavioral assays. To investigate the spatiotemporal expression of scavenger receptors, including CD163, CD36 and CD91 in the hemorrhagic striatum, immunofluorescent and immunoblotting analysis of brain tissue were performed (*n* = 20 in total; *n* = 4–6 per group). Dose-response of rhCDNF was tested in a separate experiment (*n* = 5 per group). In addition, the mechanistic study was performed to determine whether rhCDNF treatment maintains ER or oxidative homeostasis, which is implicated in GSK-3β activity and the Nrf2/HO-1 pathway. The immune modulation study with CDNF was designed to characterize inflammatory responses, including pro-inflammatory and anti-inflammatory mediators, at different time points and to provide isolated CD11b^+^ cells for transcriptome analysis of hemorrhagic striatum. The rats were allocated into sham+ saline, ICH + saline and ICH + rhCDNF on days 1, 3, and 7 post-ICH induction. The third neuroprotective study with systemic administration of rhCDNF (*n* = 9) and saline (*n* = 6) was designed to reveal whether intravenous delivery of rhCDNF after ICH also induced expression of scavenger receptors in the hemorrhagic striatum to accelerate lesion resolution and reversal of hemorrhagic injury-induced behavior deficits in rats. Rats were allocated into two treatment groups at 1 h after collagenase injection surgery.

### Animals

All mouse experiments were performed in accordance with the 3R principles of the EU directive 2010/63/EU on the care and use of experimental animals, and local laws and regulations (Finnish Act on the Protection of Animals Used for Scientific or Educational Purposes (497/2013) and Government Decree on the Protection of Animals Used for Scientific or Educational Purposes (564/2013). The generation of CDNF knockout *Cdnf*^*−*^^/−^ mice has been described previously [22]. The CDNF gene was interrupted by inserting a puromycin/tka cassette flanked by Frt and LoxP recombination sites into the intron between exon 1 and exon 2. Heterozygous *Cdnf*^Pu/+^ mice were then crossed to transgenic mice ubiquitously expressing CaqFlp recombinase to remove the puromycin cassette, resulting in the ablation of *Cdnf* full-length transcript and protein.

Rat experiments were carried out in accordance with the National Institutes of Health Guide for the Care and Use of Laboratory Animals, and the Affidavit approved experimental protocols of Approval of Animal Use Protocol Board Buddhist Tzu Chi General Hospital (Approval No. IACUC 107-04). Animals were housed under a 12-h light/dark cycle with free access to food and water. Utmost efforts were made to minimize suffering and number of animals used. Experimental procedures followed the guidelines for the “Guide for the Care and Use of Laboratory Animals” (National Institutes of Health publication, 1996), local laws and regulations.

### Production of recombinant CDNF proteins (Drugs)

In i.c.v. studies recombinant human CDNF (rhCDNF), produced in mammalian cells, was purchased from Icosagen (cat. nr. P-100-100, Tallinn, Estonia). In i.v. studies and in vitro phagocytosis study rhCDNF was produced in e. coli and provided by Herantis Pharma. Biological activity of rhCDNF was tested on cultured sympathetic and dopamine neurons.

### Intracerebral Hemorrhage Model

Mice and rats were anesthetized with 3% isoflurane/O_2_ mixture (Sigma-Aldrich, St. Louis, MO, USA) for induction and maintenance with 2% isoflurane/O_2_ mixture throughout surgical procedures.

#### Mouse ICH model

Three-month-old male wild-type (Wt) or *Cdnf*^−/−^ mice (25–30 g) were used to perform the microinfusion of bacterial collagenase VII-S (0.075U in 0.5 μl sterile saline) into the right striatum of the mouse (0.2 mm anterior, 2.0 mm right, 3.7 mm ventral to bregma at the skull surface) over 10 min. The needle was kept in place for another 10 min to prevent backflow. The burr hole was sealed with bone wax. Mice were allowed to recover in separate cages at room temperature.

#### Rat ICH models

Twelve-week-old male Sprague-Dawley rats (300–350 g) were performed by micro-infusion of bacterial collagenase VII-S (0.23 U in 1 μl sterile saline) into the right striatum of the rat (0.0 mm anterior, 3.0 mm right, 5.0 mm ventral to bregma at the skull surface) over 10 min or 100 μl autologous blood infusion over 20 min into the striatum (0.0 mm posterior, 3.0 mm right, 5.0 mm ventral to bregma at the skull surface). The needle was kept in place for an additional 10 min to prevent backflow. The burr hole was sealed with bone wax. After recovery from anesthesia, the animals were returned to their home cage with free food and water intake.

### Assessment of hemorrhagic volume and lesion volume

Hemorrhagic lesion volume was analyzed by morphometric measurement as described. After rats or mice were decapitated, brains were removed and sliced into 2-mm-thick sections using an acrylic brain matrix. Digital photographs of serial slices were taken, and hemorrhagic lesion volume in the striatum was computed using an image analysis program (Image J, NIH, USA). The total lesion volume (mm^3^) was calculated by multiplying the lesion area in each section by the distance between sections. Hemoglobin content determined by spectrophotometric measurement is a well-defined manifestation of the hemorrhagic volume after ICH [23].

### Magnetic resonance imaging (T2-weighted & diffuse weighted image)

Magnetic Resonance Imaging (MRI) studies were performed with a 4.3 cm diameter surface coil on a 3 T MRI (Signa HDx, GE healthcare Milwaukee, WI, USA). The intensity of T2WI was processed and quantitated with a GE workstation analysis software package (version 4.2, GE Healthcare, Fairfield, USA). A ratio of the signal intensity-time curve in a 9 mm^2^ region of interest (ROI) of the hematoma (right) to the contralateral striatum (left) was semi-segmental and calculated by ITK-SNAP software (version 2.2, University of Pennsylvania, Philadelphia, USA). T2-weighted images were acquired using a rapid acquisition with relevant parameters: repetition time (TR)/echo time (TE): 3100/60 ms, matrix size = 256 × 256, a field of view (FOV) = 40 × 40 mm, slice thickness = 1.0 mm; encompassing the whole brain. Diffused weighted MRI (DWI) images were acquired using a spin-echo pulse sequence with the following acquisition parameters set as TR/TE: 1500/37.7 ms; matrix size = 256 × 256; field of view = 40 × 40 mm; slice thickness = 1.0 mm; *b* values 18, 320, 1102 s/mm^2^.

### BBB disruption with Evans blue extravasation

The integrity of BBB was evaluated with a modified Evans blue extravasation method before sacrifice [23]. Briefly, 70 h post-ICH, rats were anesthetized with sodium pentobarbital (50 mg/kg, i.p.) and infused via the right femoral vein with 37 °C Evans blue dye (2% in 0.9% normal saline, 4 ml/kg) over 5 min. Two hours later, the rats were perfused with 300 ml normal saline to wash out any remaining dye in the blood vessels, and then the brains were removed and sectioned to 2 mm thickness with a rodent brain matrix. Coronal brain sections were taken starting at +2 mm and ending at −2 mm from bregma. BBB permeability was evaluated in the ipsilateral and contralateral striatum, and cerebellum. The cerebellum was used as an internal control. Each portion was weighed immediately and placed in 1 ml of 0.9% normal saline for homogenization of the sample. For protein precipitation, 1 ml of 60% trichloroacetic acid solution was added and vortexed for 2 min. Subsequently, the mixture was cooled for 30 min and centrifuged (1500 × *g* at 4 °C) for another 30 min. The absorbance of Evans blue in the supernatant was then measured with a spectrophotometer (Molecular Devices OptiMax, USA) at 610 nm. Dye concentration was expressed as μg/g of tissue weight and calculated from a standard curve obtained from known amounts of dye.

### Functional neurobehavioral tests

Assessment of neurological abnormalities by a modified Neurological Severity Score (mNSS) test (normal score, 0; maximal deficit score, 18) and cylinder test were carried out as described [23, 24]. Evaluation was performed by an investigator blinded to the experimental treatment scheme. The mNSS is a composite test of motor, sensory, and balance functions. The assessment was performed on day 1 pre- and on days 1, 3, and 7 post-ICH. In the cylinder test, the animal is placed inside a transparent vertical tube with a diameter of 35 cm, and its movement is video recorded for 5 min. The number of first front paw touches to the inner wall of the tube, after rising on hind limbs, is counted.

### Immunoblotting

The area of the hemorrhagic lesion composed of hematoma core and peri-hematoma striatum extending 1 mm from the hematoma border was collected and rapidly frozen in liquid nitrogen at −80 °C until further analysis. Briefly, equal amounts of protein (20 μg) were loaded onto 10% sodium dodecyl sulfate-polyacrylamide gel (SDS-PAGE) gel and then electrophoresed, followed by blotting of protein onto a polyvinylidene difluoride (PVDF) membrane (Immobilon-P PVDF membrane, Millipore, Bedford, MA) blocked with 5% non-fat milk in 0.05% Tween-Tris-buffered saline. The membrane was probed with primary antibodies overnight at 4 °C with gentle rotation. Primary antibodies and concentrations were used as follows: hCDNF (1:250, Sigma-Aldrich, HPA044587), β-actin (1: 10000, Sigma-Aldrich, A5316), CD163 (1:250; Abcam, ab87099, ab182422), CD91 (1:1000, Abcam, ab92544), CD36 (1:500, Novus,NB400-144), Nrf2 (C-term, 1:500, Cayman; 1:250, Abcam, ab137550), Histone 3 (1:2000; GeneTex, GTX122148), HO-1 (1:500, Abcam, ab13243), GAPDH (1:1000, Abcam, ab181602), GRP78 (1: 1000, Cell Signaling Technology, 3183S), ATF4 (1:500, Proteintech, 10835-1-AP), ATF6α (1:500, Santa Cruz, H2120), CHOP (1: 2000, Cell Signaling Technology, 2895S), phosphor-GSK3β (Ser 9, 1:1000, Cell Signaling Technology, 9336S), phosphor-GSK3β (Y216, 1: 1000, BD, 612313), GSK3β (1:2000, Cell Signaling Technology, 9315 S), phosphor-Akt (1:500, Cell Signaling Technology, 9217S), Akt (1:1000, Cell Signaling Technology, 9272 S), phosphor-ERK1/2 (1:2000, Cell Signaling Technology), ERK1/2 (1:1000, Cell Signaling Technology), phosphor-p38 (1:1000, Cell Signaling Technology, 9211S), p38 (1:1000, Cell Signaling Technology, 9212S), phosphor-JNK (1:500, Cell Signaling Technology, 9251S), JNK (1:1000, Cell Signaling Technology, 9252 S). Following washing and incubation with respective secondary antibodies (AP307P and AP308P; EMD Millipore, Billerica, MA, 1: 20,000) for 1 h at room temperature, membranes were then reacted with chemiluminescent ECL Plus Western blotting detection system (RPN2133; Amersham Biosciences, Little Chalfont, UK). Bands were detected by exposure to X-ray films (34091; Kodak, Rochester, NY). Band intensities were quantified using densitometric analysis (GS-800 Calibrated Densitometer, Bio-Rad, Hercules, CA) and calculated as the optical density × area of the band.

### TUNEL assay

TUNEL staining was assayed using In Situ Cell Death Detection kit, POD (Roche), according to the manufacturer’s instructions. TUNEL signal was detected with FITC-labelled secondary antibody to streptavidin. Cell nuclei were counterstained with DAPI. Apoptotic/necrotic cells were counted by scoring TUNEL positive/DAPI cells around the peri-hematoma region.

### Immunohistochemical staining

Animals were deeply anesthetized with pentobarbital (50 mg/kg, i.p.) and transcardially perfused with cold 0.1 M phosphate buffer saline followed by cold 4% paraformaldehyde in 0.1 M phosphate-buffered saline. Brains were removed and immersed in 4% paraformaldehyde for 24 h and 30% sucrose for another 24 h. Coronal brain slices (20 μm thickness) were cut. Slices were collected at +1.0, 0.0, and −1.0 mm (center of the hemorrhagic lesion) anterior and posterior to bregma using a cryostat (Leica CM 1900) and processed for the staining and counting of marker-specific cells. The following primary antibodies were used, with the dilution rates and retrieval methods indicated in parentheses. Antibodies against CD91(1:500, Abcam, ab92544), CD36 (1:500, Novus, NB400-144), CD163 (1:250, Abcam, ab182422), CD11b (1:200, AbD Serotec; 1:250, Abcam, ab1211), CD68 (1:500; Bio-Rad, MCA341R) and Arginase-1 (1:500, Santa Cruz, sc-271430) were used as microglia/macrophage markers. Anti-RECA antibody (1:200, Abcam, ab9774) was used as an endothelial marker. Anti-GFAP antibody (1:500, NOVUS, NBP-05197) was used as a glial marker, and anti-NeuN antibody (1:1000; Chemicon, MAB377) was used as a neuron marker.

### Reverse transcription and quantitative polymerase chain reaction (qPCR)

Total RNA was extracted from rat or mice striatum using the Total RNA Isolation Kit (tissue, GeneDireX, Taipei, Taiwan). cDNA was synthesized using the SuperScript® III First-Strand Synthesis System for RT-PCR (Invitrogen, Carlsbad, CA, USA). RNA Purity and quantification were checked using SimpliNano™ - Biochrom Spectrophotometers (Biochrom, MA, USA). One-step real-time RT-PCR analysis was performed to determine gene expression (Luminaris Color HiGreen qPCR Master Mix, K0391; Thermo Fisher Scientific, Waltham, MA). Real-time PCR primers listed in table S1 that were used in this study included: Cdnf, Gapdh, Bip, sXbp-1, Atf4, Chop, Homx1, Actin, ApoE, Gpnmb, Cd63 & Ube2i (Supplement Table 1). Quantitative real-time PCR was performed in a reaction mixture containing cDNA, 200 nM of specific primers, and Maxima SYBR Green/ROX qPCR Master Mix (Fermentas, Waltham, MA, USA). PCR amplification was performed in a QuantStudio 5 Real-Time PCR System (Applied Biosystems, Waltham, MA, USA). The following PCR conditions were used: 50 °C for 2 min, 95 °C for 10 min, 40 cycles of 95 °C for 15 s, 60 °C for 1 min, and 60 °C for 30 s. The ΔΔCt method was used for data analysis. mRNA expression levels were measured in triplicate and normalized to expression levels of Gapdh, actin, or Ube2i in the same samples.

### Human CDNF ELISA after systemic administration

Intracerebral Hemorrhage model was performed and one dose of vehicle or rhCDNF was administered intravenously 60 min after collagenase injection. Blood samples were collected 6 and 24 h post-intravenous (30 μg) rhCDNF. After collection of whole blood, it was allowed to blot at room temperature for 30 min. Then, supernatant was collected by removing the clot by centrifuging 2000 × *g* for 10 min in a refrigerated centrifuge. Supernatants (serum) were used for rhCDNF detection using a human CDNF Elisa Kit (ab260071, Abcam) according to the manufacturer’s protocol.

### RNA-sequencing from CD11b^+^ cells isolated from the hemorrhagic striatum of rats

Rat striatal microglial cells were isolated as described [25] by using the gentleMACS^TM^ (Miltenyi Biotec, Germany) procedure. Briefly, isolated ipsilateral striatum tissue (400 mg) at day 3 post-ICH injury was dissociated using a Neural Tissue Dissociation kit (Miltenyi Biotec) and gentleMACS Dissociator (Miltenyi Biotec, Cat. # 130-049-601, Germany). After dissociation, cells were suspended in 0.5 % BSA in PBS and incubated with Myelin Removal Beads II (1:10, Miltenyi Biotec) for 15 min at 4 °C, then washed and resuspended in 0.5% BSA in PBS and filtered through an LS column (Miltenyi Biotec) using a Quadro-MACS Separator (Miltenyi Biotec). The total effluent was collected and resuspended in 0.5% BSA with 2 mM EDTA in PBS. Cells were incubated at 4 °C for 10 min with rat anti-CD11b: FITC antibody (AbD Serotec). Cells were washed, resuspended in 0.5% BSA with 2 mM EDTA in PBS, and incubated with anti-FITC MicroBeads (Miltenyi Biotec) for 15 min at 4 °C. Cells were washed and resuspended in 0.5% BSA with 2 mM EDTA in PBS. The cell suspension was applied to a LS column placed on a QuadroMACS Separator. Finally, we isolated the CD11b positive cells and centrifuged the suspension. Cell suspensions were subsequently counted for further RNA extraction and RNA-Sequencing analysis. 500 ng of total RNA was ribo-depleted using Illumina’s mammalian Ribo-Zero Magnetic Gold Kit HMN/Mouse/Rat (MRZG12324). Library preparation was completed on ribo-depleted RNA using the NEBNext Ultra Directional RNA Library prep kit (NEB, #E7420L) using 8 cycles of PCR amplification and indexed using single (i7) indexing. Indexed library preps from each sample were then pooled and sequenced with 75SE reads at a pool concentration of 1.5 pM and 2.6 pM on the NextSeq 500 using respectively a NextSeq High Output 75 cycle flow cell and a NextSeq Mid Output 150 cycle flow cell (both Illumina). bcl2fastq2 Conversion Software (v2.20.0.422) was used to convert BCL files to FASTQ file format and demultiplex samples. Sequenced reads were trimmed for adaptor sequences and masked for low-complexity or low-quality sequence using Trimmomatic. Trimmed reads were mapped to Rnor_6.0 (GCA_000001895.4) reference genome using STAR aligner (2.6.0c). Counts per gene were calculated using featureCounts tool from Subread package (v1.6.4). The proportion of reads mapped to the human reference genome ranged from 65 to 81.9% in total for all samples. Differentially expressed genes were identified using CLC Genomics Workbench (version 21), and differences with a FDR *p* value < 0.05 were considered as significant. For the comparison between different groups, transcript per million (TPM) were calculated based on a negative binomial Generalized Linear Model (GLM) (Supplement data 3). The raw data for this project are available at the Gene Expression Omnibus under accession number GSE216547. GO enrichment analysis of DEGs was conducted using clusterProfiler (v3.10.1) (Supplement data 4) [26].

### In vitro erythrophagocytosis of BV2 cell lines and primary microglial cells

Murine BV-2 microglial cells were a gift from Dr. Mei-Jen Wang and maintained in Dulbecco’s modified Eagle’s medium (DMEM) containing 5% fetal bovine serum (FBS), 2 mM l-glutamine, 100 µg/ml streptomycin, and 100 U/ml penicillin (all from Gibco; Thermo Fisher Scientific, Inc., Waltham, MA, USA) at 37 °C under humidified 95% O_2_ and 5% CO_2_. BV-2 microglial cells were confirmed to be free of mycoplasma contamination. Cells were seeded into 24-well plates at a density of 1 × 10^5^/well and maintained at 37 °C under humidified 95% O_2_ and 5% CO_2_. Red blood cells (RBCs) were isolated from whole blood of donor mice, heated-shocked at 56 °C for 6 min to induce phosphatidylserine expression and labeled with lipophilic PKH-26 fluorescent probe (Sigma-Aldrich). The PKH-26-labeled RBCs for in vitro studies were counted and fed to BV2 cells at 1:30 ratio of BV2 cells and RBCs. After incubation at 37 °C for 6 h, the unengulfed RBCs were removed by washing with Dulbecco’s PBS 3 times followed by staining BV2 cells with CD11b. Engulfed RBCs were observed by fluorescent microscopy. Phagocytosis index of BV2 cell lines was calculated according to the following formula: Phagocytic index = (number of RBC-engulfed BV2 cells / total number of counted BV2 cells) × 100.

As described previously, primary microglial cells were isolated using p1-p2 rat pups [27]. Briefly, neonatal pups were sacrificed, and the ventral mesencephalic tissue was isolated by a mild mechanically trituration. The isolated cells (5 × 10^7^) were seeded in 150 cm^2^ culture flasks in DMEM containing 10% FBS, 50 U/ml penicillin, and 50 mg/ml streptomycin for 14 days. The loosely adherent microglia were harvested by shaking (180 rpm) the flasks for 2 h at 37 °C. Total 2 × 10^5^ cells per well were plated into a 24-well plate and incubated 24 h before drug treatments or phagocytosis assay. The RBCs from whole blood of donor rat were isolated using Ficoll-paque PLUS (GE Healthcare, USA). Fluorescent dye 5 [6]-carboxyfluorescein diacetate (CFDA; Molecular Probes, Eugene, OR, USA) were used to label purified RBCs for in vitro studies. The CFDA-labeled RBCs for in vitro studies were counted and fed to cells at a 1:10 ratio of primary microglial and RBCs. After incubation at 37 °C for 6 h, the unengulfed RBCs were removed by washing with Dulbecco’s PBS 3 times, then staining primary microglial cells with CD11b. The engulfed RBCs were observed by fluorescent microscopy. In parallel, primary microglial cells containing phagocytosed CFDA-RBCs were lysed in distilled water. The fluorescence intensity in the supernatant from the cell lysates was measured using a fluorometer with a 492/517 nm filter set. Phagocytosis index was calculated according to the following formula: Phagocytic index = (Fluorescence intensity of RBC-engulfed microglia ­ mean fluorescence intensity of microglia)/(mean fluorescence intensity of PBS-treated RBC-engulfed microglia – mean fluorescence intensity of microglia) × 100% [28].

### Statistical analysis

All graphs and statistics were performed in GraphPad Prism 9.0. The lesion volume, TUNEL assay, and qPCR results were analyzed using the two-tailed Student’s *t*-test. The statistical comparisons among multiple groups were made using one-way ANOVA, and multiple time points by two-way ANOVA were followed by Bonferroni or Dunnett’s multiple-comparisons post hoc test. In all instances, n refers to the number of animals in a particular group. A *p*-value of <0.05 is considered statistically significant.

## Results

### CDNF expression in the hemorrhagic striatum decreased at the early stage of ICH insult

**Fig. 1:**
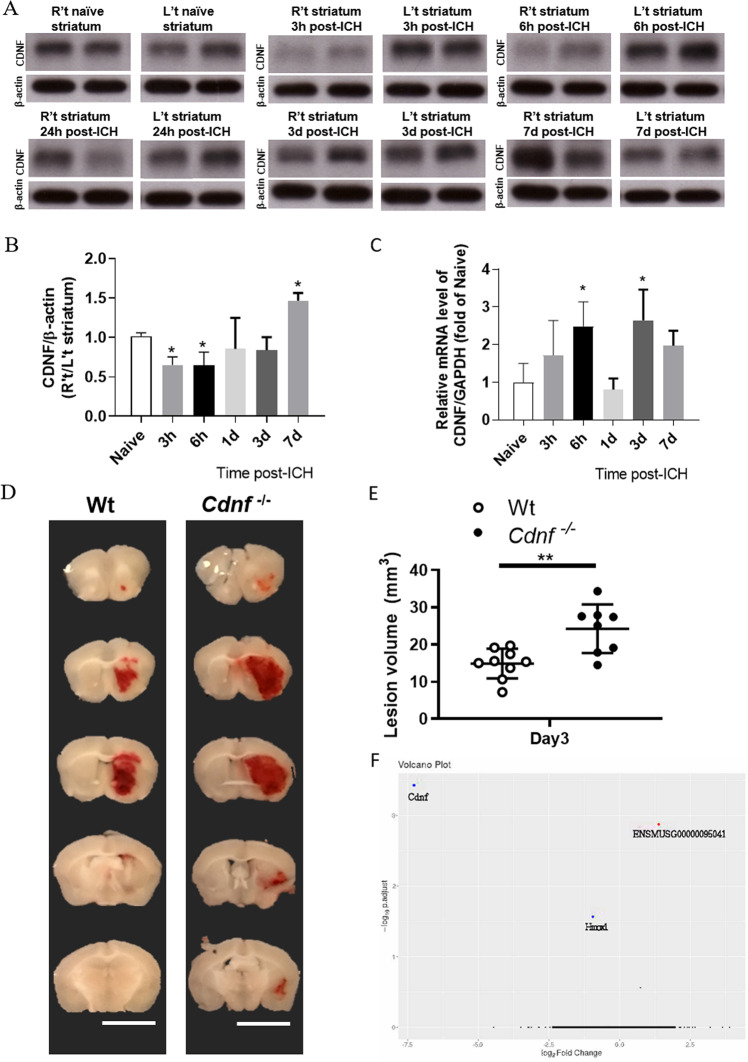
Endogenous CDNF affects the hemorrhagic lesion after ICH. **A** Photograph of representative films demonstrating temporal changes in CDNF protein in the naive striatum and ICH-affected striatum, at 3 h to 7 days post-ICH in SD rats, which were assessed using Western blotting. **B** Bar graph showing the relative levels of CDNF protein in the striatum of naïve rats and rats at 3 h, 6 h, 24 h, 72 h, and 7 days after ICH. Data were analyzed as repeated measures by one-way ANOVA followed by Bonferroni corrections (*n* = 4/time point). **C** Bar graph showing time course of CDNF mRNA levels in hemorrhagic striatum at 3 h to 7 days after ICH in SD rats. Data were analyzed as repeated measures by one-way ANOVA followed by Bonferroni corrections (*n* = 3/time point). **D** Representative coronal sections (1 mm thickness) showing brain hemorrhagic areas of WT and *Cdnf*^−*/−*^ mice killed 3 days after ICH. **E** Lesion volume on days 3 (*n* = 7–8, each group) post-ICH was determined by morphometric measurement. Data were analyzed as two-tailed Student’s *t*-test. **F** Volcano plot of gene expression profiles in hemorrhagic striatum collected after collagenase-induced ICH in WT and *Cdnf*^*-/-*^ mice, showing distribution of significance [−log10(adjusted *P* value)] vs. fold change [log2(fold change)] for all genes. The blue dots indicate downregulated genes (fold change < −1.5, adjusted *P* val <0.05), the red dots indicate upregulated genes (fold change >1.5, adjusted *P* val <0.05), and the black dots indicate genes with no significant change post-ICH. *P < 0.05 by multiple comparisons using the Holm-Šidák method. Mean ± SEM is shown. Scale bars: 5 mm.

CDNF expression is widely distributed in the cerebral cortex, hippocampus, and striatum of the rodent brain [13] and the protein is principally located in the ER lumen of neurons or glia. In pathological conditions in which ER calcium is misbalanced due to the accumulation of misfolded proteins, CDNF can be secreted and may have paracrine effects in the injured tissue [14]. Using collagenase-induced ICH in rats, we investigated whether the expression of CDNF in peri-hematoma tissue was affected. Immunoblotting analysis showed that CDNF expression in the peri-hematoma striatum decreased within 6 h of ICH onset, persisted at a sub-marginal level for at least 3 days, and then increased at 7 days (Fig. 1A, B; *F* (5, 28) = 4.151, *p* = 0.006, One-way ANOVA Holm-Šidák multiple comparison test, Table 1a.1). On the other hand, mRNA analysis showed a transient increase of CDNF transcript at 6 h and 3 days (Fig. 1C; *F* (5, 17) = 5.208, *p* = 0.0044, One-way ANOVA Holm-Šidák multiple comparison test, Table 1a.2) after ICH insult. This discrepancy between CDNF protein and *mRNA* levels could reflect a compensatory mechanism for retaining steady intracellular CDNF levels in the hemorrhagic striatum. These results also suggest that downregulation of CDNF was not due to a decrease in CDNF biogenesis during the acute phase of ICH injury. It may be attributable to aberrant CDNF trafficking and secretion or pathological degradation of the CDNF protein following ICH insult.

**Table 1:** Statistical table.

Dataset	Data structure	Type of test	Power
**a.1** Fig. 1B	Normal distribution	Ordinary one-way ANOVA Holm-Šidák multiple comparison test	*F* (5, 28) = 4.151, *p* = 0.006 Naive vs. 3 h: Padj:0.0278 Naive vs. 6 h: Padj:0.0162 Naive vs. 1d: Padj:0.294 Naive vs. 3d: Padj:0.244 Naive vs. 7d: Padj:0.04
**a.2** Fig. 1C	Normal distribution	Ordinary one-way ANOVA Holm-Šidák multiple comparison test	*F* (5, 17) = 5.208, *p* = 0.0044 Naive vs. 3 h: Padj:0.2933 Naive vs. 6 h: Padj:0.0298 Naive vs. 1d: Padj:0.6974 Naive vs. 3d: Padj:0.0189 Naive vs. 7d: Padj:0.1802
**a.3** Fig. 1E	Normal distribution	Paired *t*-test	*t*(6) = 3.423, *p* = 0.01
**b.1** Fig. 2C	Normal distribution	Ordinary two-way ANOVA Šidák multiple comparison test	CDNF therapy: *F* (1, 31) = 8.574, p = 0.0063 Interaction: *F* (1, 31) = 6.910, *p* = 0.0132
**b.2** Fig. 2E	Normal distribution	paired *t*-test	*t*(6) = 5.482, *p* = 0.0015
**b.3** Fig. 2G	Normal distribution	Ordinary two-way ANOVA Šidák multiple comparison test	CDNF therapy: *F* (1, 21) = 12.04, *p* = 0.0023
**b.4** Fig. 2I	Normal distribution	Ordinary two-way ANOVA Šidák multiple comparison test	Interaction: *F* (1, 14) = 9.895 *p* = 0.0072 Ipsilateral striatum: t(14)=3.319, *p* = 0.01 Contralateral striatum: *t*(14) = 1.252, p = 0.41
**b.5** Fig. 2J	Normal distribution	Ordinary two-way ANOVA Bonferroni’s multiple comparison test	CDNF therapy: *F* (1, 65) = 21.21, *p* < 0.0001 pre-op: *p* > 0.99 D1: *P* = 0.005 D3: *P* = 0.001 D7: *P* = 0.1458
**b.6** Fig. 2k	Normal distribution	Ordinary two-way ANOVA Bonferroni’s multiple comparison test	CDNF therapy: F (1, 48) = 29.76, *p* < 0.0001 pre-op: *p* > 0.99 D1: *P* = 0.0006 D3: *P* = 0.0022 D7: P = 0.0128
**b.7** Fig. 2M	Normal distribution	Paired *t*-test	*t*(16) = 2.925, *p* = 0.0099
**c.1** Fig. 3E	Normal distribution	Ordinary two-way ANOVA Bonferroni’s multiple comparison test	CDNF therapy: *F* (1, 32) = 5.026, *p* = 0.03 D1: *P* = 0.004 D3: *P* > 0.99
**c.2** Fig. 3G	Normal distribution	Ordinary two-way ANOVA Šidák multiple comparison test	CDNF therapy: *F* (1, 17) = 10.95, *p* = 0.004 D1: *P* = 0.001 D3: *P* = 0.85
**c.3** Fig. 3H	Normal distribution	Ordinary two-way ANOVA Bonferroni’s multiple comparison test	CDNF therapy: *F* (1, 12) = 15.23, *p* = 0.002 D1: *P* = 0.002 D3: P = 0.53
**c.4** Fig. 3I	Normal distribution	Ordinary two-way ANOVA Bonferroni’s multiple comparison test	CDNF therapy: *F* (1, 12) = 6.799, *p* = 0.02 D1: *P* = 0.01 D3: P = 0.67
**c.5** Fig. 3J	Normal distribution	Ordinary two-way ANOVA Bonferroni’s multiple comparison test	CDNF therapy: *F* (1, 20) = 1.099, *p* = 0.93 D1: *P* = 0.69 D3: P = 0.64
**d.1** Fig. 4B	Normal distribution	Ordinary two-way ANOVA Bonferroni’s multiple comparison test	CDNF therapy: *F* (1, 18) = 14.37, *p* = 0.001 6 h: *P* = 0.02 24 h: *P* = 0.01 72 h: *p* > 0.99
**d.2** Fig. 4C	Normal distribution	Ordinary two-way ANOVA Bonferroni’s multiple comparison test	CDNF therapy: *F* (1, 26) = 8.826, *p* = 0.006 6 h: P = 0.004 24 h: P = 0.03 72 h: *p* = 0.07
**d.3** Fig. 4E	Normal distribution	Ordinary two-way ANOVA Bonferroni’s multiple comparison test	CDNF therapy: *F* (1, 12) = 15.28, *p* = 0.002 6 h: *P* = 0.005 24 h: *p* = 0.11
**d.4** Fig. 4F	Normal distribution	Ordinary two-way ANOVA Bonferroni’s multiple comparison test	CDNF therapy: *F* (1, 12) = 16.37, *p* = 0.002 6 h: *P* = 0.01 24 h: *P* = 0.07
**d.5** Fig. 4G	Normal distribution	Ordinary two-way ANOVA Bonferroni’s multiple comparison test	CDNF therapy: F (1, 10) = 18.24, *p* = 0.002 6 h: *P* = 0.75 24 h: *P* < 0.001
**d.6** Fig. 4H	Normal distribution	Ordinary two-way ANOVA Bonferroni’s multiple comparison test	CDNF therapy: *F* (1, 8) = 14.58, *p* = 0.005 6 h: *P* = 0.64 24 h: *P* = 0.005
**d.7** Fig. 4I	Normal distribution	Ordinary two-way ANOVA Bonferroni’s multiple comparison test	CDNF therapy: *F* (1, 18) = 11.41, *p* = 0.003 6 h: *P* < 0.001 24 h: *P* > 0.99
**d.8** Fig. 4J	Normal distribution	Ordinary two-way ANOVA Bonferroni’s multiple comparison test	CDNF therapy: *F* (1, 18) = 0.275, *p* = 0.61 6 h: *P* = 0.65 24 h: *P* = 0.92
**e.1** Fig. 5B	Normal distribution	Ordinary two-way ANOVA Bonferroni’s multiple comparison test	CDNF therapy: *F* (1, 17) = 29.03, *p* < 0.001 medial peri-hematoma region: *p* = 0.0012 lateral peri-hematoma region: *p* = 0.0063
**e.2** Fig. 5D	Normal distribution	*t*-test	*t*(15) = 3.096, *p* = 0.0074
**e.3** Fig. 5I	Normal distribution	Multiple unpaired *t*-test using parametric test	ApoE: *t*(12) = 3.51, *p* = 0.004 Gpnmb: *t*(12) = 2.87, *p* = 0.013 CD63: *t*(9) = 2.47, p = 0.037
**f.1** Fig. 6C	Normal distribution	Ordinary one-way ANOVA Dunnett’s multiple comparison test	CDNF therapy: F (2, 11) = 6.222, *p* = 0.0156 Vehicle vs. rhCDNF (0.5 μg/ml): *p* = 0.054 Vehicle vs. rhCDNF (1 μg/ml): *p* = 0.0096
**f.2** Fig. 6D	Normal distribution	Ordinary one-way ANOVA Dunnett’s multiple comparison test	CD163: CDNF therapy: *F* (2, 6) = 15.16, p = 0.0045; Vehicle vs. rhCDNF (1 μg/ml): *p* = 0.0031 CD36: CDNF therapy: *F* (2, 6) = 5.56, *p* = 0.043; Vehicle vs. rhCDNF (1 μg/ml): *p* = 0.042 HO-1: CDNF therapy: F (2, 8) = 5.085, *p* = 0.0376; Vehicle vs. rhCDNF (0.5 μg/ml): p = 0.0356; Vehicle vs. rhCDNF (1 μg/ml): *p* = 0.0424
**f.3** Fig. 6F	Normal distribution	paired *t*-test	t(5) = 2.972; *p* = 0.031
**g.1** Fig. 7C	Normal distribution	*t*-test	*t*(5) = 2.707, *p* = 0.0424
**g.2** Fig. 7D	Normal distribution	Ordinary two-way ANOVA Bonferroni’s multiple comparison test	CDNF therapy: *F* (1, 35) = 18.93, *p* < 0.00 1D1: *P* = 0.0037 D3: *p* = 0.003
**g.3** Fig. 7E	Normal distribution	Ordinary two-way ANOVA Bonferroni’s multiple comparison test	CDNF therapy: *F* (1, 18) = 29.41, *p* < 0.001 6 h: *P* < 0.001 24 h: *p* > 0.99
**g.4** Fig. 7G	Normal distribution	Multiple unpaired *t*-test with Holm-Šidák multiple comparison test	hCDNF: *t*(10) = 5.47, *p* < 0.0001 CD163: *t*(13) = 3.2, *p* = 0.007 CD91: *t*(6) = 0.217, *p* = 0.084 Cell surface CD36: *t*(8) = 2.77, *p* = 0.02 Intracellular CD36: t(8) = 5.18, *p* < 0.001

### Loss of CDNF, decreasing Hmox1 transcription at the early time point, increases hematoma volume in a mouse model of collagenase-induced ICH

To determine the role of endogenous CDNF in ICH outcomes, wild-type (WT) and CDNF knockout (*Cdnf*^−/−^) adult male mice [22] were subjected to collagenase injection. The absence of CDNF protein and specificity of the antibody were verified by Western blot from striatal tissue of *Cdnf*^−/−^ mice brain (Fig. S1A). The lesion volume, manifesting as hematoma deposition, at 3 days post-ICH was significantly larger in *Cdnf*^−/−^ mice than in WT mice (Fig. 1D, E; *t*(6) = 3.423, *p* = 0.01, paired *t*-test, Table 1a.3), suggesting that endogenous CDNF plays a role in coping with collagenase-induced hemorrhagic injury. To further investigate the biological effects of CDNF during hematoma accumulation, we performed RNA sequencing (RNAseq) from lesioned striatum 6 h after collagenase injections (Fig. S1B). Principal component analysis indicated segregation of the samples into two distinct groups, supporting reproducibility of our methodology (Fig. S1C). Our data show that only two transcripts were differentially expressed (FC > 1.5) compared to the WT group, *Cdnf* and Heme Oxygenase 1 (*Hmox1)*, both of which were downregulated (Fig. 1F and Fig. S1D). Quantitative polymerase chain reaction (qPCR) analysis verified that in *Cdnf*^−/−^ mice, expression of *Hmox1* was significantly down-regulated (Fig. S1D) in response to ICH insult. Although CDNF was shown to regulate UPR initiated by transducers IRE1α, PERK and ATF6 in the pathological condition [29], we found that mRNA levels of UPR markers *Grp78*, spliced *Xbp1*, *Atf4*, and *Chop* were not significantly different between WT and *Cdnf*^*−/−*^ mice after intracerebral hemorrhage (Fig. S1E). Overall, these findings suggest that loss of CDNF decreases mRNA levels of *Hmox1* at the early stage of ICH, but increases hematoma accumulation at 3 days post hemorrhagic stroke.

### CDNF treatment mitigates brain injury and improves recovery of neurological function following ICH

**Fig. 2:**
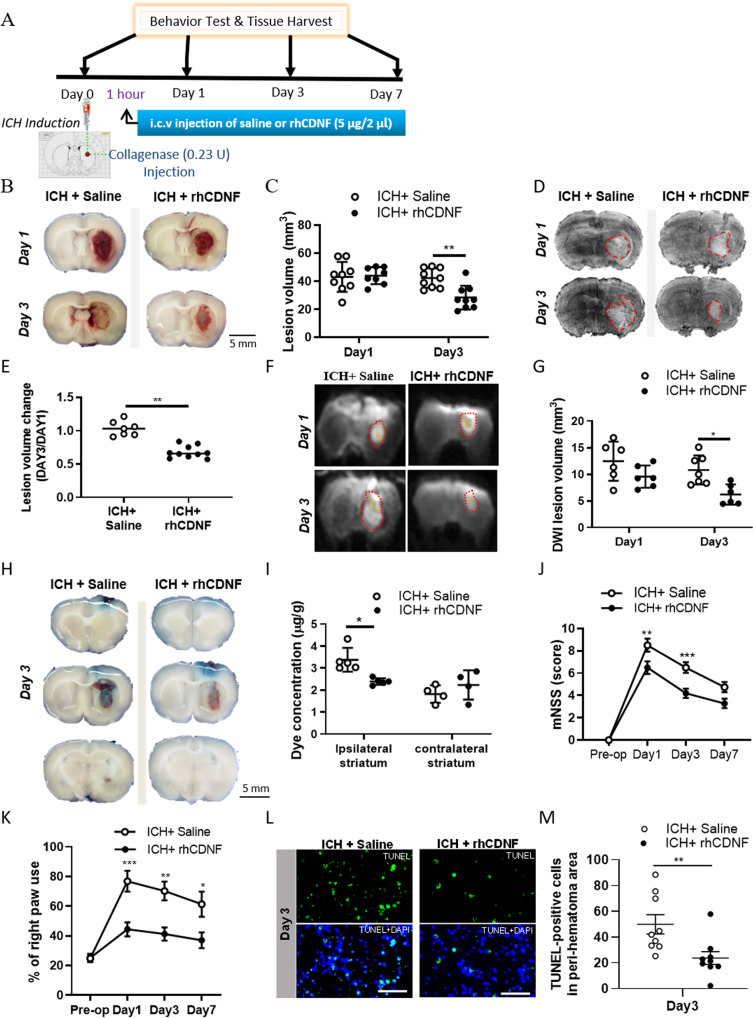
Effects of rhCDNF treatment on hemorrhagic lesion, brain edema, integrity of BBB neurobehavioral outcomes and cellular apoptosis in rats after ICH. **A** Rats underwent intracerebral injections of collagenase, and at 1 h, they received intracerebroventricular infusions of either normal saline or rhCDNF, and their neurological deficits were assessed on days 1, 3, and 7. **B** Representative coronal sections (2 mm thickness) show brain hemorrhagic areas of rats at 24 h and 72 h after ICH. **C** Lesion volume on days 1 and 3 (*n* = 8-9 each group) post-ICH was determined by morphometric measurements. **p < 0.01 Dunnett’s multiple-comparisons post hoc test, following two-way ANOVA [effect of treatment: *F*_(1,31)_ = 5.472, *P* = 0.0259]. **D** T2-weighted MRI images representative of the development of lesion size as outlined in red from days 1 to days 3 post-ICH. **E** Lesion volume measured from T2-weighted MRI images taken on days 1 and 3 post-ICH is expressed in relation to the size of the hemorrhagic lesion in the same animals on day 1. **p < 0.01. Student’s *t*-test was used for the analysis of statistical significance. **F** Diffused weighted image (DWI) sequences were scanned to assess hematoma volume visible in yellow regions and lesion volume as outlined in red at days 1 and 3 post-ICH induced by injection of collagenase. **G** Quantification of perihematomal edema in rats receiving rhCDNF or saline at days 1 and 3 post-ICH by subtracting hematoma volume from lesion volume *p < 0.05 Dunnett’s multiple-comparisons post hoc test, following two-way ANOVA [effect of treatment: *F*_(1,21)_ = 12.04, *P* = 0.0023] **H** Representative brain coronal sections (2 mm thickness) show Evans blue extravasation on day 3 post-ICH. **I** Comparison of dye concentrations in the ipsilateral and contralateral striatum between ICH + saline and ICH + rhCDNF groups. Dye concentration is expressed as μg/g of tissue weight and calculated from a standard curve obtained from known amounts of dye. *p < 0.05, Dunnett´s multiple-comparisons post-hoc test. Mean ± SEM is shown. Scale bars: 5 mm. **J** Modified neurological severity scores (mNSS) were examined before and 1 to 7 d post-ICH. *n* = 8 to 10 per group. ***p < 0.001 by Bonferroni’s multiple comparisons test, following two-way ANOVA [effect of treatment: *F*_(1,65)_ = 21.21, *P* < 0.0001]. **K** Forepaw use bias of the rats was assessed in the cylinder test before and 1 to 7 d after ICH. *n* = 7 per group. *p < 0.05; **p < 0.01, ***p < 0.001 by Bonferroni’s multiple comparisons test, following two-way ANOVA [effect of treatment: *F*_(1,48)_ = 29.76, *P* < 0.0001]. **L** Representative TUNEL staining images and **M** quantitative analysis of TUNEL-positive cells in each group 72 h after ICH. **p < 0.01 by Student’s *t* test. Mean ± SEM is shown. Scale bars: 20 µm.

To determine the effect of CDNF on ICH, rats underwent a single intracerebroventricular (i.c.v.) injection of either rhCDNF or saline vehicle 1 h after ICH induction by injection of collagenase or autologous blood (Fig. 2A). As a proof-of-principle, we first examined the distribution properties of exogenously administered rhCDNF. We administered Alexa Fluor® 488-labeled-rhCDNF i.c.v. 1 h post-ICH and validated that the labeled rhCDNF is limited to hCDNF-stained cells in the ipsilateral striatum (Fig. S2A, B). Six hours after i.c.v. injection of fluorescently labeled rhCDNF, the labeled rhCDNF co-localized with NeuN and GFAP and OX-42, but not RECA in the peri-hematoma area (Fig. S2C–F), indicating that rhCDNF injected in the lateral ventricle of brain is internalized by neurons, astrocytes, and microglia, rather than endothelial cells, respectively.

Next, we studied the gross distribution of hemorrhagic lesions by morphometric measurement 1 and 3 days after collagenase or autologous injection. Lesion areas manifested as oxygenated hematoma (dark red) at 1 day. In contrast, it would develop to lysed blood clot mixed with deoxygenated hemoglobin or methemoglobin (khaki grey) at 3 days after ICH (Fig. 2B). The lesioned area extended to its maximum at 1 day, maintained over 3 days, and regressed 7 days after ICH [30]. Thus, since rhCDNF has brain half-life of 5–6 h [31], we selected three dosages of rhCDNF and determined that delivery of rhCDNF at a dosage of 5 μg/2 μl would be sufficient for a therapeutic effect in a rat model of collagenase-induced ICH (Fig. S3A–C). While treatment with rhCDNF (5 μg/2 μl) did not affect lesion volumes at 1 day, it significantly reduced lesion volumes at 3 days after ICH induction by collagenase or autologous injection, compared with saline treatment (Fig. 2B, C; F (1, 31) = 8.574, *p* = 0.0063, two-way ANOVA Šidák multiple comparison test, Table 1b.1; Fig. S3D, E). The results of hemorrhagic lesion were validated by T2-weighted images of MRI from the same rats in each group at 1 and 3 days after ICH (Fig. 2D, E; *t*(6) = 5.482, *p* = 0.0015, paired *t*-test, Table 1b.2). Lesion volume changes on T2 images during hematoma resolution were significant in the CDNF-treated group compared to the saline-treated group. Similarly, spectrophotometric measurement showed that hemorrhagic volume on day 3 post-ICH was markedly reduced in the ICH + rhCDNF group, suggesting that CDNF accelerates hematoma clearance (Fig. S3F, G). By acquiring diffusion-weighted imaging (DWI) at 3 days after ICH, hemorrhagic brain lesions were observed as an area of hypointensity, caused by paramagnetic intracellular deoxyhemoglobin or methemoglobin, accompanied by a markedly hyperintense rim corresponding to extracellular water and brain edema (Fig. 2F). The volume of peri-hematoma edema was then calculated by subtracting the area of hypointensity from the whole lesion area in DWI at 3 days after ICH. Treatment with rhCDNF significantly decreased perihematomal edema by day 3, compared to saline treatment (Fig. 2G; F (1, 21) = 12.04, *p* = 0.0023, two-way ANOVA Šidák multiple comparison test, Table 1b.3). Since posttreatment with rhCDNF significantly reduces perihematomal edema after ICH, we next investigated whether rhCDNF treatment affects vascular permeability of the blood-brain barrier (BBB). On day 3 after ICH, Evans blue concentrations in the hemorrhagic striatum in the ICH + rhCDNF group were smaller than in the ICH + saline group (Fig. 2H, I; *F* (1, 14) = 9.895 *p* = 0.0072, two-way ANOVA Šidák multiple comparison test, Table 1b.4), indicating that posttreatment with rhCDNF significantly reduced ICH-induced BBB leakage. Dye concentrations in contralateral striatum were not different between the ICH + saline and ICH + rhCDNF groups (Fig. 2I; t(14) = 1.252, *p* = 0.41, paired *t*-test), suggesting that unilateral striatal ICH in the present experimental regime appeared not to affect the BBB of the contralateral striatum.

Neurological function was evaluated using modified Neurological Severity Score (mNSS) and cylinder test at days 1, 3, and 7 post-ICH. Compared with the control group of saline-infused rats, rats infused with rhCDNF had a significantly faster reversal of neurological deficits at both time points (Fig. 2J). Additionally, rhCDNF-injected rats exhibited milder injury-induced behavioral deficits in the paired or right forepaw use of cylinder tests (Fig. 2K; F (1, 48) = 29.76, *p* < 0.0001, two-way ANOVA Bonferroni’s multiple comparison test, Table 1b.6). Given that rhCDNF treatment mitigates ICH-induced behavioral deficits, we investigated whether rhCDNF treatment affects the number of apoptotic cells around the hematoma core. Our recently published data demonstrate that ICH increases apoptotic cells, namely TUNEL-positive cells, and apoptosis occurs in neurons, endothelium, and microglia [32]. Compared with saline treatment, rhCDNF treatment decreased the number of TUNEL-positive cells at day 3 (Fig. 2L, M; *t*(16) = 2.925, *p* = 0.0099, paired *t*-test, Table 1b.7). Collectively, these findings demonstrate that rhCDNF treatment accelerates hemorrhagic lesion resolution, ameliorates hematoma-induced brain edema, decreases cell death, and promotes recovery of neurological functions.

### CDNF upregulates the expression of scavenger receptors on microglia/macrophages in the hemorrhagic striatum

**Fig. 3:**
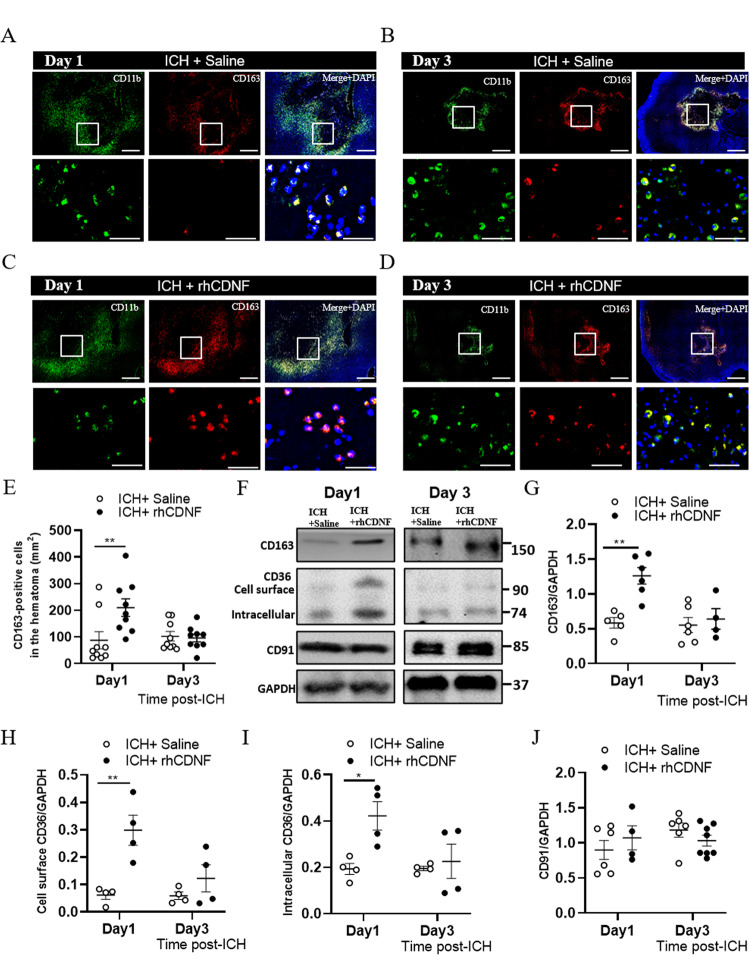
CDNF treatment enhanced expression of scavenger receptors on activated microglia/macrophages at the early stage of ICH. **A**–**D** Figures show immunofluorescent staining of cells in hemorrhagic striatum with anti-CD11b (green), anti-CD163 (red) and counterstained with DAPI (blue). The insets show the hematoma edge in higher-magnification photomicrographs immunostained with CD11b and CD163 antibodies. Immunofluorescence pictures showing co-localization of CD163 and CD11b in the saline-treated perihematomal area at 1 d (**A**) and 3 d (**B**), respectively. **C**–**E** rhCDNF injections increased CD163 immunostaining in the peri-hematoma area at 1 d, but not at 3 d post-ICH, compared with saline group. **p < 0.01 Bonferroni’s multiple comparisons test, following two-way ANOVA [effect of treatment: *F*(1,32) = 5.026, *P* = 0.03]. **F**–**J** WB bands of CD163, CD36, CD91, and GAPDH at different time points post-ICH. **G** Increased the level of CD163 in the rhCDNF-treated hemorrhagic striatum at 1 d but not at 3 d post-ICH. **P < 0.001, Bonferroni’s multiple comparisons test, following two-way ANOVA [effect of treatment: *F*(1,12) = 44.52, *P* < 0.001]. **H**, **I** Increased levels of cell surface CD36 and intracellular CD36 were detected in rhCDNF-treated hemorrhagic striatum at 1 d post-ICH. *p < 0.05, **p < 0.01, Bonferroni’s multiple comparisons test, following two-way ANOVA [effect of treatment: *F*(1,18) = 30.2, *P* < 0.001. **J** rhCDNF treatment did not increase the level of CD91 in the hemorrhagic striatum, compared to a saline-treated group post-ICH. The mean ± SEM of three independent experiments is shown. Scale bars: **A**, **B**: 1000 µl, **C**, **D**: 50 µl.

Since rhCDNF treatment accelerates resolution of blood components and mitigates hematoma-induced secondary brain injury, we next asked whether post-ICH delivery of rhCDNF induces a distinct phenotype of immune cells that can remove extravasated erythrocytes or offset toxic components of the lysed hematoma. Due to the significant difference in the hematoma volume between saline and rhCDNF-treated groups, we hypothesized that this result could be attributed to difference in removal of blot clots by phagocytosis. Therefore, we quantitatively analyzed the spatiotemporal expression of scavenger receptors in microglia/macrophages. We co-stained CD11b, a microglia/macrophage marker, with three scavenger receptors, i.e. CD163, CD36, and CD91, in the hemorrhagic striatum using immunohistochemistry. Immunohistochemical analysis of brains from rats sacrificed 1 and 3 days after collagenase injection, revealed numerous CD11b^+^ cells in the peri-hematoma region at day 1 post-ICH. CD11b^+^ cells were still found at day 3 post-ICH though hematoma volume was partially resolved (Fig. 3A–D and Fig. S4). CD163 is a hemoglobin scavenger receptor, exclusive to the brain’s microglia/hematogenous macrophages mediating endocytosis of hemoglobin:haptoglobin (Hb:Hp) complexes and thereby counteracting Hb-induced oxidative tissue damage [33]. We found that the number of CD11b^+^CD163^+^ cells in the hemorrhagic striatum was increased in the rhCDNF-treated group compared to the saline-treated group at 24 h after ICH (Fig. 3A, C, E; *F* (1, 32) = 5.026, *p* = 0.03, two-way ANOVA Bonferroni’s multiple comparison test, Table 1c.1). However, we did not detect any difference in the number of CD11b^+^CD163^+^ cells between the two groups at 72 h after ICH (Fig. 3B, D, E).

CD36 and CD91, scavenger receptors expressed on microglia/macrophages, vascular endothelial cells, and astrocytes, were highly expressed in CD11b^+^ cells at day 1 post-ICH (Fig. S4A, C, E, G; *F* (1, 17) = 10.95, *p* = 0.004, two-way ANOVA Šidák multiple comparison test, Table 1c.2). At day 3 post-ICH, CD36 was mostly colocalized with CD11b in the perihematomal tissue (Fig. S4F). On the other hand, CD91 had a wider distribution in the ipsilateral subcortical region, not only colocalized with CD11b (Fig. S4B, D, F, H). By quantitative immunoblotting analysis, levels of CD163, cell surface/intracellular CD36, but not CD91 in the hemorrhagic lesion significantly increased in the rhCDNF-treated group at 24 h post-ICH compared with the saline-treated group (Fig. 3F–J; cell surface CD36: *F* (1, 12) = 15.23, *p* = 0.002; intracellular CD36: F (1, 12) = 6.799, *p* = 0.02; CD91: *F* (1, 20) = 1.099, *p* = 0.93, two-way ANOVA Bonferroni’s multiple comparison test, Table 1c.3–5). At 72 h post-ICH, there was no difference in the level of CD163, CD36, or CD91 between groups. Overall, these results suggest that rhCDNF treatment increases expression of scavenger receptors, CD163 and CD36 mainly in microglia/macrophages, at early stage of ICH. Increases scavenger function shown in these cells might contribute to enhancing hematoma clearance observed in animals treated with rhCDNF.

### CDNF mitigates the UPR, oxidative stress, and GSK-3β activity but increases Nrf2—mediated HO-1 expression

**Fig. 4:**
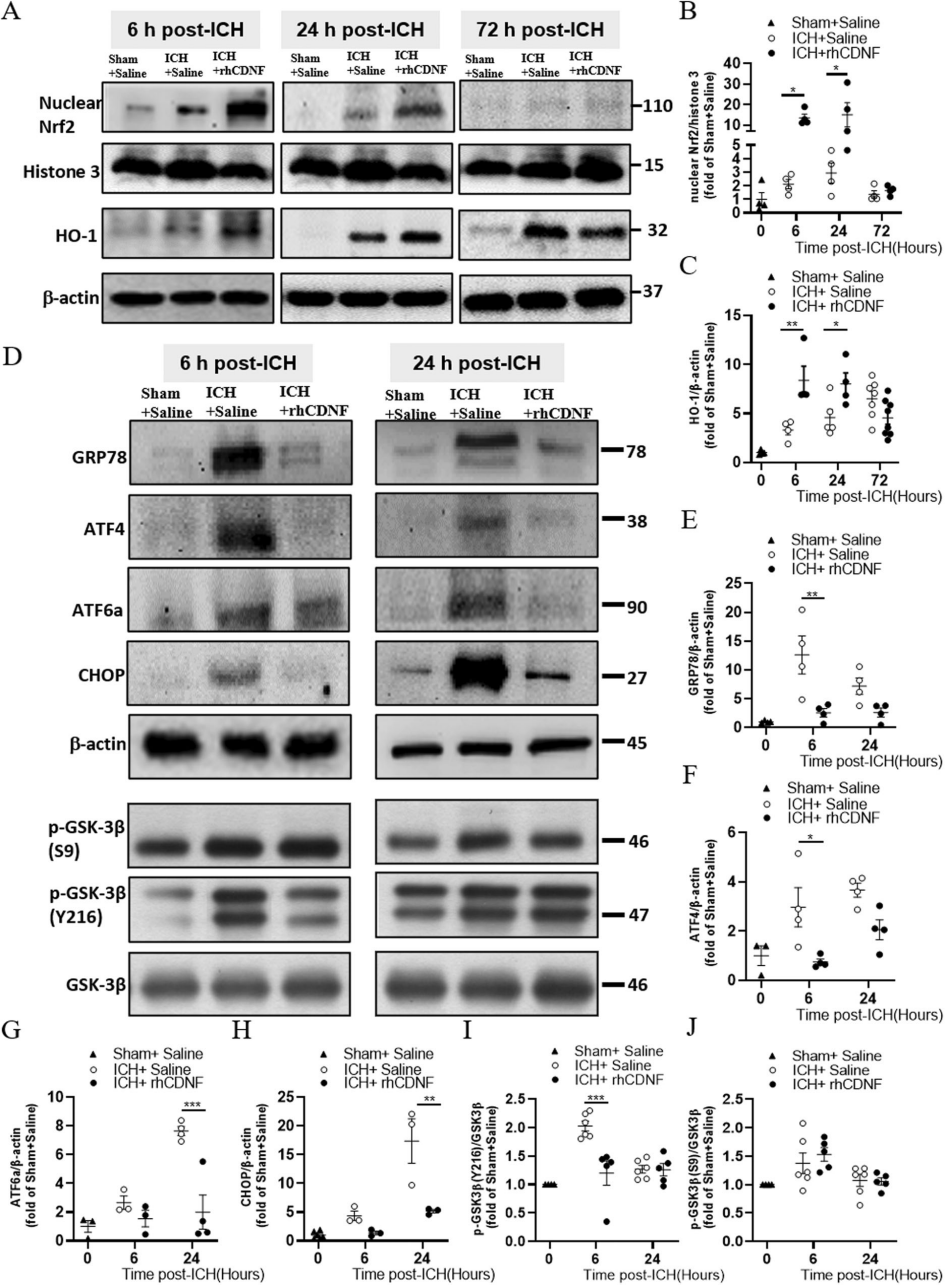
CDNF mediated Nrf2/HO-1 axis activation is implicated in decreasing UPR marker expression and tempering GSK3β activation. **A** Representative Western blot images of nuclear Nrf2, Histone 3, Ho-1, GAPDH in the striatum and the area of hemorrhagic lesion at different time points. **B, C** The protein expression levels of Ho-1 and Nrf2 in the nucleus were detected in the striatum of indicated groups of rats receiving sham + saline, ICH + saline, or ICH + rhCDNF. Increased levels of nuclear Nrf2 and Ho-1 were shown in the rhCDNF-treated group at 6 h and 24 h post-ICH. **D** Representative Western blot from tissue lysates of intact or hemorrhagic striatum probed with antibodies to UPR markers, p-GSK-3β (S9), p-GSK-3β (Y216), and GSK-3β. Β-actin was used as an internal marker. **E**–**J** Data points show protein expression levels of GRP78, Atf4, Atf6, and Chop in the naïve or hemorrhagic striatum of indicated groups of rats at 6 h and 24 h post-ICH. Expression of p-GSK-3β (S9) **(I)**, and p-GSK-3β (Y216) **(J)** were analyzed by immunoblotting quantified by densitometry and normalized to expression of GSK-3β. Results are presented as fold change increase compared to sham plus saline control. Mean± SEM, *n* = 3–4/sham group, *n* = 4–8/ group of ICH plus saline or ICH plus rhCDNF. Two-way ANOVA + post-hoc Bonferroni test, *p < 0.05; **p < 0.01; ***p < 0.0001 in comparison to the ICH + saline group.

Although CDNF was shown to possess an anti-inflammatory capacity in previous studies, the precise operating mechanism by which it affects innate immune cells remains unclear. Thus, we hypothesized that rhCDNF coordinates intracellular signals to upregulate the expression of scavenger receptors on microglia/macrophages after ICH. It is well documented that Nrf2 in microglia/macrophages plays an essential role in regulating the phagocytic function for hematoma clearance in experimental ICH [34]. At 6 and 24 h post-ICH, the basal nuclear Nrf2 level was increased in the area of the hemorrhagic lesion where numerous CD11b^+^ microglia/macrophages accumulate (Fig. 4A), suggesting that cells are exposed to excessive oxidative stimuli from hemorrhagic injuries. Notably, rhCDNF treatment caused a further increase in the nuclear Nrf2 level compared to the saline-treated group at 6- and 24-h post-ICH (Fig. 4A, B; *F* (1, 18) = 14.37, p = 0.001, two-way ANOVA Bonferroni’s multiple comparison test, Table 1d.1). As expected, HO-1 protein content was higher in the hemorrhagic area of the rhCDNF-treated group at the corresponding time points (Fig. 4A, C; *F*(1, 26) = 8.826, *p* = 0.006, two-way ANOVA Bonferroni’s multiple comparison test, Table 1d.2). However, at 72 h post-ICH (Fig. 4C), there was a marginal decrease in the level of HO-1 in the rhCDNF-treated group, suggesting that treatment with rhCDNF increases transcriptional activity of Nrf2 and its antioxidant response element HO-1 in a time-dependent manner. Next, we investigated the possible involvement of the downstream regulatory mechanism of CDNF, which has previously been shown to correlate with Nrf2 activation.

According to immunoblotting analysis of rats sacrificed at 6 or 24 h post-ICH, there were no treatment-induced changes in phosphorylation of Akt, ERK, p38, and JNK in the lesioned area (Fig. S5A–E). Then, we sought to identify whether CDNF coordinates UPR and related intracellular signals in this area. Interestingly, at 6 h post-ICH, we only found an upregulation of GRP78 and ATF4 (Fig. 4D–F; GRP78: *F* (1, 12) = 15.28, *p* = 0.002; ATF4: *F* (1, 12) = 16.37, *p* = 0.002, two-way ANOVA Bonferroni’s multiple comparison test, Table 1d.3, 1d.4), a downstream protein of the PERK pathway, which blocks general protein translation initiation in stressed cells. Meanwhile, activation of PERK-ATF4 pathway has been reported to phosphorylate and activate Nrf2 that contributes to the adaptive UPR-related protective response during ER stress [35]. At 24 h post-ICH, the ATF6 pathway was upregulated, in particular levels of CHOP protein, which has a prominent role in ER-stress induced apoptosis, increased more than 4-fold (Fig. 4D, G, H; ATF6: *F* (1, 10) = 18.24, *p* = 0.002; CHOP: F (1, 8) = 14.58, *p* = 0.005, two-way ANOVA Bonferroni’s multiple comparison test, Table 1d.5, 1d.6). Notably, upon rhCDNF delivery, GRP78, ATF4, ATF6α, and CHOP proteins decreased time-dependently to sham levels (Fig. 4D–H), implying that rhCDNF treatment attenuated these markers of different UPR branches in the hemorrhagic area of the striatum. Moreover, protein carbonyl levels, an indicator of oxidative stress, were less elevated in the rhCDNF-treated group than in the saline-treated group (Fig. S5F and G), suggesting that rhCDNF treatment could attenuate ICH-induced oxidative stress. In previous studies, an increased level of pY126-GSK3β corresponding to decreased pS9-GSK-3β has been shown to be associated with elevated levels of ER stress or oxidative stress [36]. Interestingly, we only found increased expression of pY126-GSK3β at 6 h post-ICH, implying that catalytic activity of GSK3β increases in the early stage of ICH (Fig. 4D, I, J: p-GSK3β (Y216): *F* (1, 18) = 11.41, *p* = 0.003; p-GSK3β (S9): *F* (1, 18) = 0.275, *p* = 0.61, two-way ANOVA Bonferroni’s multiple comparison test, Table 1d.7, 1d.8). By mitigating ER and oxidative stress, rhCDNF was also shown to decrease active Tyr216 phosphorylation of GSK3β at Tyr216 (Fig. 4D, I), an upstream molecule regulating nuclear translocation of Nrf2. These findings suggest that rhCDNF treatment could modulate the PERK signaling pathway accompanied by decreasing CHOP expression, blocking GSK3β activation, and further upregulating Nrf2 transcriptional activity. This may contribute to CDNF’s cytoprotective effect and hematoma-scavenging ability.

### CDNF regulates inflammatory responses primarily by suppressing pro-inflammatory cytokines but promoting anti-inflammatory mediators after hemorrhagic stroke

To get a broader view of the imbalance of immune cell function in response to ICH and of how rhCDNF treatment could modulate immune responses via its ability to increase expression of scavenger receptors on microglia/macrophages, we characterized the time course of cytokine responses in the striatum surrounding hematoma area. We then tested the efficacy of post-ICH rhCDNF treatment in adult rats. Significant increases in TNF-α, IFN-γ, IL-1β, and IL-6 levels were observed 1 and 3 days post-ICH within the peri-hematoma striatum, where IFN-γ remained elevated at 7 days post-ICH (Fig. S6A–D). Meanwhile, IL-1β and IL-6 returned to basal levels, and the level of TNF-α in the peri-hematoma region was even lower than that in the control group at 7 days post-ICH, indicating a reaction to the continued immune response. Similarly, down-regulation of IL-10 was observed 1 day post-ICH in the peri-hematoma striatum, where it remained decreased until 7 days (fig. S6E). Administration of rhCDNF resulted in a significant decrease in TNF-α, IFN-γ, IL-1β, and IL-6 levels, but an increase in IL-10 level within the peri-hematoma striatum at 1 and 3 days post-ICH (Fig. S6A–E). Interestingly, rhCDNF treatment preserved upregulation of IL-10 and restored TNF-α to normal levels at day 7. Collectively, these findings indicate that rhCDNF injections have long-term immunomodulatory effects comprising attenuation of pro-inflammatory cytokines accompanied by an increase in anti-inflammatory mediator after ICH insult.

### CDNF reduces the number of CD11b^+^ cells but enhances the erythrophagocytic process at three days post-ICH

**Fig. 5:**
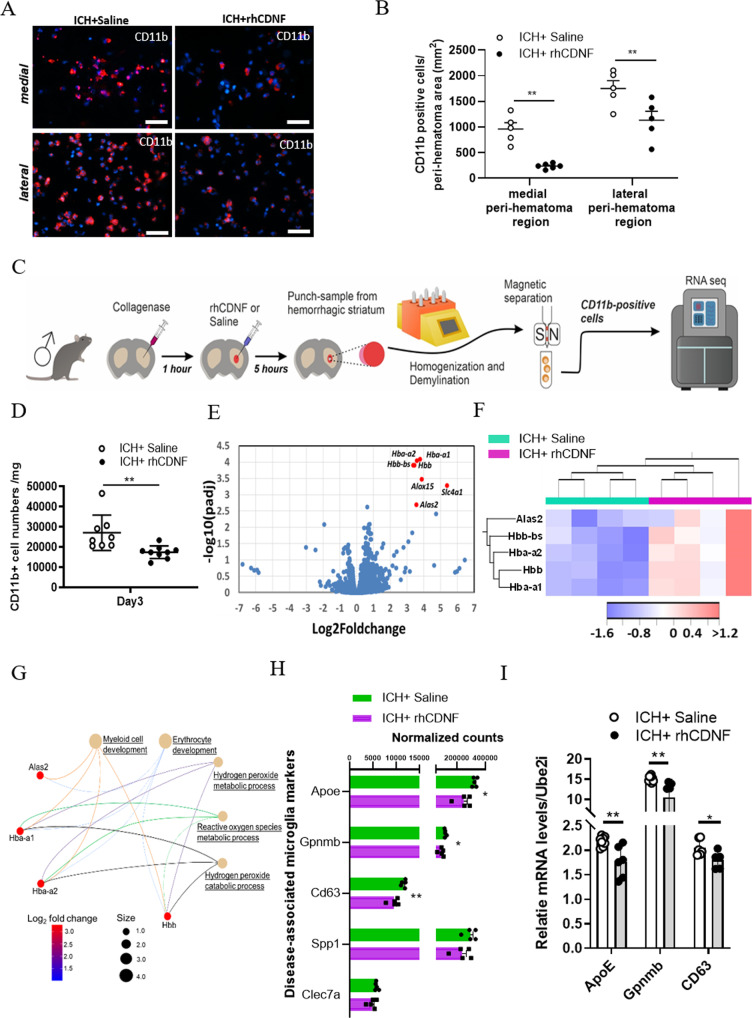
Post-ICH treatment with rhCDNF reduces the number of CD11b^+^ microglia/macrophages but enhances its erythrophagocytosis on day 3. **A** Immunohistochemical staining of CD11b expression at 3 days post-ICH reveals the distribution of CD11b^+^ cells in the medial and lateral peri-hematoma striatum of indicated groups. **B** Statistical measurement of striatal CD11b^+^ cells subjected to ICH injury in the ICH + Saline and ICH + rhCDNF groups, respectively. **p < 0.01 by Student’s *t* test. *N* = 5/each group. **C** Microglia**/**macrophages were purified from hemorrhagic striatum at day 3 post-ICH using gentleMACs Dissociator and CD11b antibody-beads. The gene profile of CD11b^+^ cells was analyzed by RNA sequencing. **D** The number of isolated CD11b^+^ cells were reduced in the hemorrhagic striatum of animals receiving intraventricular injections of rhCDNF, comparedi with saline. **p < 0.01 by Student’s *t* test. **E** Volcano plot comparing log2fold changes and adjusted *p*-values of 22250 gene expressions. The red dots indicate genes biologically upregulated (log2fold change >2, adjusted p-value <0.01). The blue dots indicate genes with no significant change between two groups. Note: only validated genes are included in this list. **F** Heatmap plot of DEGs in CD11b^+^ myeloid cells in the area of hemorrhagic striatum between ICH + saline and ICH + rhCDNF. **G** Molecular network plot connected using GO overrepresentation analysis from CD11b^+^ cells following rhCDNF treatment in a rat model of ICH. **H** CD11b^+^ cells from rhCDNF-treated group express low transcript number of disease-associated microglial (DAM) genes. **I** qPCR analysis of gene expression of DAM (ApoE, Gpnmb and CD63) in the CD11b-expressing microglia/macrophages of ICH + saline and ICH + rhCDNF groups. *n* = 5. *P < 0.05; **P < 0.01, Multiple-Mann–Whitney tests. Scale bars: 20 µL.

Immunostaining analysis revealed that 3 days post-ICH, there were fewer CD11b-positive microglia/macrophages in the striatum where the hematoma was partially resolved (Fig. 3B, D). Thus, we asked whether CDNF, promoting the resolution of hemorrhagic lesions, would further decrease the number of CD11b^+^ cells or direct them toward certain phenotypes. Indeed, we found that at 3 days post-rhCDNF administration, there were fewer CD11b^+^ cells in the medial or lateral peri-hematoma region of the striatum (Fig. 5A, B; F (1, 17)=29.03, *p* < 0.001, two-way ANOVA Bonferroni’s multiple comparison test, Table 1e.1). In our previous study, MANF, a paralogous protein of CDNF, was shown to increase the density of ARG1^+^ and CD68^+^ cells in subcortical regions after stroke [24]. In hemorrhagic stroke, rhCDNF treatment did not increase the density of ARG1-positive cells, but rather modestly decreased the density of CD68-positive cells in the lateral peri-hematoma striatum (Fig. S7A–D). These findings indicate that rhCDNF treatment decreases activation of microglia/macrophages but does not direct immune cells toward alternative activation at the subacute stage of ICH.

To compare the transcriptomic profile of immune cells from the hemorrhagic striatum of saline-treated and rhCDNF-treated rats, we performed RNA-sequencing (Fig. 5C). The hemorrhagic striatum was dissected, and single immune cells were isolated by gentleMACS columns using CD11b^+^ selection. In line with results obtained from immunofluorescence, rhCDNF treatment significantly reduced the number of CD11b^+^ cells on day 3 post-ICH (Fig. 5D; *t*(15) = 3.096, *p* = 0.0074, paired *t*-test, Table 1e.2). Isolated cells scantly expressed markers for neurons (*Elavl3*, *Map1b*, and *Map2*), astrocytes (*Aldh1l2*, *Slc1a2*, and *Gja1*), oligodendrocytes (*Mag*, *Mog*, and *Olig1*), and endothelial cells (*Cdh5* and *vWF*), confirming that our sorted populations were highly enriched for myeloid cells (*Itgax*, *Trem2* and *Cx3cr1*), without substantial cross-contamination (Fig. S8A). In addition, these myeloid cells not only expressed microglial markers highly (*P2ry12*, *Slc2a5*, *Tmem119,* and *Gpr34*), but also peripheral monocyte/macrophage signature genes (*F10*, *Emilin2*, *F5*, *Gda*, and *Mki67*), suggesting that isolated CD11b^+^ cells are enriched in microglia and peripheral monocytes/macrophages (Fig. S8A). The volcano plot in Fig. 5E illustrates the results of a different gene analysis of CD11b^+^ cells from two groups (*n* = 5, each group). Genes were identified as differentially expressed genes (DEGs) only when the fold difference between two groups was greater than 3 and the adjusted p values were lower than 0.01. Among the most overexpressed genes in CD11b^+^ striatal microglia/macrophages from rhCDNF-treated rats were marker genes of the erythrophagocytic process (*Hba-a1, Hba-a2, Hbb, Hbb-bs,* and *Alas2*) (Fig. 5E). Accordingly, network-based gene ontology (GO) enrichment analysis of DEGs identified myeloid cell development, erythrocyte development, hydrogen peroxide metabolic process, reactive oxygen species metabolic process, and hydrogen peroxide catabolic process as the most upregulated processes (Fig. 5F). Of note, mRNA copy numbers of *ApoE, Gpnmb*, and *CD63* genes which are upregulated in disease-associated-microglia (DAM) [37], were lower in CD11b^+^ cells from the rhCDNF-treated group (Fig. 5H). qPCR analysis verified that the expression of *ApoE*, *Gpnmb*, and *CD63* were downregulated in the rhCDNF-treated group compared with saline-treated (Fig. 5I; ApoE: t(12) = 3.51, p = 0.004; Gpnmb: *t*(12) = 2.87, *p* = 0.013; CD63: *t*(9) = 2.47, *p* = 0.037, Multiple unpaired *t*-test, Table 1e.3). These differential expressions of transcripts associated with the genes of the erythrophagocytosis recapitulate that CDNF boosts the hematoma-scavenging function in activated microglia/macrophages.

### CDNF promotes erythrocytes phagocytosis and expression of scavenger receptors on BV2 microglial cells

**Fig. 6:**
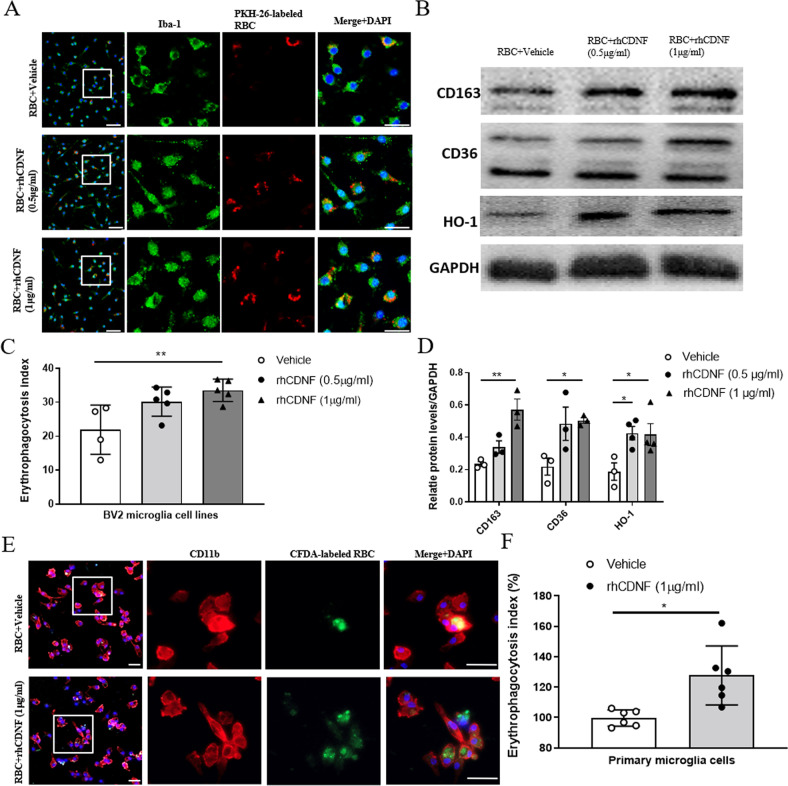
CDNF directly increases RBC engulfment by microglia and upregulates scavenger receptor expression on BV2 cell lines. **A** Representative immunofluorescence images show engulfment of PKH-26-labeled erythrocytes (red) in microglia (Iba-1; green) after erythrocyte incubation for 6 h. BV2 cells were incubated with saline or rhCDNF for 1 h before treatment with erythrocytes. Scale bar: 100 μm. **B** WB bands of CD163, CD36, and HO-1 in BV2 cell lines after incubation with erythrocytes and different concentrations of rhCDNF treatment for 6 h. **C** Quantification of erythrophagocytosis. *n* = 3 independent experiments/group; each experiment includes 3 technical replicates. **p < 0.01 vs. RBC + vehicle. Data were analyzed as repeated measures by one way ANOVA followed by Bonferroni correction. **D** Quantitative analysis of CD163, CD36, and HO-1 expression in BV2 cell lines treated with rhCDNF (0.5 or 1 μg/ml) for 1 h before incubation with erythrocytes. *P < 0.05, **P < 0.01 by one-way ANOVA followed by Bonferroni corrections. Mean ± SEM is shown. **E** A photomicrograph of CD11b-immunolabled microglia in cultures at 6 h after exposure to CFDA-labeled RBCs in the presence of 1 μg/ml rhCDNF or PBS. **F** Phagocytosis was measured by measuring the fluorescence intensity in the microglial lysate at 6 h after exposure to RBCs. RhCDNF at 1 μg/ml significantly augmented phagocytosis, when compared to PBS treatment. Data were analyzed as two-tailed Student’s *t*-test. *P < 0.05 (*n* = 5).

Given that phagocytosis is required to eliminate the hematoma after intracerebral hemorrhage, we next want to investigate if CDNF can directly manipulate the phagocytic activity of microglia/macrophages under pathological condition. To examine the effect of rhCDNF treatment on microglial phagocytosis in response to RBC stimulation, BV2 cells were treated with rhCDNF for 1 h and then incubated with PKH-labeled erythrocytes for 6 h to investigate the erythrophagocytosis. We found that there is a marginal increase in engulfment of erythrocytes after rhCDNF 0.5 μg/ml treatment (Fig. 6A, C; *F* (2, 11) = 6.222, *p* = 0.0156, one-way ANOVA Dunnett’s multiple comparison test, Table 1f.1). However, a 1.5-fold elevation of phagocytosis was observed after 6 h in the 1 μg/ml rhCDNF concentration (Fig. 6A, C), suggesting that CDNF directly promotes erythrocyte engulfment in BV2 cell lines. In line with in vivo results, CD36 and CD163 expression were increased by 2-fold and by 2.5-fold, respectively after rhCDNF treatment (1 μg/ml; 6 h) (Fig. 6B, D; CD163: *F* (2, 6) = 15.16, *p* = 0.0045; CD36: *F* (2, 6) = 5.56, *p* = 0.043; HO-1: *F* (2, 8)=5.085, *p* = 0.0376, one-way ANOVA Dunnett’s multiple comparison test, Table 1f.2). Furthermore, expression of HO-1 was increased in rhCDNF-treated BV2 cells after RBC incubation for 6 h (Fig. 6B, D). As BV2 cell line does not exactly exhibit the real function of microglia, we adopted a primary microglial culture system and used isolated RBCs as indicators of phagocytosis. Based on previous studies, the phagocytosed RBCs in microglia were shown at 6 h. To quantify phagocytosis, we loaded RBCs with fluorescence dye (CFDA) and then exposed them to microglia (Fig. 6E). The fluorescence intensity in the cell lysates was measured. The erythrocyte phagocytosis was significantly increased by treating microglia with rhCDNF, demonstrated by 27.7% more fluorescence within microglia (phagocytosis index) after microglia exposed to RBCs for 6 h (Fig. 6F; *t*(5) = 2.972; *p* = 0.031, paired *t*-test, Table 1f.3). Overall, these findings demonstrated CDNF treatment can directly increase microglia scavenger receptors, CD36, CD163 expressions and its erythrophagocytosis.

### Intravenous delivery of rhCDNF improves ICH outcomes

**Fig. 7:**
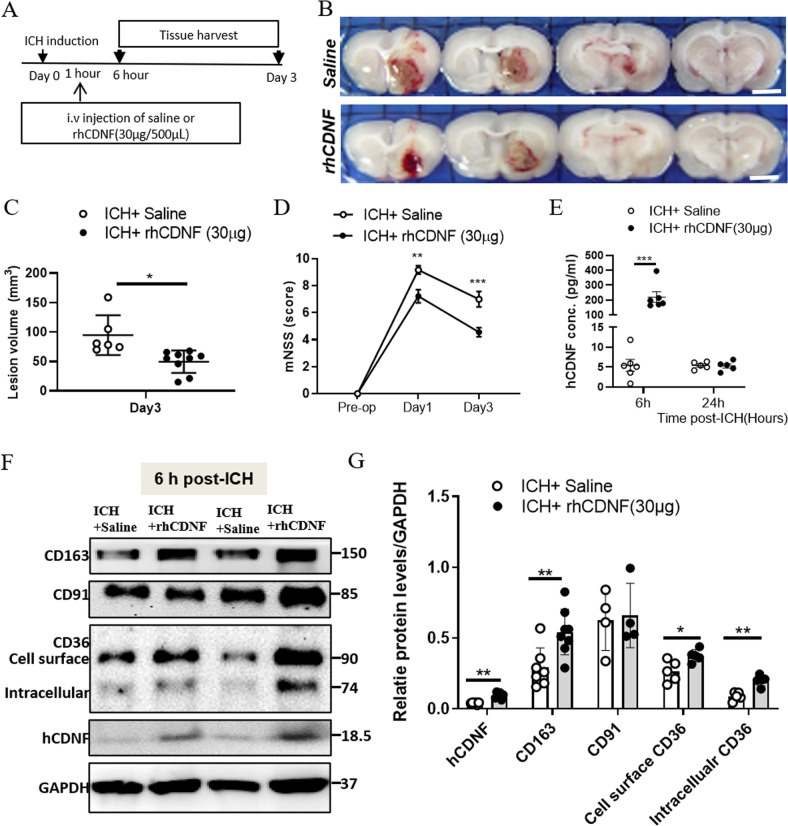
Effects of systemic treatment with rhCDNF on lesion volume, neurobehavioral function, and the expression of scavenger receptors in the hemorrhagic striatum. **A** Timeline of treatment. **B** Coronal sections of the brain exhibit lesion volume with intravenous injection of saline, and rhCDNF, respectively at day 3 post-ICH. **C** Lesion volume on days 3 (*n* = 6–8, each group) post-ICH was determined by morphometric measurement. *p < 0.05 by Student’s *t* test. **D** Modified neurological severity scores (mNSS) were examined before and 1 to 3 d after ICH. **p < 0.01, ***p < 0.001 by Bonferroni’s multiple comparisons test, following two-way ANOVA [effect of treatment: *F*(1,35) = 18.93, *P* = 0.0001]. **E** Human CDNF concentration in hemorrhagic stroked rat serum 6 h and 24 h after intravenous administration of 30 μg rhCDNF (*n* = 5-6) or saline (*n* = 5). Two-way ANOVA + post-hoc Bonferroni test, ***p < 0.0001 in comparison to the ICH + saline group. **F** WB bands of CD163, CD36, CD91, hCDNF and GAPDH at 6 h post-ICH. **G** Quantitative analysis of hCDNF, CD163, CD91, and intracellular/cell surface CD36 expression in the area of hemorrhagic lesion. *<0.05, **P < 0.01 by multiple comparisons using the Holm-Šidák method. Mean ± SEM is shown. Scale bars: 5 mm.

Based on the above results of immune modulation by CDNF and considering safety and convenience of systemic administration for clinical application, we hypothesized that CDNF can also regulate the function of microglia/macrophages with systemic administration. We subjected rats to ICH and then treated them with intravenous (i.v.) injection of rhCDNF. Systemic administration of rhCDNF reduced lesion volume at day 3 and attenuated neurological deficits, demonstrated by mNSS (Fig. 7A–D; lesion volume: *t*(5) = 2.707, *p* = 0.0424, paired *t*-test, Table 1g.1; mNSS: *F* (1, 35) = 18.93, *p* < 0.001, two-way ANOVA Bonferroni’s multiple comparison test, Table 1g.2). Given that systemic treatment of rhCDNF may directly affect physiological parameters to mitigate ICH-induced brain injury, an additional 20 rats were randomly assigned to determine the duration of rhCDNF bioavailability and then evaluate the physiological parameters in this duration. Approximately 0.67% of the dose remained in the blood circulation at 6 h post-i.v. administration, but human rhCDNF was nearly undetectable at 24 h post-i.v. administration (Fig. 7E; *F* (1, 18) = 29.41, *p* < 0.001, two-way ANOVA Bonferroni’s multiple comparison test, Table 1g.3). Moreover, we found rhCDNF is transported from the systemic circulation into the peri-hematoma striatum 6 h post-ICH (Fig. 7F, G). Therefore, we decided to investigate the physiological parameters before ICH induction and at different time points after i.v. injections of saline or rhCDNF. There were no significant differences in mean arterial pressure (MABP), heart rate (HR), PaO2, sodium (Na), potassium (K), glucose, lactate, and hemoglobin (Hgb) for the time points studied between the ICH + saline and ICH + rhCDNF groups (Fig. S9A–H), indicating that exogenous rhCDNF in the circulation did not induce significant changes in hemodynamics or blood chemistry. Finally, to verify whether this rhCDNF-mediated benefit is also attributable to the hematoma-scavenging function of microglia/macrophages, we analyzed the markers of scavenger receptors in the peri-hematoma area at 6 h post-i.v. rhCDNF injection. Indeed, the expression of CD163 and cell surface/intracellular CD36 were up-regulated, whereas the increase in CD91 expression was statistically significant (Fig. 7F, G; hCDNF: *t*(10) = 5.47, *p* < 0.0001; CD163: *t*(13) = 3.2, *p* = 0.007; CD91: *t*(6) = 0.217, *p* = 0.084; Cell surface CD36: *t*(8) = 2.77, *p* = 0.02; Intracellular CD36: *t*(8) = 5.18, *p* < 0.001, Multiple unpaired *t*-test with Holm-Šidák multiple comparison test, Table 1g.4). The differential expression of proteins associated with hematoma-scavenging functions implies that systemic delivery of rhCDNF facilitates myeloid cells acquiring a significant subset of innate pattern recognition receptors that are crucial in hematoma-scavenging capacity and the maintenance of cerebral homeostasis.

## Discussion

Spontaneous ICH is a life-threatening stroke subtype that lacks effective drug therapy. Although the pathogenesis of primary brain injury results from the mechanical effect of the hematoma, release of toxic substances due to erythrocyte lysis is a crucial factor leading to ER stress which when combined with oxidative stress and imbalance in immune cell function precipitates secondary brain injury [38]. Therefore, redox signaling and immune cell activation become interesting targets for therapeutic intervention by coordinating the interplay of UPR [28]. Here, we first demonstrate that endogenous levels of CDNF change during disease progression, decreasing at hematoma accumulation stages and increasing at a later period of hematoma resolution. In particular, genetic ablation of CDNF markedly impaired hemorrhagic lesion resolution and downregulated Hmox1 transcription at the phase of hematoma accumulation. Hmox1 gene codes for the enzyme HO-1, which exerts potent antioxidant and anti-inflammatory effects by promoting heme catabolism to produce CO and bilirubin [39]. Previous studies have demonstrated that HO-1 is rapidly induced after ICH and primarily expressed in activated microglia in the early stages post-ICH [40]. Although the effects of HO-1 expression on microglia/macrophages following ICH remain controversial, appropriately upregulating HO-1 expression associated with regulation of Nrf2 entry into the nucleus may play a neuroprotective role in the early stage of ICH [41].

Further, immunofluorescence staining revealed that most HO-1-positive cells expressed CD68 and CD163, indicating that HO-1 expression may define a subtype of activated microglia/macrophages with a specific role in anti-inflammation processes following brain injury [42]. Furthermore, Schallner et al. reported that microglial HO-1 is necessary to attenuate neuronal cell death, vasospasm, impairments in cognition function, and clearance of the cerebral hematoma [43]. Based on the results described here, we argue that endogenous CDNF might preserve upregulated HO-1 expression on microglia/macrophages at the early stage of ICH. In addition, the deficiency in endogenous CDNF affects hematoma resolution, suggesting that the administration of exogenous protein will adopt this mechanism to have a therapeutic effect after ICH.

Current scientific research suggests that augmenting endogenous clearance pathways for red blood cells and promoting microglia/macrophage-mediated phagocytosis as a potential therapeutic strategy for ICH [44]. Notably, scavenger receptors, expressed on microglia/macrophages, mainly function in endocytosis of exogenous invaders [34]. This study observed that the number of CD11b^+^ cells increased in the perihematomal region at 24 h post-ICH. The recruitment of activated microglia and macrophages may explain the increased number of CD11b^+^ cells post-ICH, consistent with previous studies showing that phagocytic microglia/macrophages increased in the peri-hemorrhagic areas [45]. However, the anti-CD11b antibody cannot specifically identify phagocytic microglia/macrophages post-ICH. Here, we demonstrate for the first time that CDNF can augment the expression of CD36 and CD163 in the hemorrhagic area at the early stage of ICH. Nrf2 activation upregulates CD36 expression, a type II scavenger receptor that plays an important role in mediating recognition and phagocytosis[8]. CD163, a hemoglobin scavenger receptor, is expressed in M2-like microglia/macrophages and mediates IL-10 release and the Nrf2/HO-1 pathway [46]. In this study, CDNF treatment upregulated the hematoma-scavenging activity of activated microglia/macrophages in a time-dependent manner, implying that CDNF can modulate microglia/macrophage phagocytosis and fuel anti-oxidative stress and anti-inflammatory responses to alleviate secondary brain injury. This effect of CDNF on myeloid cell function is similar to that of its analog protein, MANF, in ischemic stroke, unselectively enrolling phagocytic macrophages [24].

Given that Nrf2 plays a crucial role in regulating the phagocytic functions of microglia/macrophages [47], we propose that CDNF exploits specific molecular mechanisms to induce Nrf2 activation. Distinct from orchestrated mechanisms of regulating Nrf2 activation, CDNF did not mediate activation of the Akt, ERK1/2, p38, or JNK pathways. In contrast, CDNF can modulate UPR signaling, and in particular, the PERK-ATF4 pathway is the first to be downregulated. While PERK activation directly induces phosphorylation of Nrf2 and subsequently Nrf2 nuclear import, paradoxically, it increases translation of ATF4 mRNA and initiates up-regulation of CHOP that induces cellular apoptosis [48]. CHOP is critical for inducing oxidative stress, which disrupts the protein folding mechanism and enhances production of misfolded proteins, causing further ER stress [49]. This vicious forward cycle may account for many pathological processes, including GSK3β-induced inflammatory responses [50]. In previous studies, GSK3β could be activated by IRE1α signaling of the UPR pathway, possibly via a route involving increased autophagy of the inactive kinase phosphorylated at serine 9, which would be counteracted by the Akt-MAPK pathway [51]. In addition, ER stress in macrophages was shown to activate the PERK signaling branch of UPR to induce GSK3β activation that requires autophosphorylation of tyrosine 216 [52]. In our experimental model of ICH, GSK3β was phosphorylated at Tyr216 in the peri-hematoma area, suggesting that this hemorrhagic injury could induce catalytic activity of GSK3β, possibly via upregulation of the PERK signal pathway. Phosphorylation of GSK-3β at Tyr216 is regarded as the active form, which phosphorylates members of the Src kinase family (SFK), resulting in Nrf2 nuclear exclusion and subsequently decreasing nuclear levels of Nrf2 [53, 54]. Therefore, the balance between Nrf2-mediated adaptive and CHOP-dependent apoptotic UPR is critically important for cellular survival during ER stress. In our study, rhCDNF treatment was shown to suppress the PERK-ATF4-CHOP pathway, accompanied by decreasing phosphorylation of GSK-3β at Tyr216 and increasing nuclear Nrf2 levels, suggesting that CDNF may play a pivotal role in regulating PERK downstream branches in the cellular outcome of ER stress after the hemorrhagic stroke. This upstream regulation of Nrf2 activation by CDNF seems to be different from that of its analog MANF. Unlike CDNF, MANF-induced Nrf2 up-regulation is related to the PI3K/Akt/GSK3β pathway [47]. This discrepancy in the regulation of Nrf2 activation may be explained by the different models used; 6-OHDA injection as a model of Parkinson’s disease predominantly causes dopaminergic neuron damage. However, ICH-induced brain injury is mainly attributed to integration of aberrant immune cell function and aggravating inflammatory responses. Therefore, CDNF treatment may predominantly maintain ER and redox homeostasis to decrease GSK3β activation, which boosts Nrf2 activation to modulate the phagocytic function of microglia/macrophages in this collagenase model.

In addition to upregulating the beneficial components of microglia/macrophages, post-ICH rhCDNF administration is sufficient to limit pro-inflammatory cytokine production (TNF-α, IL-1β, IL-6, and IFN-γ), in line with previous studies. Interestingly, CDNF was shown to possess anti-inflammatory capacity by inducing the production of IL-10 that plays pivotal roles in neuroprotection after ischemia. Although IL10-mediated cytoprotection mechanisms are likely to be multifaceted, it was demonstrated to enhance polarization of microglia/macrophages towards the alternative M2 phenotype, which is well known to facilitate neuroprotection and promote brain repair after CNS injury. While MANF was shown to mitigate the production of pro-inflammatory cytokines likely via its binding to neuroplastin and then suppress NF-kβ signaling [55], the underlying mechanism of how CDNF modulates immune responses remains unclear due to the lack of knowledge of its receptor. Therefore, we need to elucidate whether this anti-inflammatory effect is a consequence of regulating innate cell responses or a mechanism involved in cytoprotection to provide a permissive microenvironment.

An increasing number of research observations highlight that microglia/macrophages can dynamically and temporally change their phenotypes in response to acute brain injury [56]. Although the supposed dichotomy between M1 and M2 phenotypes is helpful for understanding the function of immune cells in various brain diseases, controversy has begun to surround the concept of M1 versus M2 microglia/macrophage polarization. For example, in a model of TBI, canonical markers of both polarization states were highly co-expressed by the same cell [57]. Gene expression profiling of purified microglia/macrophage has significantly contributed to the characterization of these cells and understanding disease stage-specific microglia/macrophage states [58]. In the present study, we demonstrate that CDNF enhances expression of the scavenger receptors, CD36 and CD163 on activated microglia/macrophages at the hematoma accumulation stage. Using RNA-seq studies of CD11b^+^ microglia/macrophages, we confirmed that CDNF increases the capacity for erythrophagocytosis in microglia/macrophages by visualizing the mRNA expression of hemoglobin (Hba-a1, Hba-a2, Hbb, Hbb-bs), which stems from remnant mRNA in phagocytosed RBCs [59]. This suggests that CDNF-polarized microglia/macrophages exhibit enhanced erythrophagocytic activity accompanied by increased hydrogen peroxide/ROS metabolic process, providing on-demand adaptation to hemorrhagic stress. In addition to decreasing the number of activated microglia/macrophages at the subacute stage of ICH, CDNF upregulates microglial genes linked to homeostatic functions, Sall1, Ltc4s, and F11r (Fig. S9B), but it downregulates DAM genes, such as ApoE, CD63, and Gpnmb [37, 60, 61]. Homeostatic genes are enriched in matured myeloid cells that physically surveil the surrounding parenchyma and are involved in controlling neuronal excitability, synaptic activity, neurogenesis, and clearance of apoptotic cells in the healthy adult brain [62]. In contrast, ApoE or Gpnmb signaling is linked to a switch from homeostatic to a neurodegenerative microglial/myeloid phenotype and involved in severity of neurodegeneration [60]. Although DAM-specific genes were previously identified as risk factors of neurodegenerative diseases, the DAM expression program in brain myeloid cells remains to be identified [37]. We proposed that CDNF might dynamically regulate the function of brain myeloid cells from the phagocytotic phenotype during hematoma accumulation to the homeostatic state when the blood clot is cleared. However, it remains to be determined whether a trend to the homeostatic phenotype of myeloid cells is a consequence of CDNF-induced erythrophagocytosis or a reflection of decreased inflammatory responses or smaller lesioned areas.

While the I.C.V. approach in this animal model is very similar to an invasive stereotactic surgery conducted in patients, the infection, rebleeding and procedure-induced secondary brain injury might hamper this invasive surgery as a feasible application for hemorrhagic stroked patients. However, these results show that CDNF or CDNF-like smaller molecules can be developed towards disease-modifying therapy for ICH. Accordingly, in this study, we adopt intravenous delivery and demonstrate that systemic administration of rhCDNF offers beneficial effects in lesion volume caused by ICH and overall neurological function. Although the duration of exogenous CDNF in blood circulation is relatively short, it still could increase the expression of CD163 and CD36 in the peri-hematoma area, implying that CDNF in the circulation could induce peripheral monocytes/macrophages to remove hematoma at the early stage of ICH. Although the underlying mechanism by which rhCDNF in the circulation increases the scavenging function of peripheral immune cells needs further clarification, the effectiveness of systemic administration without significant untoward effects offers potential for CDNF in clinical applications.

**Fig. 8:**
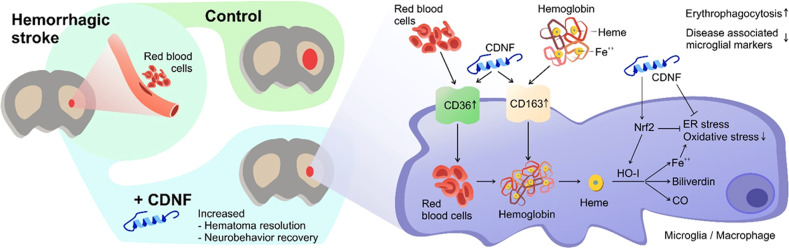
The illustration shows the schema of CDNF-augmenting hematoma resolution. In response to injury caused by ICH, CDNF administration upregulates the expressions of scavenger receptors, CD163 and CD36, in microglia/macrophages. At the same time CDNF treatment not only attenuates ER stress response, but mitigates oxidative stress by upregulating Nrf2/HO-1 levels. This process decreases a microglia type associated with neurodegenerative disease (DAM), while enhances microglial erythrophagocytosis and subsequently accelerates hematoma clearance and neurobehavior recovery.

In conclusion, we found that post-ICH delivery of rhCDNF can accelerate resolution of hemorrhagic lesions, mitigate secondary brain injury, and promote functional recovery of rats. In addition to being a promising regulator for the UPR in combination with oxidative stress, CDNF was first identified to promote hematoma-scavenging functions of microglia/macrophages by regulating the Nrf2-HO-1 signaling axis (Fig. 8). From a clinical point of view, it is of critical importance that pharmacological treatment can be systemically administered after a hemorrhagic stroke. Therefore, CDNF could be a potential therapeutic to accelerate lesion resolution and improve functional recovery in hemorrhagic stroke patients, when used in parallel with current treatment practices.

## Supplementary information

Reproducibility checklist

Supplemental material

Supplemental material (WESTERN BLOT)

Dataset 1

Dataset 2

Dataset 3

## Data Availability

The raw data for this project are available at the Gene Expression Omnibus under accession number GSE216547.
