# Supplementary material for: Augmenting hematoma-scavenging capacity of innate immune cells by CDNF reduces brain injury and promotes functional recovery after intracerebral hemorrhage
Source: Cell Death Dis. 2023 Feb 15;14(2):128. doi: 10.1038/s41419-022-05520-2 (PMC9932138; doi:10.1038/s41419-022-05520-2)
Supplement: Supplementary file 2 — Supplemental material [file 41419_2022_5520_MOESM2_ESM.pdf]

Augmenting hematoma-scavenging capacity of innate immune cells by CDNF reduces brain injury and promotes functional recovery after intracerebral hemorrhage

Tseng Kuan-Yin[1], Vassilis Stratoulias[2], Wei-Fen Hu[3], Jui-Sheng Wu[1], Vicki Wang[1], Yuan-Hao Chen[1], Anna Seelbach[2], Henri J. Huttunen [4], Natalia Kulesskaya[4], Cheng-Yoong Pang[5,6,7], Jian-Liang Chou[8], Maria Lindahl[9], Mart Saarma[9], Li-Chuan Huang[10], Mikko Airavaara[2,11*], Hock-Kean Liew[3,6,7*]

1. Department of Neurological Surgery, Tri-Service General Hospital and National Defense Medical Center, No.325, 2nd Sec., Cheng-Kung Road, Nei-Hu District, Taipei City, 114, Taiwan, R.O.C Taiwan
2. Neuroscience Center, HiLIFE, Haartmaninkatu 8, FI-00014, University of Helsinki, Finland
3. PhD Program in Pharmacology and Toxicology, Tzu Chi University, No.701, Sec.3, Zhong-Yang Road, 970 Hualien County, Hualien, Taiwan
4. Herantis Pharma Ltd, Plc, Bertel Jungin aukio 1, FI-02600 Espoo, Finland
5. Institute of Medical Sciences, Tzu Chi University, No.701, Sec.3, Zhong-Yang Road, 970 Hualien County, Hualien, Taiwan
6. Neuro-Medical Scientific Center, Hualien Tzu Chi Hospital, Buddhist Tzu Chi Medical Foundation, No.707, Sec.3, Zhong-Yang Road, 970 Hualien County, Hualien, Taiwan
7. Department of Medical Research, Hualien Tzu Chi Hospital, Buddhist Tzu Chi Medical Foundation, No.707, Sec.3, Zhong-Yang Road, 970 Hualien County, Hualien, Taiwan
8. Graduate Institute of Medical Sciences, National Defense Medical Center, Taipei, Taiwan
9. Institute of Biotechnology, HiLIFE, Viikinkaari 5D, FI-00014, University of Helsinki, Finland
10. Department of Medical Imaging, Hualien Tzu Chi Hospital, Buddhist Tzu Chi Medical Foundation, No.707, Sec.3, Zhong-Yang Road, 970 Hualien County, Hualien, Taiwan

11. Faculty of Pharmacy, Viikinkaari 5E, FI-00014, University of Helsinki, Finland

*Equal Corresponding authors: Mikko T. Airavaara & Hock-Kean Liew

Mikko Airavaara Ph.D., Faculty of Pharmacy, P.O. Box 56 (Viikinkaari 5E) 00014 University of Helsinki, Finland.

Email: mikko.airavaara@helsinki.fi

Hock-Kean Liew Ph.D., Department of Medical Research, Hualien Tzu Chi Hospital, Buddhist Tzu Chi Medical Foundation, Hualien, Taiwan.

Email: hockkean@tzuchi.com.tw; hockkean@hotmail.com

Supplement Exp.

RNA sequencing from the hemorrhagic striatum of Wt and *Cdnf*$^{-/-}$ mice

Mice were sacrificed at 6 hours post-ICH induction and perfused with 0.9% saline solution before collecting tissue (n=3 for Wt, n=3 for *Cdnf*$^{-/-}$). RNA was extracted from punch samples of tissue taken from the hemorrhagic striatum with 1 mm thick sections from positions A/P -0.9 to +0.1. RNA was extracted with Trizol reagent and treated with DNase (#1906, Ambion). RNA purity and quantification were checked using SimpliNano™ - Biochrom Spectrophotometers (Biochrom, MA, USA). RNA degradation and integrity were monitored by Qsep 100 DNA/RNA Analyzer (BiOptic Inc., Taiwan). A total amount of 1 μl RNA per sample was used as input material for RNA sample preparations. Following the manufacturer's recommendations, sequencing libraries were generated using the KAPA mRNA HyperPrep Kit (KAPA Biosystems, Roche, Basel, Switzerland), and index codes were added to attribute sequences to each sample. Briefly, mRNA was purified from total RNA using magnetic oligo-dT beads. Captured mRNA was fragmented by incubating it at 94 °C in the presence of magnesium in KAPA Fragment, Prime, and Elute Buffer (1x). First-strand cDNA was synthesized using random hexamer priming. Combined second-strand synthesis and A-tailing, which converts the cDNA: RNA hybrid into double-stranded cDNA (dscDNA), was used to incorporate dUTP into the second cDNA strand, and then dAMP was added to the 3' ends of the resulting dscDNA. dsDNA adapters with 3'dTMP overhangs were ligated to library insert fragments to generate library fragments carrying the adapters. To select cDNA fragments of 300~400bp in length, fragments were purified with the KAPA Pure Beads system (KAPA Biosystems, Roche, Basel, Switzerland). The library carrying appropriate adapter sequences at both ends was amplified using KAPA HiFi HotStart ReadyMix (KAPA Biosystems, Roche, Basel, Switzerland) and library amplification primers. The strand marked with dUTP was not amplified, allowing strand-specific sequencing. Lastly, PCR products were purified using the KAPA Pure Beads system, and the library quality was assessed using the Qsep 100 DNA/RNA Analyzer (BiOptic Inc., Taiwan). The RNA-seq

data (Supplement data 2) has been deposited in the RNA-Seq database at Biotools-rat-RNA pre library under accession number PRJNA745332.

Evaluation of tissue penetration of labeled rhCDNF

rhCDNF was labeled with Alexa Fluor® 488 dye using a Microscale Protein Labeling Kit (A30006, Invitrogen, USA) according to the manufacturer's instructions. The Alexa Fluor® 488 dye-labeled rhCDNF (5 μg), with fluorescence excitation and emission maxima of approximately 494 and 519 nm, was administered i.c.v. one hour after ICH. After 6 hours post-injection of the fluorescently labeled rhCDNF, rats were re-anesthetized with pentobarbital (50 mg/kg i.p.). Brains were removed immediately and sectioned to 20 μm thickness with a cryostat. Each slice was double-stained with an anti-CDNF antibody to confirm entrance of rhCDNF peptide into the brain striatum. Sections were incubated overnight at 4°C with CDNF primary antibody (1:100; Catalog No. U4757, Sigma, CA, USA). Slices were then washed with PBS and incubated for 1 hour with a secondary antibody (anti-rabbit-Rhodamine, Jackson ImmunoResearch, West Grove, PA, USA) at room temperature. After rinsing with PBS buffer, slices were examined under a fluorescence microscope. After counter-staining nuclei with DAPI, slides were washed and mounted on coverslips with an anti-fading mounting medium (VECTASHIELD®, CA, USA). Presence of labeled rhCDNF in the striatum was evaluated under a fluorescence microscope.

Oxidative damage of proteins by Oxyblot

To determine the amount of carbonylated proteins, we used the OxyBlot Protein Oxidation Detection Kit (Chemicon International, S7150), as described by the manufacturer's instructions. Tissues or cells were lysed in RIPA lysis buffer containing a protease inhibitor cocktail (Roche). 5 mg of tissue/cell lysate in 10 ml lysis buffer with 12% SDS was added to 10 μl of 10 mM 2,4-dinitrophenylhydrazine (DNPH) solution for 15 minutes at RT for derivatizing the sample. As a control, 10 μl of a duplicate

sample was added to 10 μl of a control solution not containing DNPH. Both samples were subsequently incubated with 7.5 μl of a neutralization solution to stop the derivatization reaction. Proteins were separated by 12% SDS-PAGE and transferred to PVDF membranes. DNP-containing proteins were immunostained using rabbit anti-DNP antiserum (1:2000) and goat anti-rabbit IgG conjugated to horseradish peroxidase (HRP) (1:2000). Blots were visualized by an enhanced chemiluminescence (ECL) system. The results were quantified by Image J software.

Assessment of cytokines

Ipsilateral striatal tissues were used to evaluate the cytokine expression by an enzyme-linked immunosorbent assay (ELISA) kits for TNFα (DY510), IL-1β (DY501), IL-6 (DY506), IL-10 (DY522), and IFNγ (R & D Systems, Minneapolis, MN, USA) according to the manufacturer's instructions, respectively. During quantification, the cytokines (TNFα, IFN-γ, IL-1β, IL-6, IL-10) were normalized to 50 μg of protein.

Evaluation of physiological parameters

Under urethane (1.0 g/kg body weight, i.p., Sigma-Aldrich) anesthesia, a femoral artery was cannulated with a PE-50 polyethylene tube for fluid supplementation and monitoring of arterial blood pressure and blood gas. Arterial blood pressure and heart rate were recorded through an amplifier (MP36, BIOPAC system, CA, USA) and stored in a PC. Body temperature (rectal temperature) was automatically maintained at 37.5 ± 0.5°C by a rectal temperature sensor and a heating pad (CMA-150, Sweden). Physiological parameters including PaO2, sodium, potassium, glucose, lactate, and hemoglobin) were measured 10 minutes pre- (Pre-op) and 3, 6, and 24 hours post-ICH.

Statistical Analysis

Values are presented as mean ± S.E.M. Unpaired t-test, and one-or two-way analysis of variance (ANOVA) with post hoc Bonferroni tests were used for statistical analysis. A statistically significant difference was defined as $p<0.05$.

SUPPLEMENTARY MATERIALS

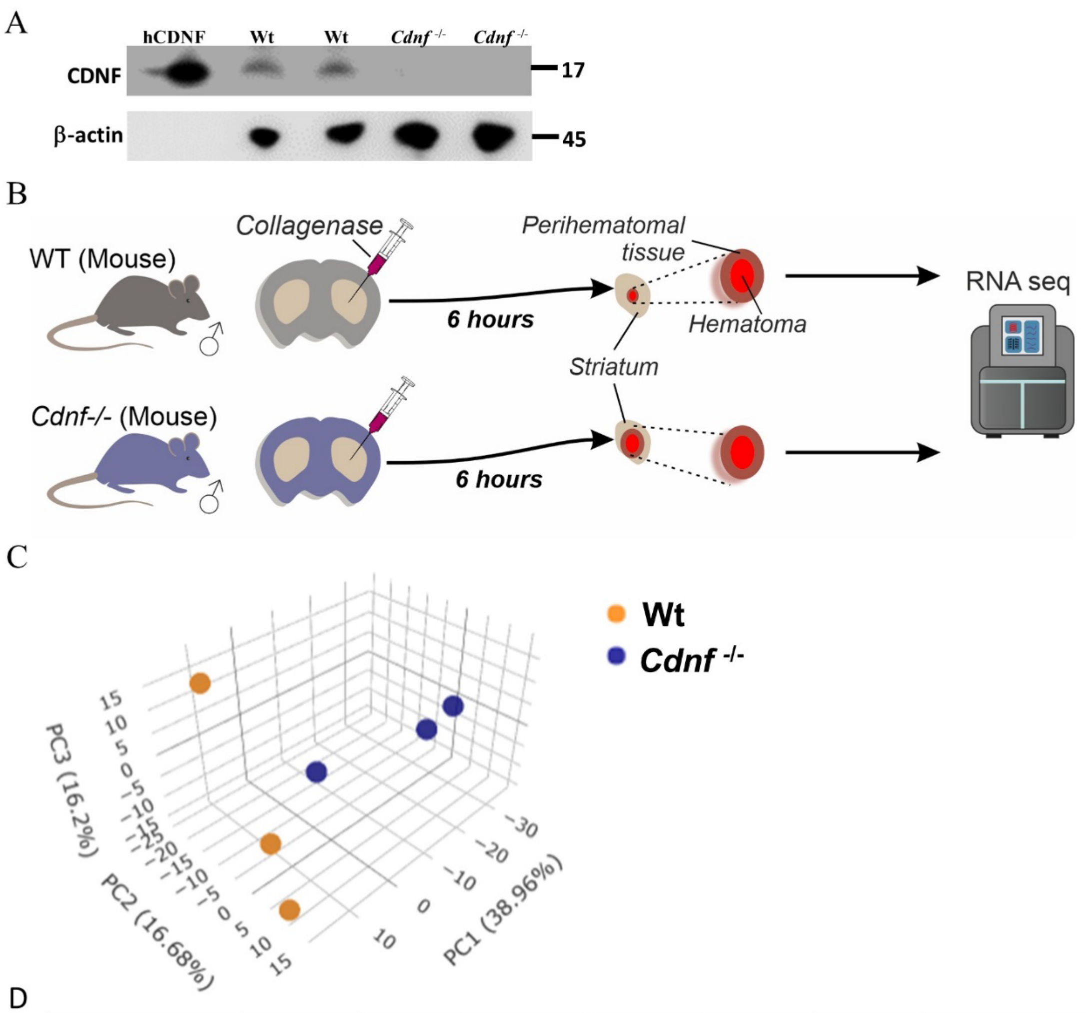

D

ensembl_gene_id	entrezgene	symbol	baseMean	log2Fold Change	pvalue	padj	Cdnf-ko mice	WT mice
ENSMUSG00000039496	227526	Cdnf	14.0832502	-7.27	1.02E-08	0.000373	0	26.99
ENSMUSG00000095041			2460.45804	1.39	7.31E-08	0.00134	4091.34	1560.75
ENSMUSG00000005413	15368	Hmox1	568.124691	-0.92	0.0000022	0.0269	627.41	1186.44

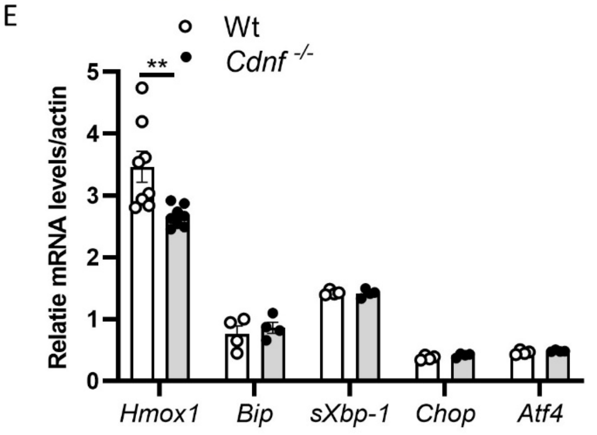

Fig. S1. **(A)** Western blot analysis of striatum lysates confirms that CDNF protein is not expressed

in the striatal area of *Cdnf*$^{-/-}$ mouse brain. Recombinant human CDNF protein (rhCDNF) was used as a positive control and anti-actin antibody for normalization of total protein content. **(B)** Schematic overview of the experimental design. **(C)** Principal component analysis (PCA) of the transcriptomes between two groups **(D)** Top hits from RNA sequencing analysis performed to find genes expressed differentially in the hemorrhagic striatum of *WT* (n=3) and *Cdnf*$^{-/-}$ (n=3) mice. The analysis was carried out with DESseq2. Genes were identified as differentially expressed genes (DEGs) only when the fold difference between the two groups was greater than 1.5, and adjusted p values were lower than 0.05. The level of up/downregulation (log2 fold change) and the p-value adjusted for multiple testing are shown. **(E)** qPCR analysis of Hmox1, Bip, sXbp-1, Chop and Atf4 mRNA levels in the peri-hematoma striatum at 6h after hemorrhagic injury (n=3). **P < 0.01 by multiple comparisons using the Holm-Šidák method. Mean ± S.E.M. is shown.

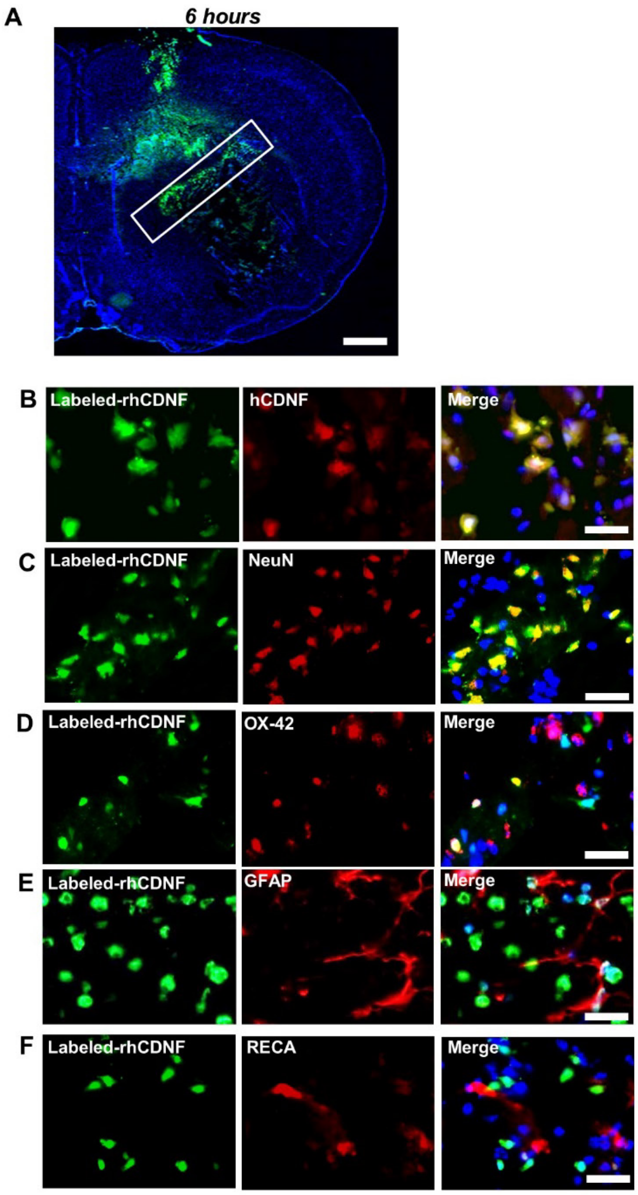

Fig. S2. **Fluorescently labeled rhCDNF is detected in histological sections of hemorrhagic striatum.** Alexa Fluro® 488-labeled-rhCDNF was delivered i.c.v. 1 hour after unilateral collagenase-ICH. Animals were sacrificed 6 hours after injection of the labeled-rhCDNF, which is distributed in the hemorrhagic striatum **(A)** and co-localized in hCDNF-expressed cells **(B)**. A yellowish color in the merge **(C-E)** indicates co-localization of rhCDNF expression in neurons (NeuN, **C**), microglia (OX-42, **D**) and astrocytes (GFAP, **E**). However, rhCDNF expression is not co-localized in RECA-labeled endothelial cells **(F)**.

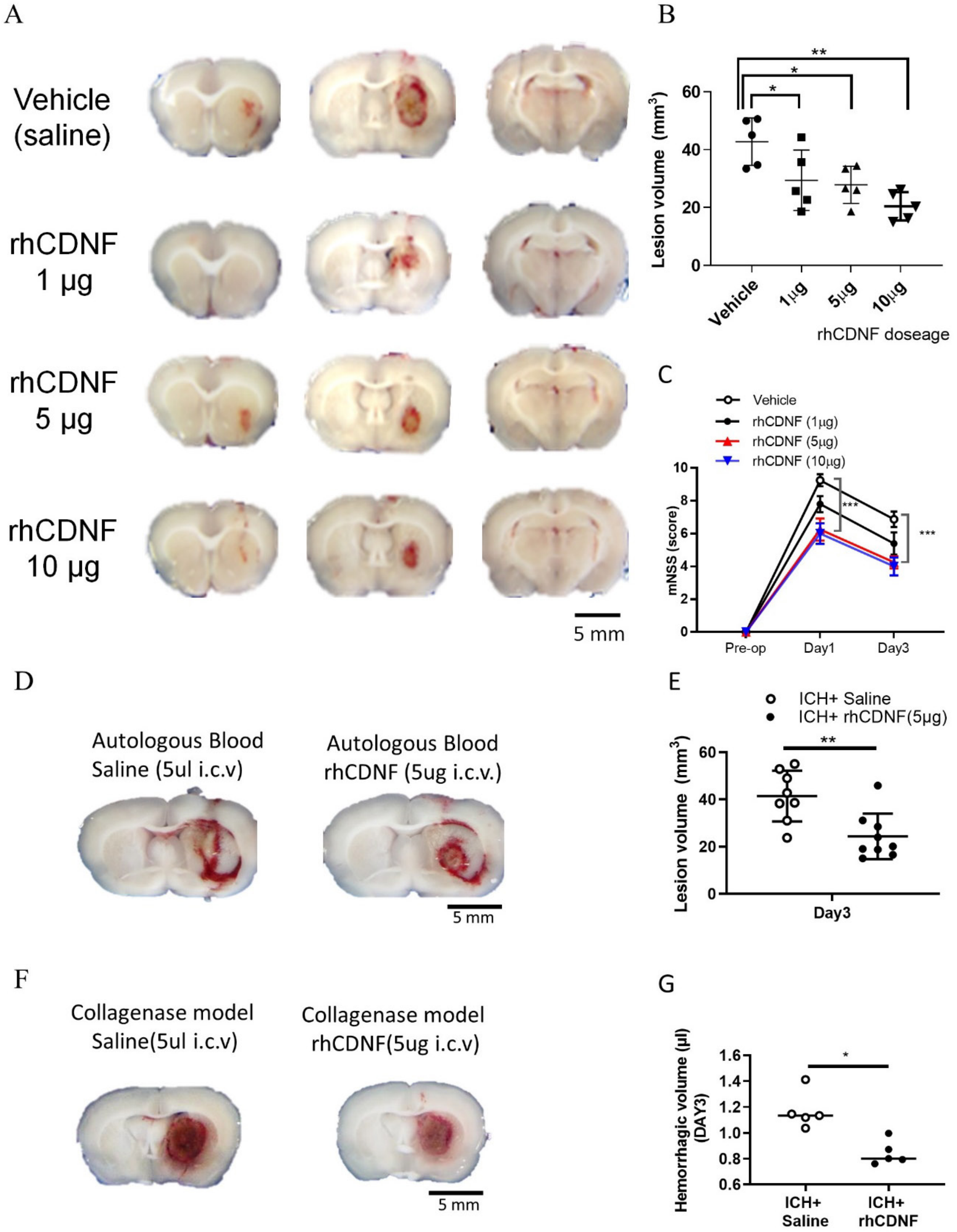

Fig. S3. **Effects of rhCDNF posttreatment on hemorrhagic and lesion volume and mNSS of rats with ICH induced by collagenase or autologous blood injection. (A-C)** Collagenase-induced ICH rats were injected with saline or rhCDNF (1, 5, and 10 µg, i.c.v) at one hour post-ICH. **(A)** Photographs of representative brain sections showing the sizes of hemorrhagic areas on days 3 after

ICH insult. **(B)** Lesion volume on days 3 post-ICH was determined by morphometric measurement. *p <0.05, ***p <0.001 vs. ICH + saline group by one way ANOVA followed by Bonferroni correction. **(C)** The mNSS were examined before and 1 to 3 d after ICH insult. ***p< 0.001 by Dunnett's multiple comparisons test, following two-way ANOVA [effect of treatment: $F_{(3,69)} = 16.50$, P <0.0001]. **(D, E)** Autologous blood infusion-induced ICH rats were injected with saline or rhCDNF (5 µg, i.c.v). Brain sections show the sizes of hemorrhagic areas in the ICH+ saline and the ICH+ rhCDNF groups on Day 3 **(D)**. Histograms show that the ICH+ rhCDNF significantly reduced the injured area on Day 3. **p< 0.01 by Student's t-test. **(E)**. **(F)** Representative coronal sections (2 mm thickness) show brain hemorrhagic areas of rats sacrificed 3 days after ICH. **(G)** Hemorrhagic volume on day 3 (n=5, each group) post-ICH was determined by spectrophotometric assay. Mean ± S.E.M. is shown. Scale bars: 5 mm.

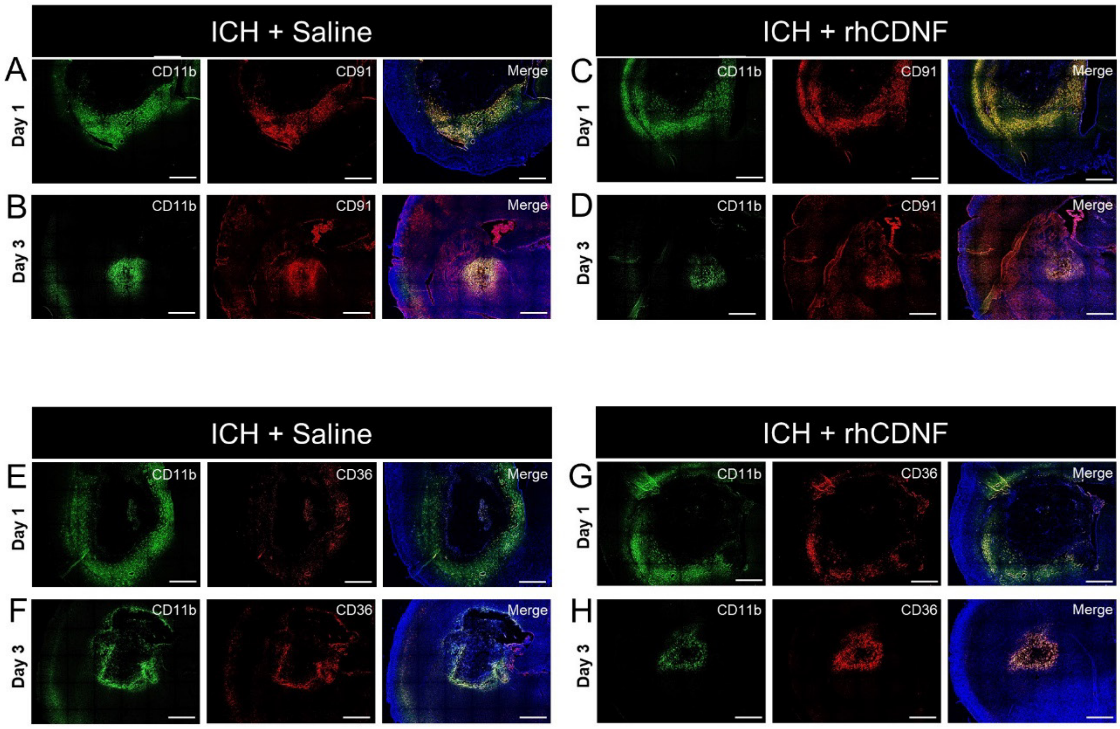

Fig. S4. **(A-D)** Immunofluorescent staining of cells in hemorrhagic striatum with anti-CD11b (green), anti-CD91 (red), and counterstained with DAPI (blue). Immunofluorescence pictures showing co-localization of CD91 and CD11b in the hemorrhagic striatum on 1 day **(A, C)** but not 3 days **(B, D)** post-ICH. **(E-H)** Figures show immunofluorescent staining of cells in hemorrhagic striatum with anti-CD11b (green), anti-CD36 (red), and counterstained with DAPI (blue). The merged images of the overlay of CD36 together with CD11b$^+$ microglia/macrophages were shown as yellow, and nuclei were stained with DAPI (blue) on 1 day **(E, G)** and 3 days **(F, H)** after ICH. Scale bars: 50 μL.

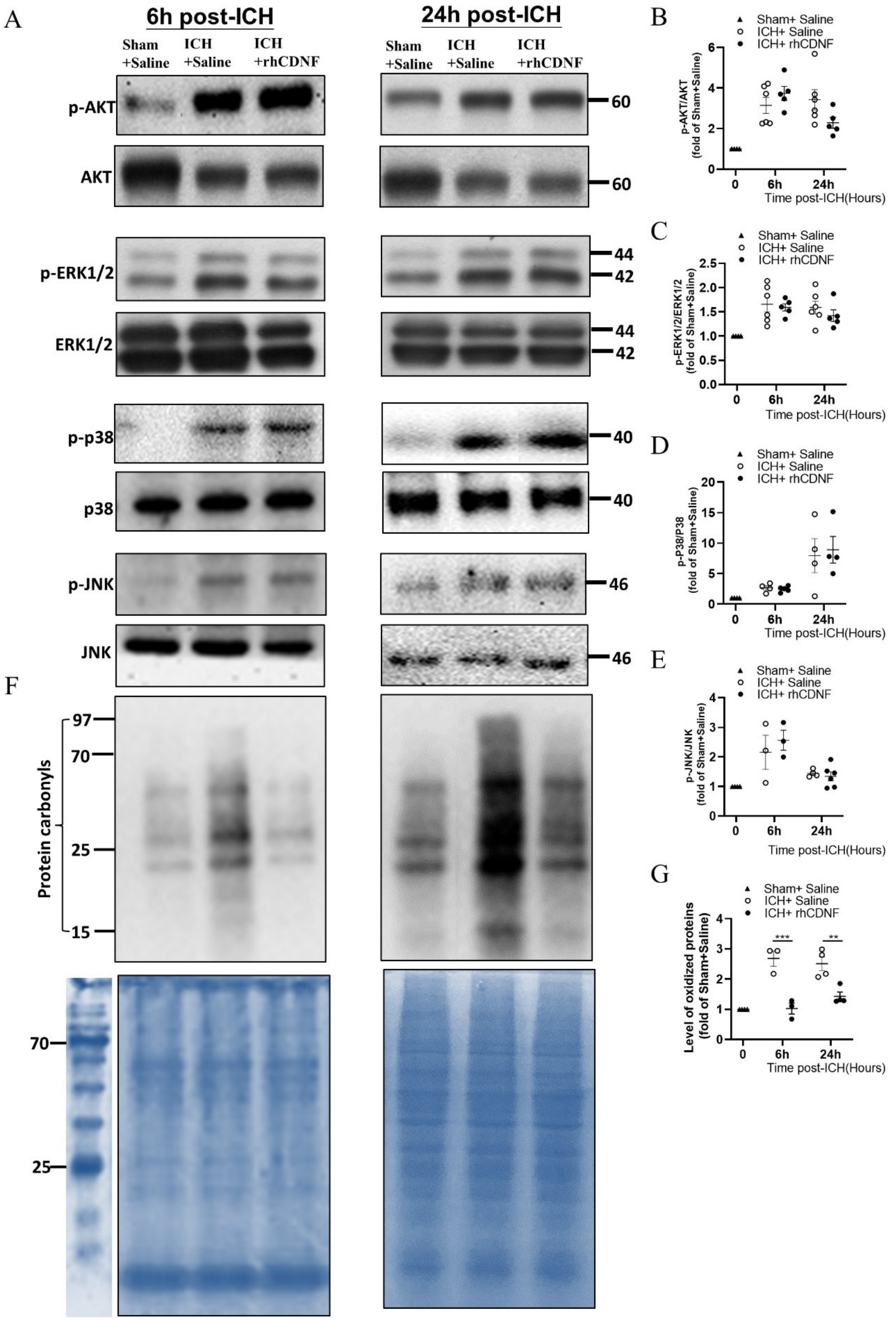

A
6h post-ICH
24h post-ICH
Sham +Saline
ICH +Saline
ICH +rhCDNF
p-AKT
AKT
p-ERK1/2
ERK1/2
p-p38
p38
p-JNK
JNK
60
44
42
40
46
F
Protein carbonyls
97
70
25
15
B
C
D
E
G
Sham+ Saline
ICH+ Saline
ICH+ rhCDNF
p-AKT/AKT (fold of Sham+Saline)
p-ERK1/2/ERK1/2 (fold of Sham+Saline)
p-P38/P38 (fold of Sham+Saline)
p-JNK/JNK (fold of Sham+Saline)
Level of oxidized proteins (fold of Sham+Saline)
Time post-ICH(Hours)
0
6h
24h
***
**

Fig. S5. **(A)** Western blot images with expression of p-AKT, AKT, p-ERK1/2, ERK1/2, p-P38, P38, p-JNK and JNK in the hemorrhagic striatum of indicated groups of rats receiving sham + saline, ICH + saline or ICH + rhCDNF. **(B-E)** Data points show the relative protein expression levels of p-AKT/AKT, p-ERK1/2/ERK, p-P38/P38, p-JNK/JNK in the hemorrhagic striatum of indicated groups of rats receiving sham + saline, ICH + saline or ICH + rhCDNF. **(F)** Hemorrhagic striatum tissue protein carbonyls were detected by Western blot analysis using the Oxyblot protein oxidation detection kit. Upper: Western blot images of carbonylated protein; Down: polyacrylamide resolving gels (12%, w/v) loaded with the same quantity of samples were electrophoresed and stained with Coomassie Brilliant Blue R250 as the loading control. **(G)** Quantitative values were calculated as the ratio of densities of the respective protein to total balanced protein. The results are presented as a fold change increase compared to sham + saline control.

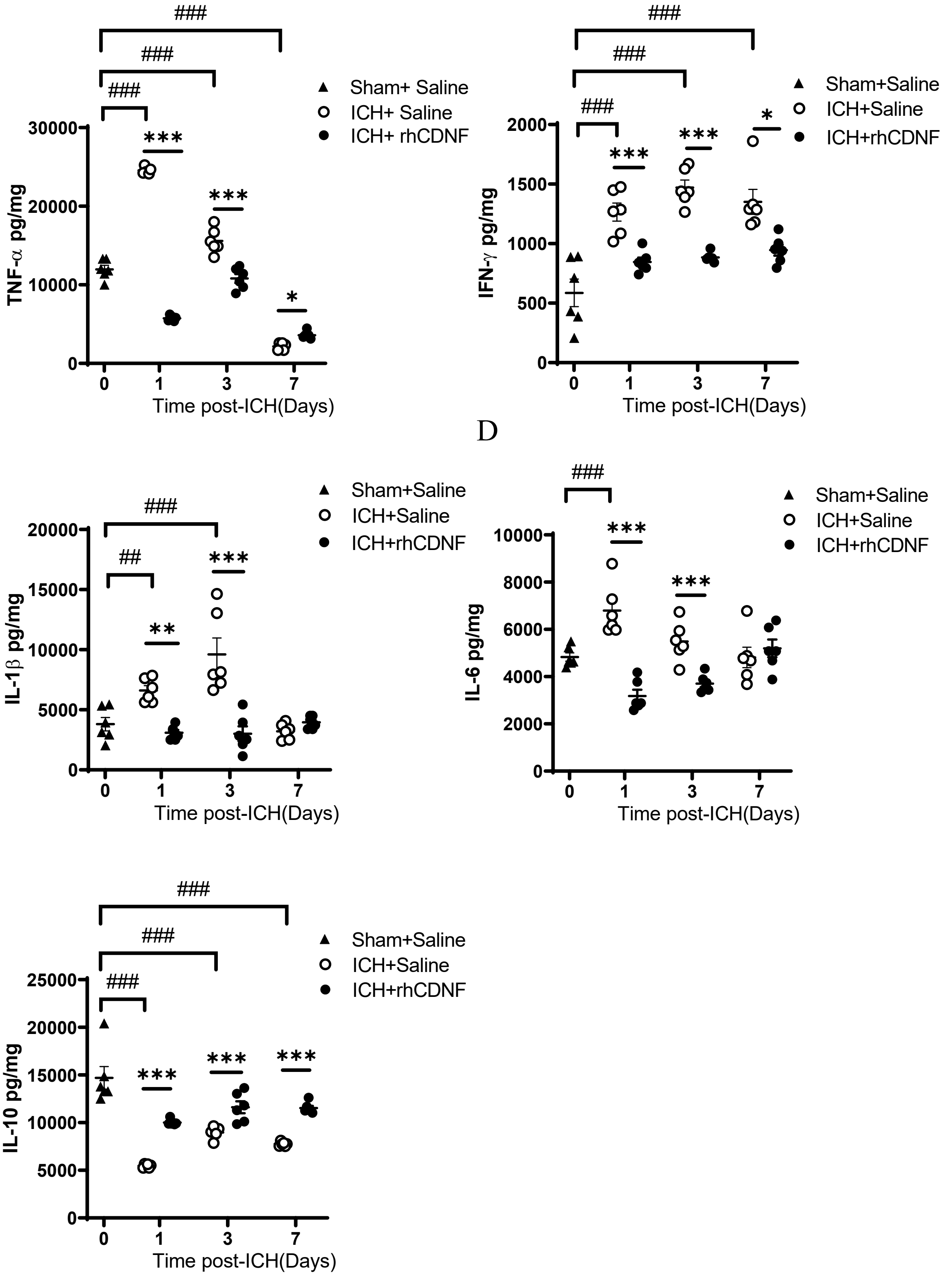

###
###
###
***
***
*
Sham+ Saline
ICH+ Saline
ICH+ rhCDNF
TNF-α pg/mg
30000
20000
10000
0
0
1
3
7
Time post-ICH(Days)
###
###
###
***
***
*
Sham+Saline
ICH+Saline
ICH+rhCDNF
IFN-γ pg/mg
2000
1500
1000
500
0
0
1
3
7
Time post-ICH(Days)
D
###
##
**
***
Sham+Saline
ICH+Saline
ICH+rhCDNF
IL-1β pg/mg
20000
15000
10000
5000
0
0
1
3
7
Time post-ICH(Days)
###
***
***
Sham+Saline
ICH+Saline
ICH+rhCDNF
IL-6 pg/mg
10000
8000
6000
4000
2000
0
0
1
3
7
Time post-ICH(Days)
###
###
###
***
***
***
Sham+Saline
ICH+Saline
ICH+rhCDNF
IL-10 pg/mg
25000
20000
15000
10000
5000
0
0
1
3
7
Time post-ICH(Days)

Fig. S6. Levels of TNF-α **(A)**, IFN- γ **(B)**, IL-1β **(C)**, IL-6 **(D)** and IL-10 **(E)** in hemorrhagic striatum tissues. After ICH, the ipsilateral striatal tissues were collected at the indicated time points. The content of cytokines in tissues was measured by ELISA. *$p<0.05$, **$p<0.01$, ***$p<0.001$ vs. ICH + saline group, ##$p < 0.01$, ###$p < 0.001$ vs. sham + saline group. Data were analyzed as repeated measures by one way ANOVA followed by Bonferroni correction.

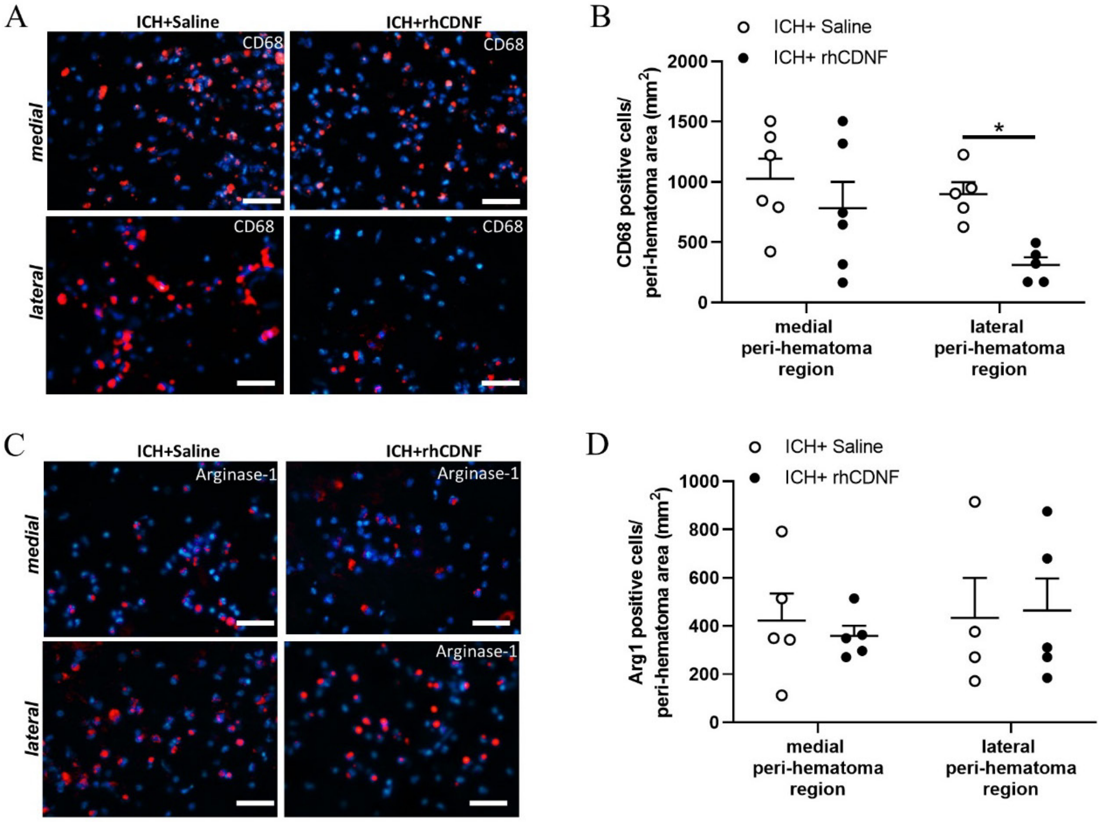

Fig. S7. **(A)** Representative CD68-positive staining of microglia/macrophages in medial and lateral regions of peri-hematoma areas. **(B)** Statistical measurement of $CD68^+$ microglia/macrophages subjected to ICH injury in the ICH+ saline and ICH+ rhCDNF groups. Two-way ANOVA + post-hoc Bonferroni test, *$p < 0.05$ in comparison to the ICH+ saline group. **(C)** Representative Arginase 1-positive staining of microglia/macrophages in the medial and lateral regions of peri-hematoma area. **(D)** Number of Arginase 1^+ microglia/macrophages subjected to ICH injury in the ICH+ saline and ICH+ rhCDNF groups.

A

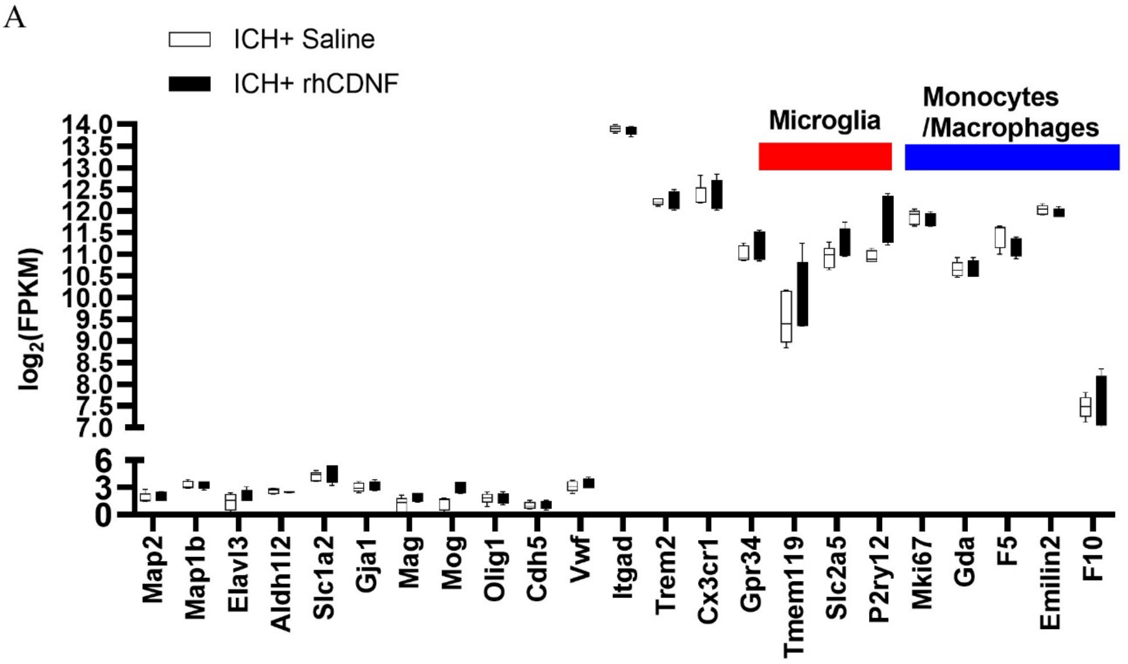

B

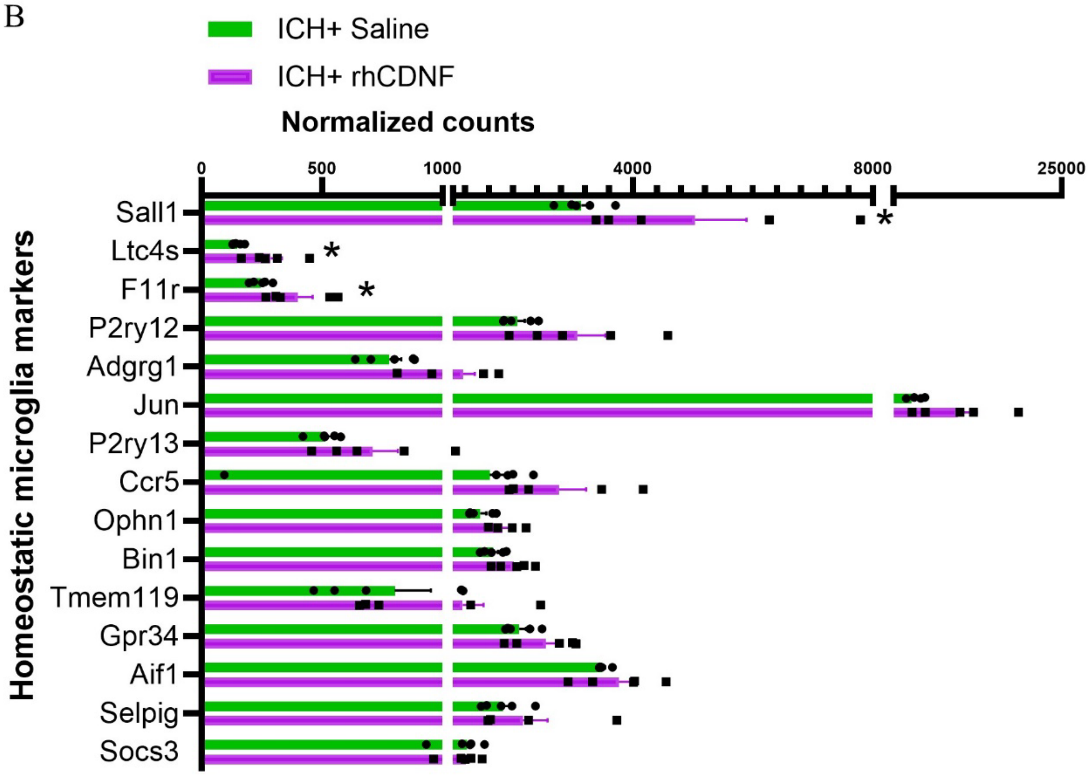

Fig. S8. **(A)** Expression of cell-specific marker genes in purified $CD11b^+$ cells. Specific markers for neurons, astrocytes, oligodendrocytes, endothelial cells, and myeloid cells were compared. $CD11b^+$ cells express high levels of microglia- and monocyte/macrophage-specific genes. Data are expressed

as log_2(FPKM). **(B)**. CD11b+ cells from rhCDNF-treated group express high transcript number of homeostatic microglial genes (Sall1, Ltc4s and F11r). n=5. * P<0.05, Multiple-Mann-Whitney tests.

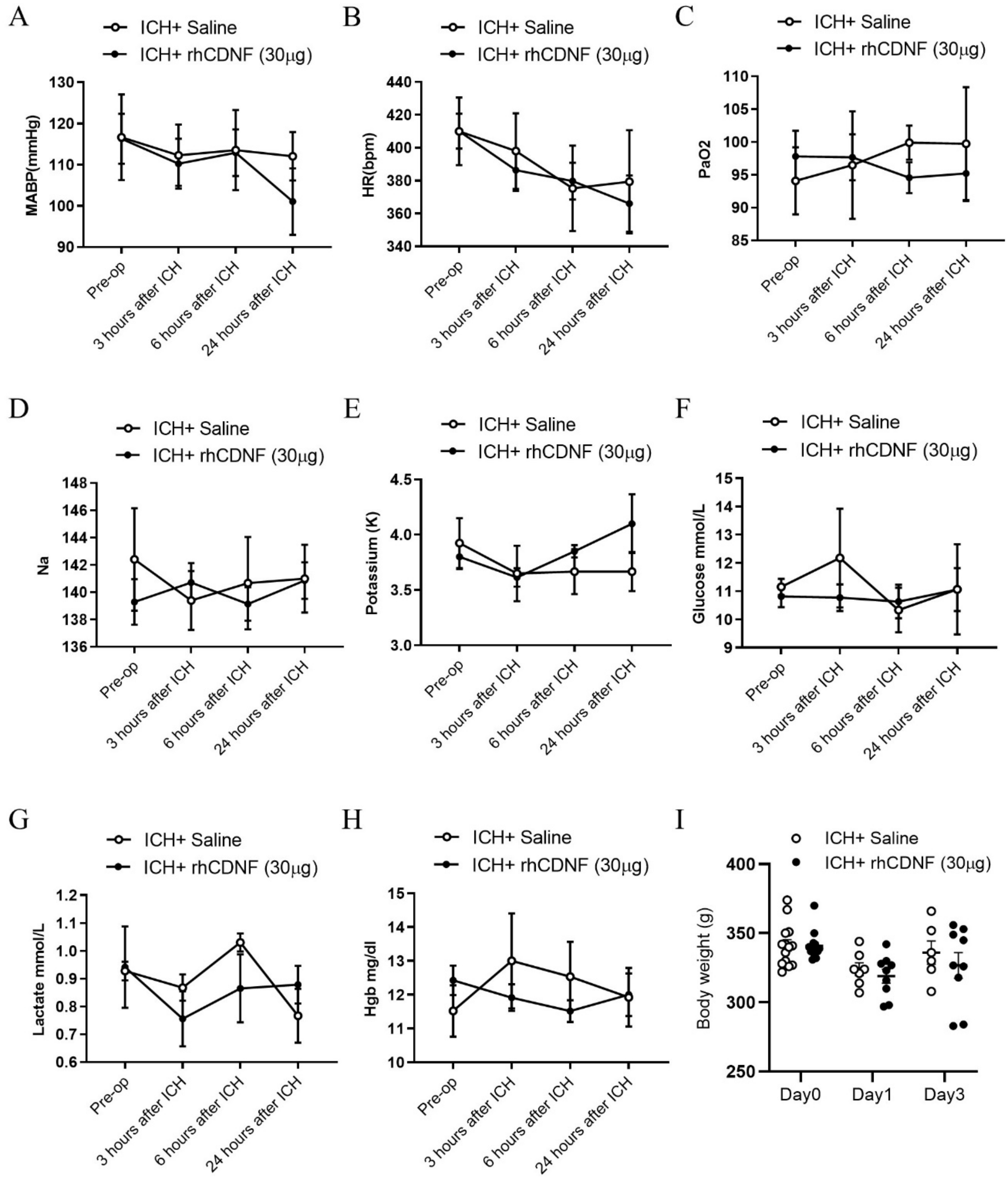

Fig. S9. Comparison of physiological parameters in ICH+ saline and ICH+ rhCDNF (30 µg, i.v.). **(A)** Mean arterial blood pressure (MABP), **(B)** Heart rate (HR), **(C)** Partial pressure of oxygen (PaO2), **(D)** Sodium (Na), **(E)** Potassium (K), **(F)** Glucose, **(G)** Lactate, and **(H)** Hemoglobin (Hgb) were measured up to 24 hours after treatment with saline or rhCDNF in the group of rats. **(I)** Body weight was measured up to 3 days after treatment with saline or rhCDNF in the group of rats.

Table. S1. Table of qPCR primers used in the study.

	Forward primer	Reverse primer
Cdnf	AAAGAAAACCGCCTGTGCTA	TCATTTTCCACAGGTCCACA
Gapdh	CCACCATGGAGAAGGC	ATGGACTGTGGTCATGA
Bip	TTCAGCCAATTATCAGCAAACTCT	TTTTCTGATGTATCCTCTTCACCAGT
sXbp1	CCTGGAAAGGGCTCAACAC	CAGTTCTTCTCTGTGGAGCTGA
Atf4	ATGGCCGGCTATGGATGAT	CGAAGTCAAACTCTTTCAGATCCATT
Chop	CCAACAGAGGTCACACGCAC	TGACTGGAATCTGGAGAGCGA
Hmox1	CGTGCAGAGAATTCTGAGTTC	AGACGCTTTACGTAGTGCTG
Actin	ACCGCTCGTTGCCAATAGTGATGA	TGAGAGGGAAATCGTGCGTGACAT
ApoE	TTGGTCCCATTGCTGACAG	ACCGTCAGTTCCTGTGTGAC
Gpnmb	AGAGTCAAGCCCTGACTGGC	GAAGAGTGGGTTCCCAGTCA
Cd63	GCCCTTGGAATTGCTTTTGTCG	CATCACCTCGTAGCCACTTSTG
Ube2i	AGCTACGGATGCTTTTCAAAGA	CAGAAGGATACACGTTTGGATGA

Supplemental data S2. RNA-sequencing data of Wt and Cdnf$^{-/-}$ mice of hemorrhagic stroke.

Supplemental data S3. RNA-sequencing data of isolated $CD11b^+$ cells in the hemorrhagic striatum.

Supplemental data S4. GO over-representation analysis based on isolated $CD11b^+$ cells in the hemorrhagic striatum.
