# Supplementary material for: Augmenting hematoma-scavenging capacity of innate immune cells by CDNF reduces brain injury and promotes functional recovery after intracerebral hemorrhage
Source: Cell Death Dis. 2023 Feb 15;14(2):128. doi: 10.1038/s41419-022-05520-2 (PMC9932138; doi:10.1038/s41419-022-05520-2)
Supplement: Supplementary file 3 — Supplemental material (WESTERN BLOT) [file 41419_2022_5520_MOESM3_ESM.pptx]

## Slide 1
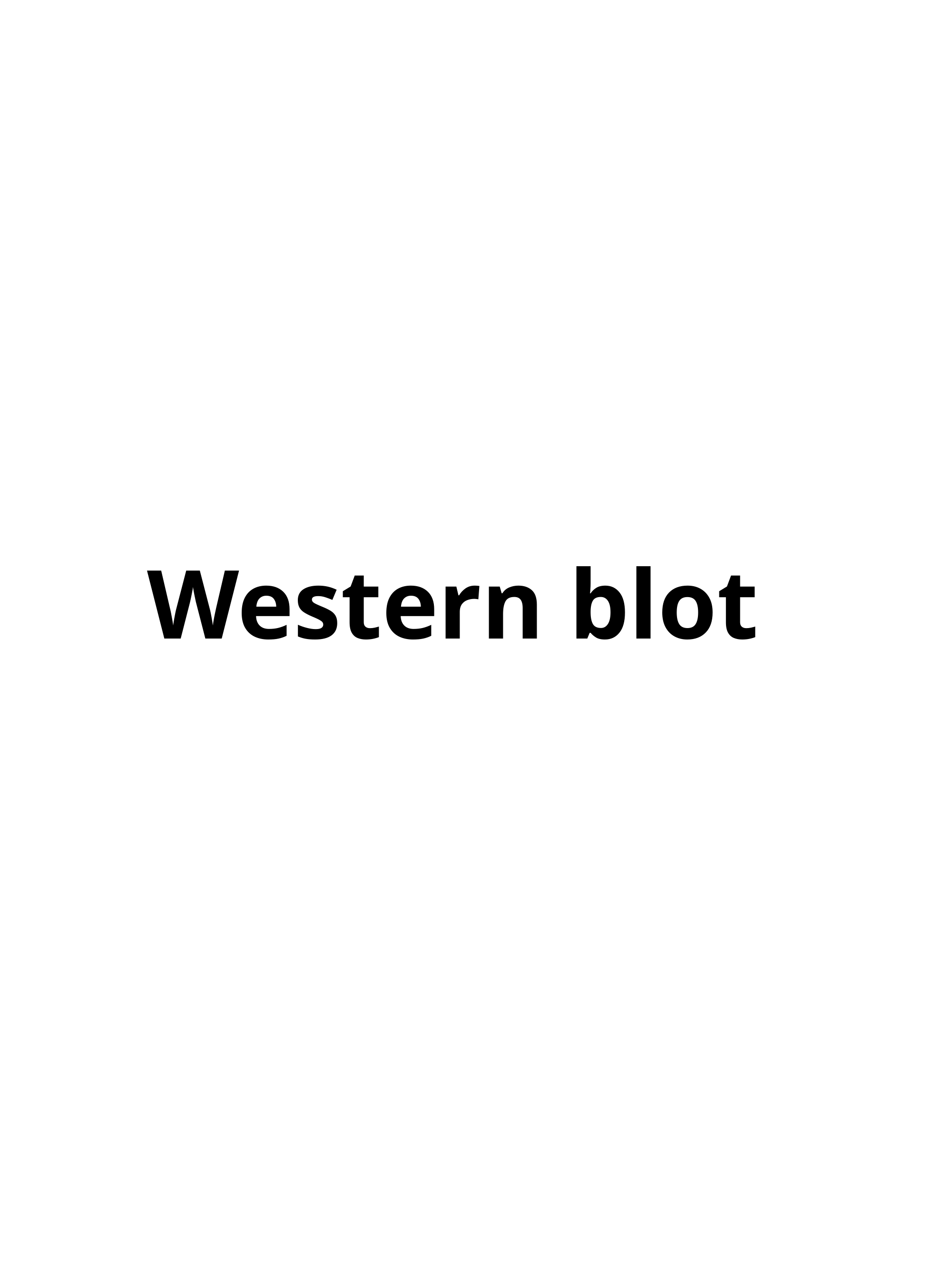

# Western blot

## Slide 2
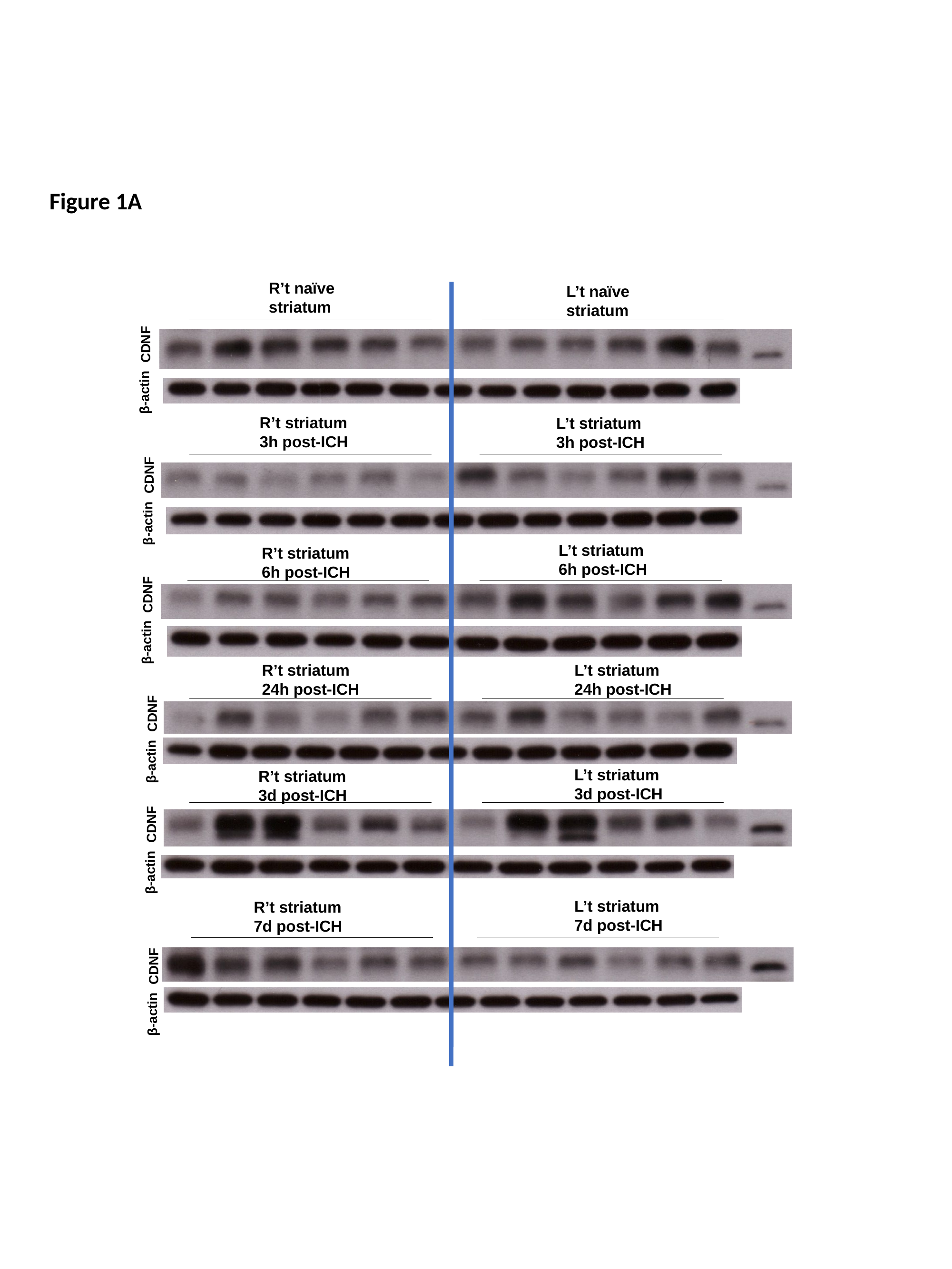

Figure 1A
R’t naïve
striatum
L’t naïve
striatum
CDNF
β-actin
R’t striatum
3h post-ICH
L’t striatum
3h post-ICH
CDNF
β-actin
L’t striatum
6h post-ICH
R’t striatum
6h post-ICH
CDNF
β-actin
R’t striatum
24h post-ICH
L’t striatum
24h post-ICH
CDNF
β-actin
L’t striatum
3d post-ICH
R’t striatum
3d post-ICH
CDNF
β-actin
L’t striatum
7d post-ICH
R’t striatum
7d post-ICH
CDNF
β-actin

## Slide 3
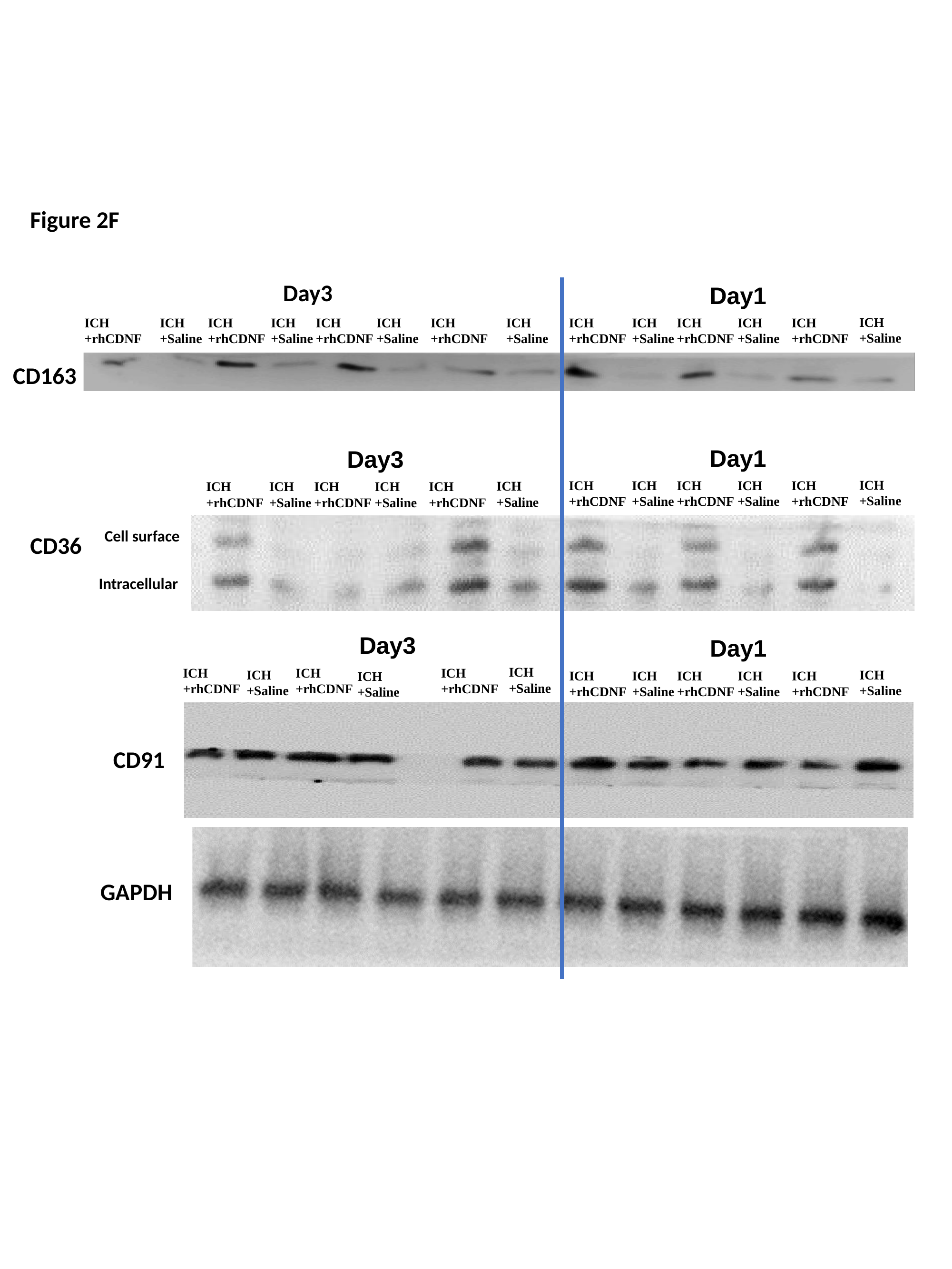

Figure 2F
Day3
Day1
ICH
+Saline
ICH
+rhCDNF
ICH
+rhCDNF
ICH
+rhCDNF
ICH
+rhCDNF
ICH
+rhCDNF
ICH
+rhCDNF
ICH
+rhCDNF
ICH
+Saline
ICH
+Saline
ICH
+Saline
ICH
+Saline
ICH
+Saline
ICH
+Saline
CD163
Day1
ICH
+Saline
ICH
+rhCDNF
ICH
+Saline
ICH
+rhCDNF
ICH
+Saline
ICH
+rhCDNF
Day3
ICH
+Saline
ICH
+rhCDNF
ICH
+Saline
ICH
+rhCDNF
ICH
+Saline
ICH
+rhCDNF
Cell surface
CD36
Intracellular
Day3
Day1
ICH
+Saline
ICH
+rhCDNF
ICH
+Saline
ICH
+rhCDNF
ICH
+Saline
ICH
+rhCDNF
ICH
+Saline
ICH
+rhCDNF
ICH
+rhCDNF
ICH
+rhCDNF
ICH
+Saline
ICH
+Saline
CD91
GAPDH

## Slide 4
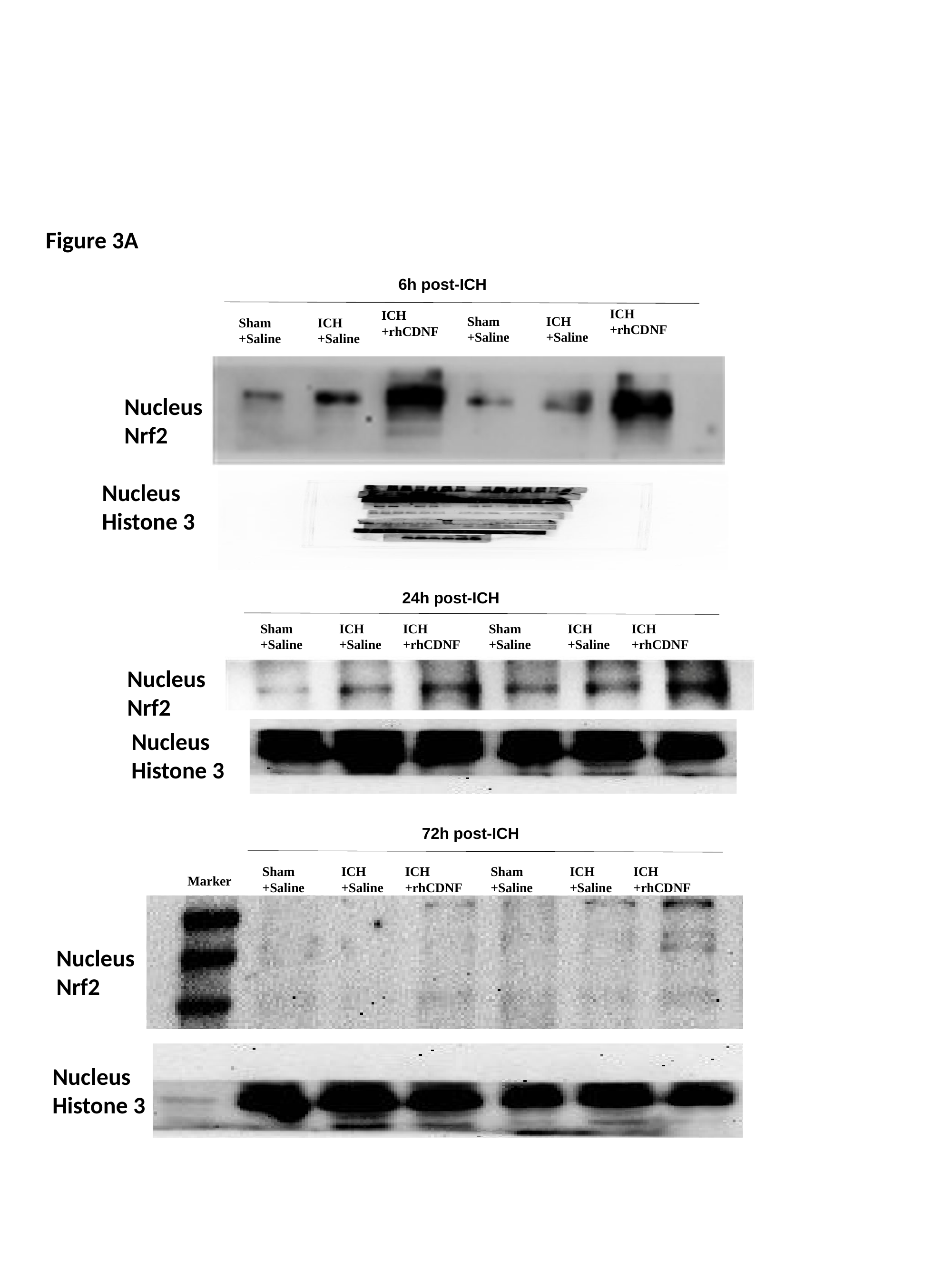

Figure 3A
6h post-ICH
ICH
+rhCDNF
ICH
+rhCDNF
Sham
+Saline
ICH
+Saline
Sham
+Saline
ICH
+Saline
Nucleus
Nrf2
Nucleus
Histone 3
24h post-ICH
Sham
+Saline
ICH
+Saline
ICH
+rhCDNF
Sham
+Saline
ICH
+Saline
ICH
+rhCDNF
Nucleus
Nrf2
Nucleus
Histone 3
72h post-ICH
Sham
+Saline
ICH
+Saline
ICH
+rhCDNF
Sham
+Saline
ICH
+Saline
ICH
+rhCDNF
Marker
Nucleus
Nrf2
Nucleus
Histone 3

## Slide 5
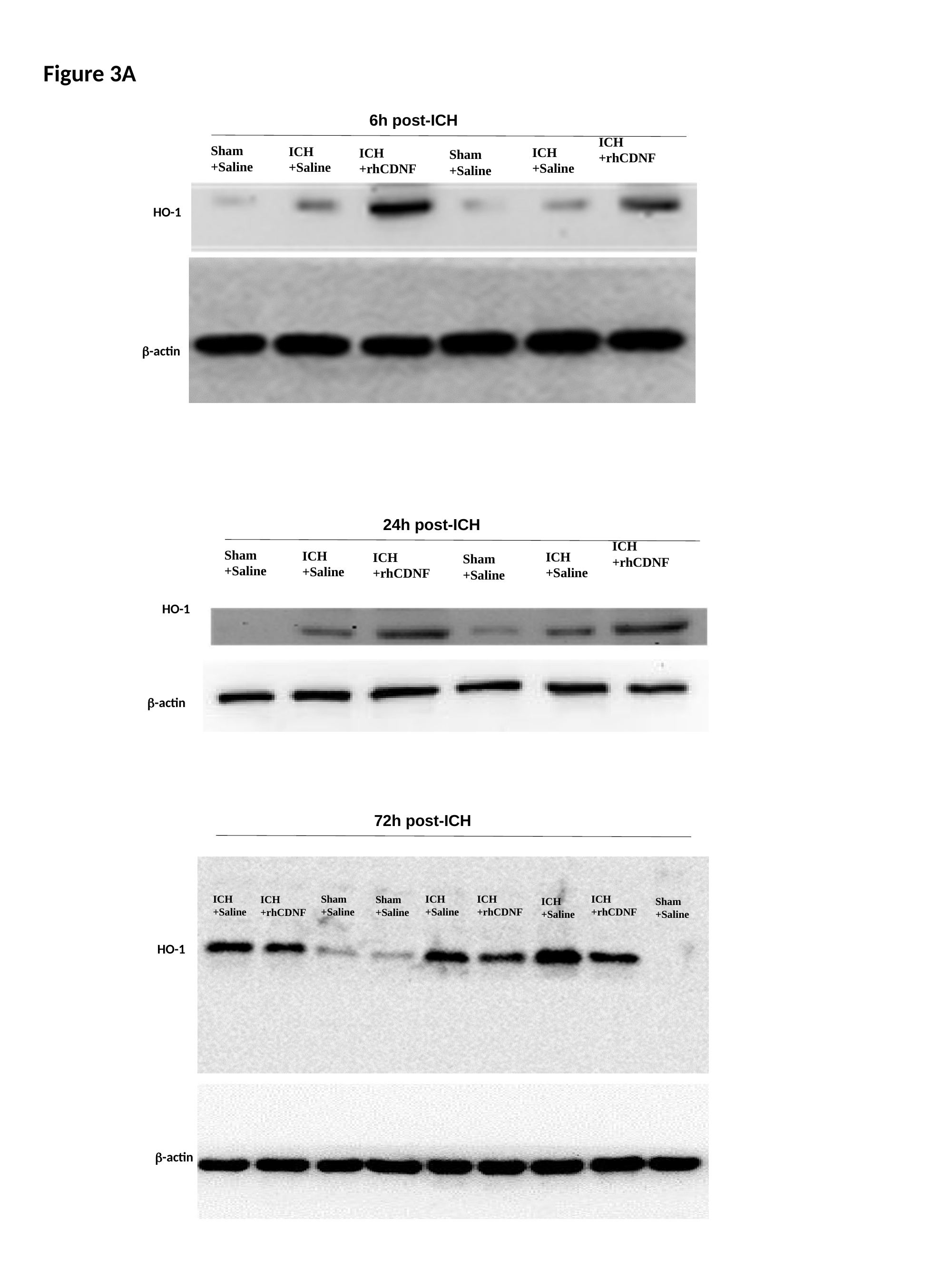

Figure 3A
6h post-ICH
ICH
+rhCDNF
Sham
+Saline
ICH
+Saline
ICH
+Saline
ICH
+rhCDNF
Sham
+Saline
HO-1
β-actin
24h post-ICH
ICH
+rhCDNF
Sham
+Saline
ICH
+Saline
ICH
+Saline
ICH
+rhCDNF
Sham
+Saline
HO-1
β-actin
72h post-ICH
ICH
+Saline
Sham
+Saline
ICH
+Saline
ICH
+rhCDNF
ICH
+rhCDNF
ICH
+rhCDNF
Sham
+Saline
ICH
+Saline
Sham
+Saline
HO-1
β-actin

## Slide 6
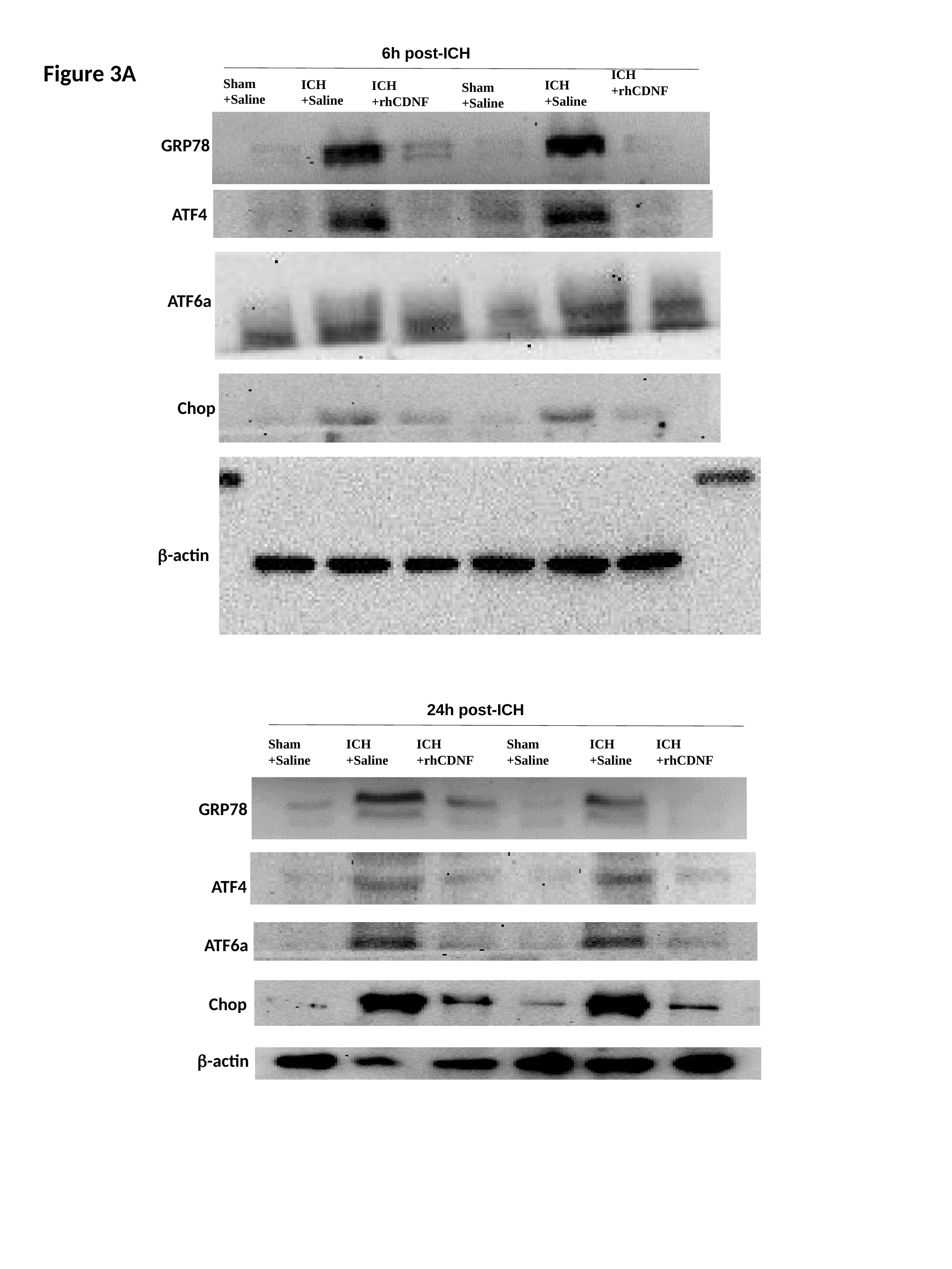

6h post-ICH
ICH
+rhCDNF
Sham
+Saline
ICH
+Saline
ICH
+Saline
ICH
+rhCDNF
Sham
+Saline
Figure 3A
GRP78
ATF4
ATF6a
Chop
β-actin
24h post-ICH
Sham
+Saline
ICH
+Saline
ICH
+rhCDNF
Sham
+Saline
ICH
+Saline
ICH
+rhCDNF
GRP78
ATF4
ATF6a
Chop
β-actin

## Slide 7
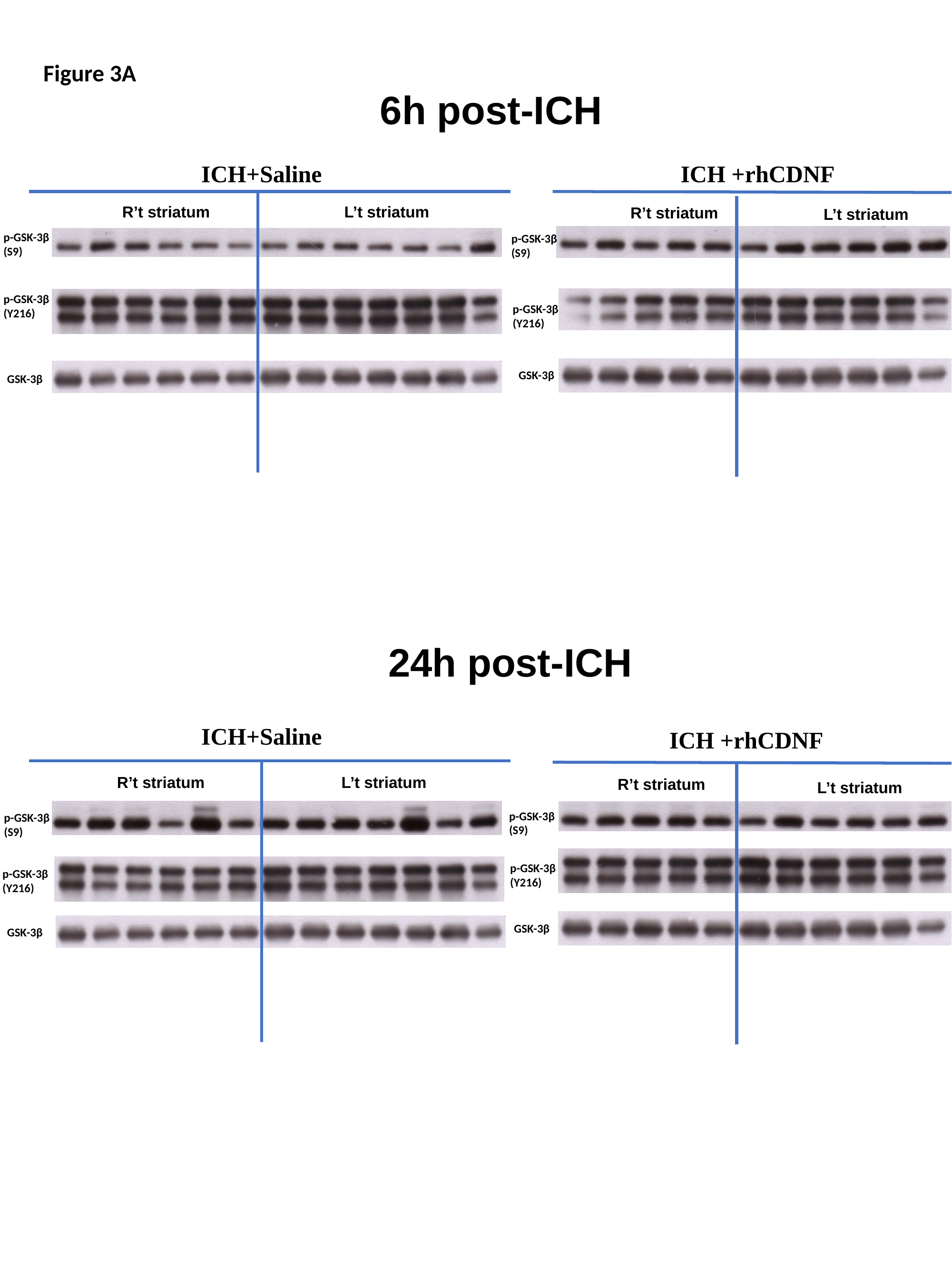

Figure 3A
6h post-ICH
ICH +rhCDNF
ICH+Saline
R’t striatum
L’t striatum
R’t striatum
L’t striatum
p-GSK-3β
(S9)
p-GSK-3β
(S9)
p-GSK-3β
(Y216)
p-GSK-3β
(Y216)
GSK-3β
GSK-3β
24h post-ICH
ICH+Saline
ICH +rhCDNF
R’t striatum
L’t striatum
R’t striatum
L’t striatum
p-GSK-3β
(S9)
p-GSK-3β
(S9)
p-GSK-3β
(Y216)
p-GSK-3β
(Y216)
GSK-3β
GSK-3β

## Slide 8
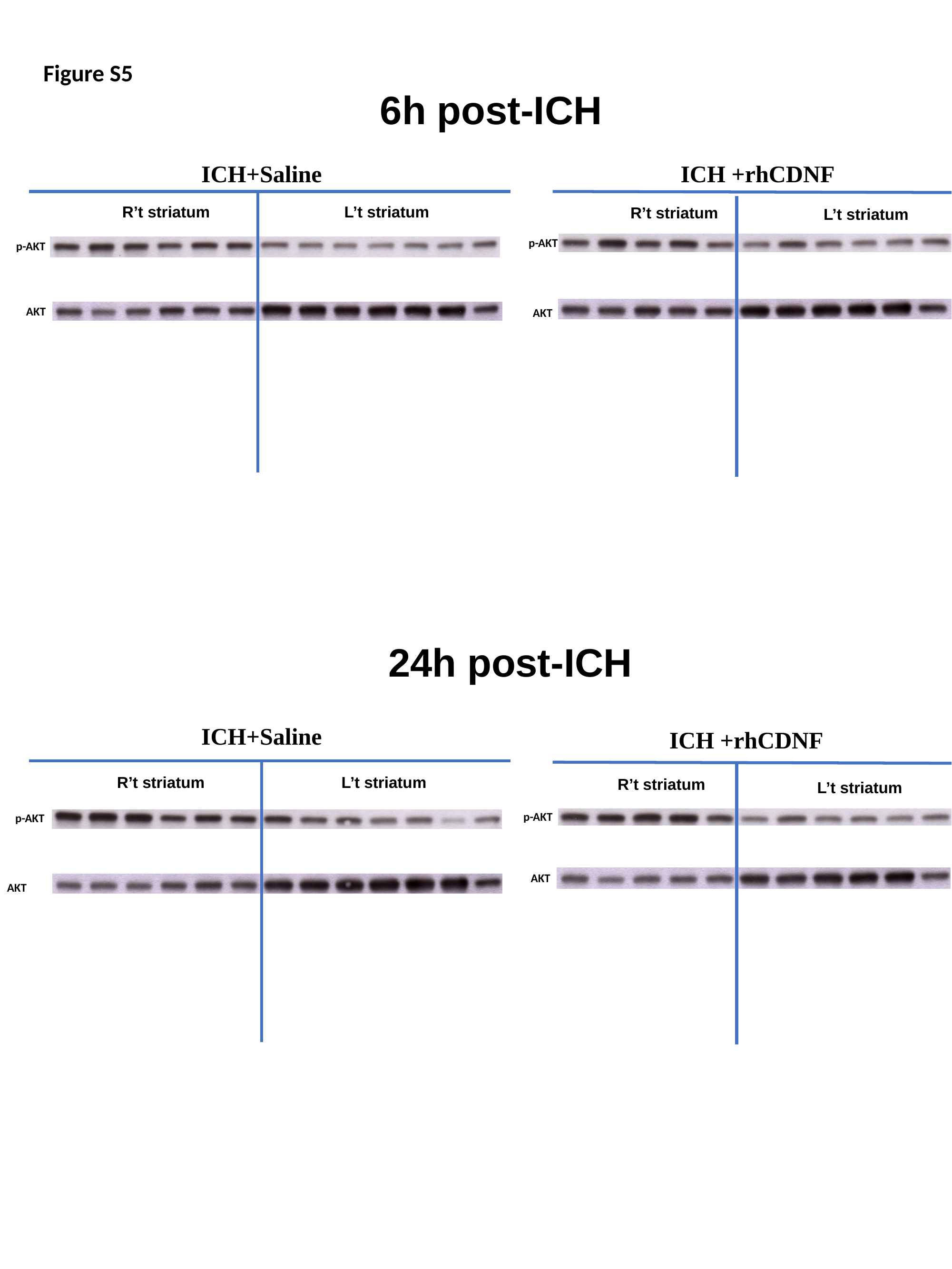

Figure S5
6h post-ICH
ICH +rhCDNF
ICH+Saline
R’t striatum
L’t striatum
R’t striatum
L’t striatum
p-AKT
p-AKT
AKT
AKT
24h post-ICH
ICH+Saline
ICH +rhCDNF
R’t striatum
L’t striatum
R’t striatum
L’t striatum
p-AKT
p-AKT
AKT
AKT

## Slide 9
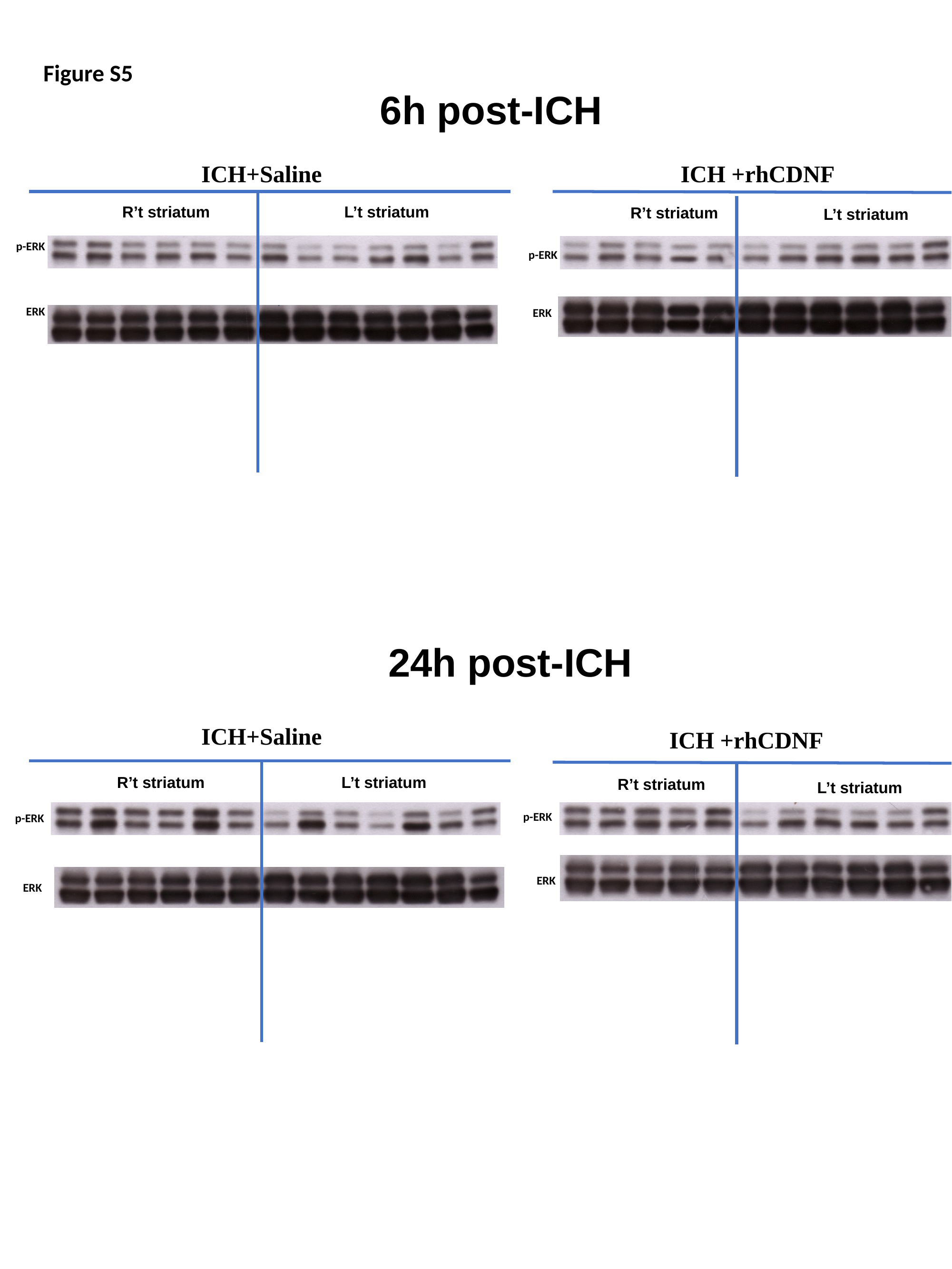

Figure S5
6h post-ICH
ICH +rhCDNF
ICH+Saline
R’t striatum
L’t striatum
R’t striatum
L’t striatum
p-ERK
p-ERK
ERK
ERK
24h post-ICH
ICH+Saline
ICH +rhCDNF
R’t striatum
L’t striatum
R’t striatum
L’t striatum
p-ERK
p-ERK
ERK
ERK

## Slide 10
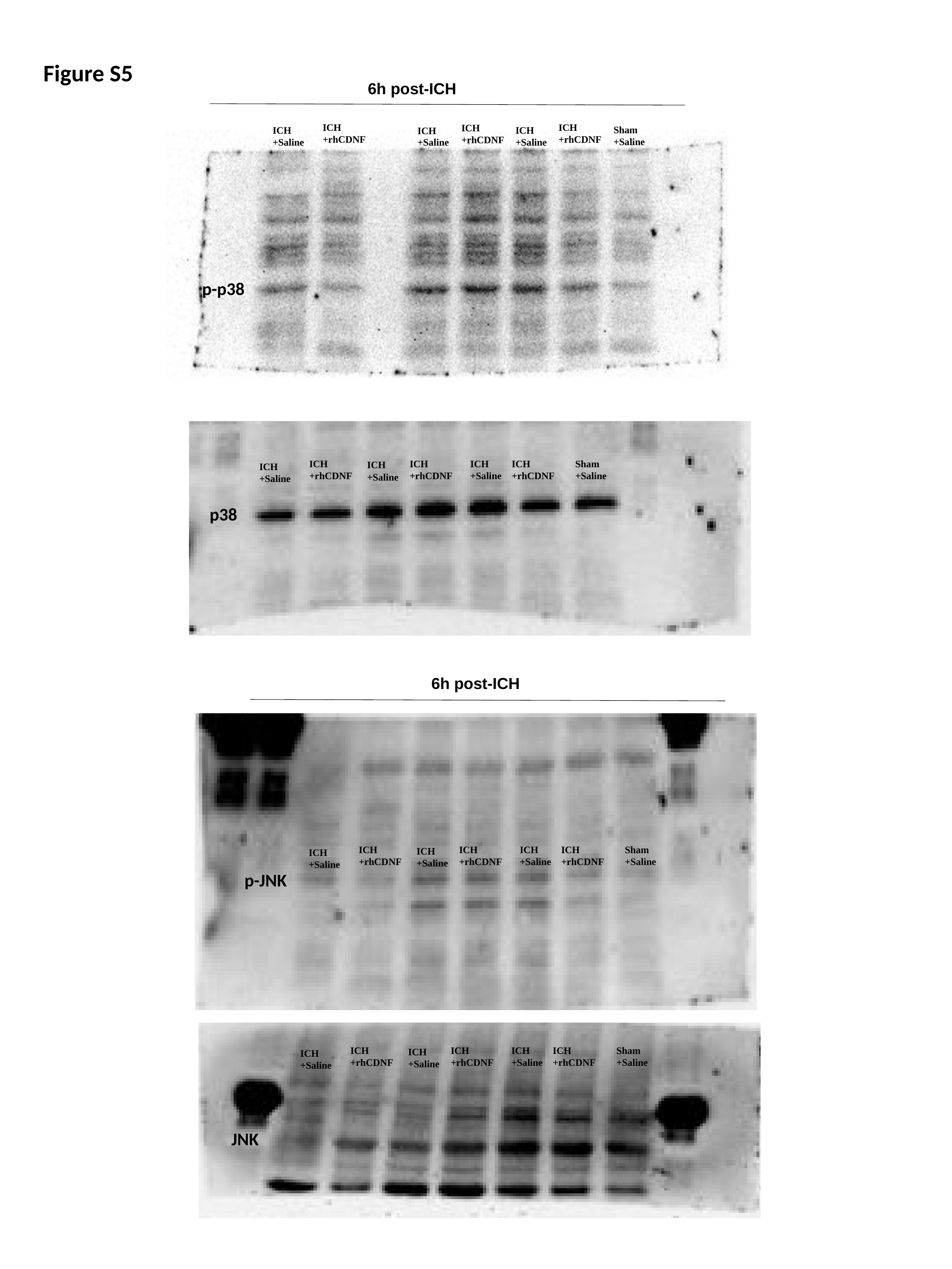

Figure S5
6h post-ICH
ICH
+rhCDNF
ICH
+rhCDNF
ICH
+rhCDNF
Sham
+Saline
ICH
+Saline
ICH
+Saline
ICH
+Saline
p-p38
ICH
+rhCDNF
ICH
+rhCDNF
ICH
+rhCDNF
Sham
+Saline
ICH
+Saline
ICH
+Saline
ICH
+Saline
p38
6h post-ICH
ICH
+rhCDNF
ICH
+rhCDNF
ICH
+rhCDNF
Sham
+Saline
ICH
+Saline
ICH
+Saline
ICH
+Saline
p-JNK
ICH
+rhCDNF
ICH
+rhCDNF
ICH
+rhCDNF
Sham
+Saline
ICH
+Saline
ICH
+Saline
ICH
+Saline
JNK

## Slide 11
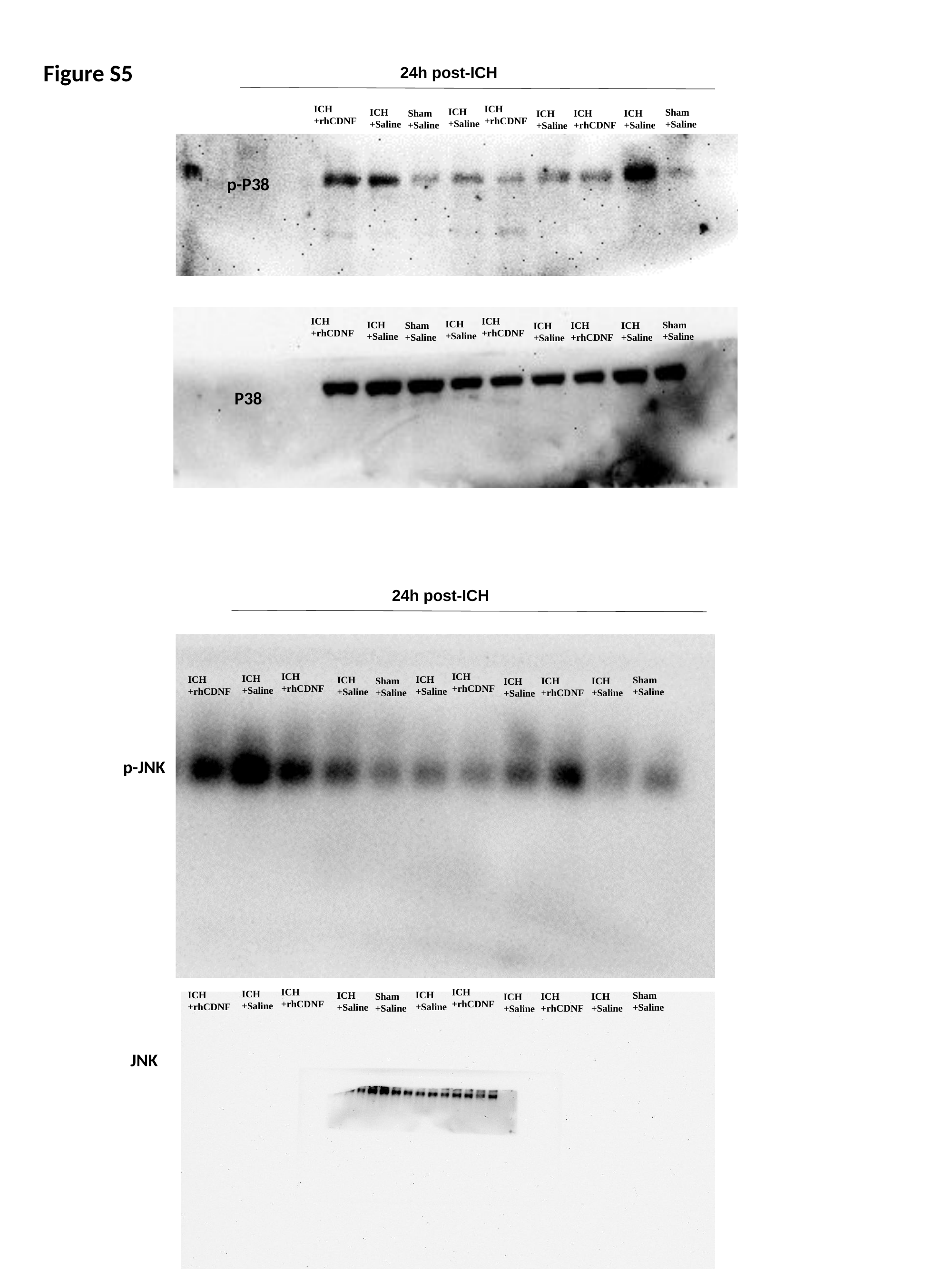

Figure S5
24h post-ICH
ICH
+rhCDNF
ICH
+rhCDNF
ICH
+Saline
ICH
+Saline
Sham
+Saline
ICH
+rhCDNF
ICH
+Saline
Sham
+Saline
ICH
+Saline
p-P38
ICH
+rhCDNF
ICH
+rhCDNF
ICH
+Saline
ICH
+Saline
Sham
+Saline
ICH
+rhCDNF
ICH
+Saline
Sham
+Saline
ICH
+Saline
P38
24h post-ICH
ICH
+rhCDNF
ICH
+rhCDNF
ICH
+Saline
ICH
+Saline
Sham
+Saline
ICH
+rhCDNF
ICH
+Saline
Sham
+Saline
ICH
+Saline
ICH
+Saline
ICH
+rhCDNF
p-JNK
ICH
+rhCDNF
ICH
+rhCDNF
ICH
+Saline
ICH
+Saline
Sham
+Saline
ICH
+rhCDNF
ICH
+Saline
Sham
+Saline
ICH
+Saline
ICH
+Saline
ICH
+rhCDNF
JNK

## Slide 12
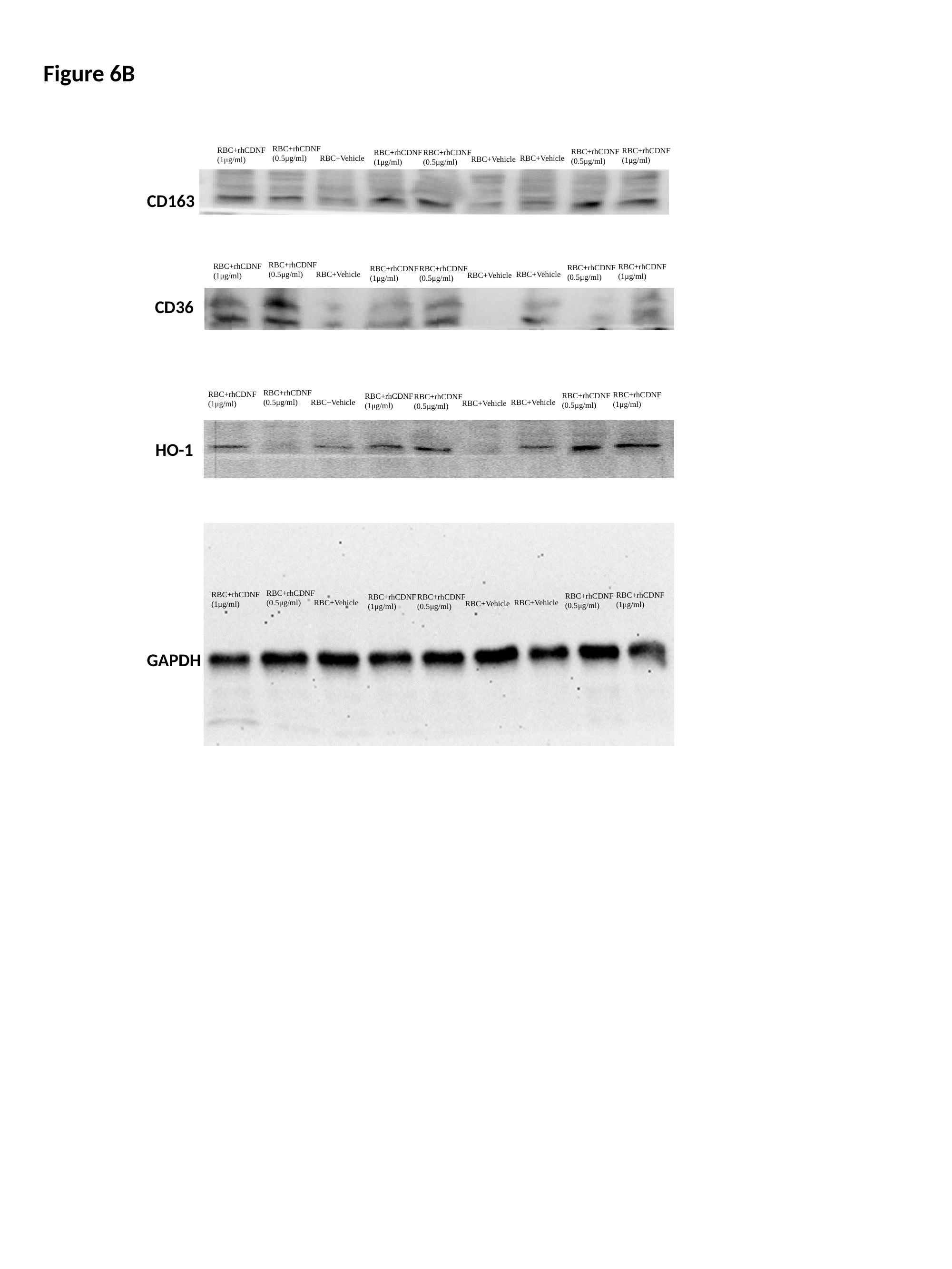

Figure 6B
RBC+rhCDNF
(0.5μg/ml)
RBC+rhCDNF
(1μg/ml)
RBC+rhCDNF
(1μg/ml)
RBC+rhCDNF
(0.5μg/ml)
RBC+rhCDNF
(1μg/ml)
RBC+rhCDNF
(0.5μg/ml)
RBC+Vehicle
RBC+Vehicle
RBC+Vehicle
CD163
RBC+rhCDNF
(0.5μg/ml)
RBC+rhCDNF
(1μg/ml)
RBC+rhCDNF
(1μg/ml)
RBC+rhCDNF
(0.5μg/ml)
RBC+rhCDNF
(1μg/ml)
RBC+rhCDNF
(0.5μg/ml)
RBC+Vehicle
RBC+Vehicle
RBC+Vehicle
CD36
RBC+rhCDNF
(0.5μg/ml)
RBC+rhCDNF
(1μg/ml)
RBC+rhCDNF
(1μg/ml)
RBC+rhCDNF
(0.5μg/ml)
RBC+rhCDNF
(1μg/ml)
RBC+rhCDNF
(0.5μg/ml)
RBC+Vehicle
RBC+Vehicle
RBC+Vehicle
HO-1
RBC+rhCDNF
(0.5μg/ml)
RBC+rhCDNF
(1μg/ml)
RBC+rhCDNF
(1μg/ml)
RBC+rhCDNF
(0.5μg/ml)
RBC+rhCDNF
(1μg/ml)
RBC+rhCDNF
(0.5μg/ml)
RBC+Vehicle
RBC+Vehicle
RBC+Vehicle
GAPDH

## Slide 13
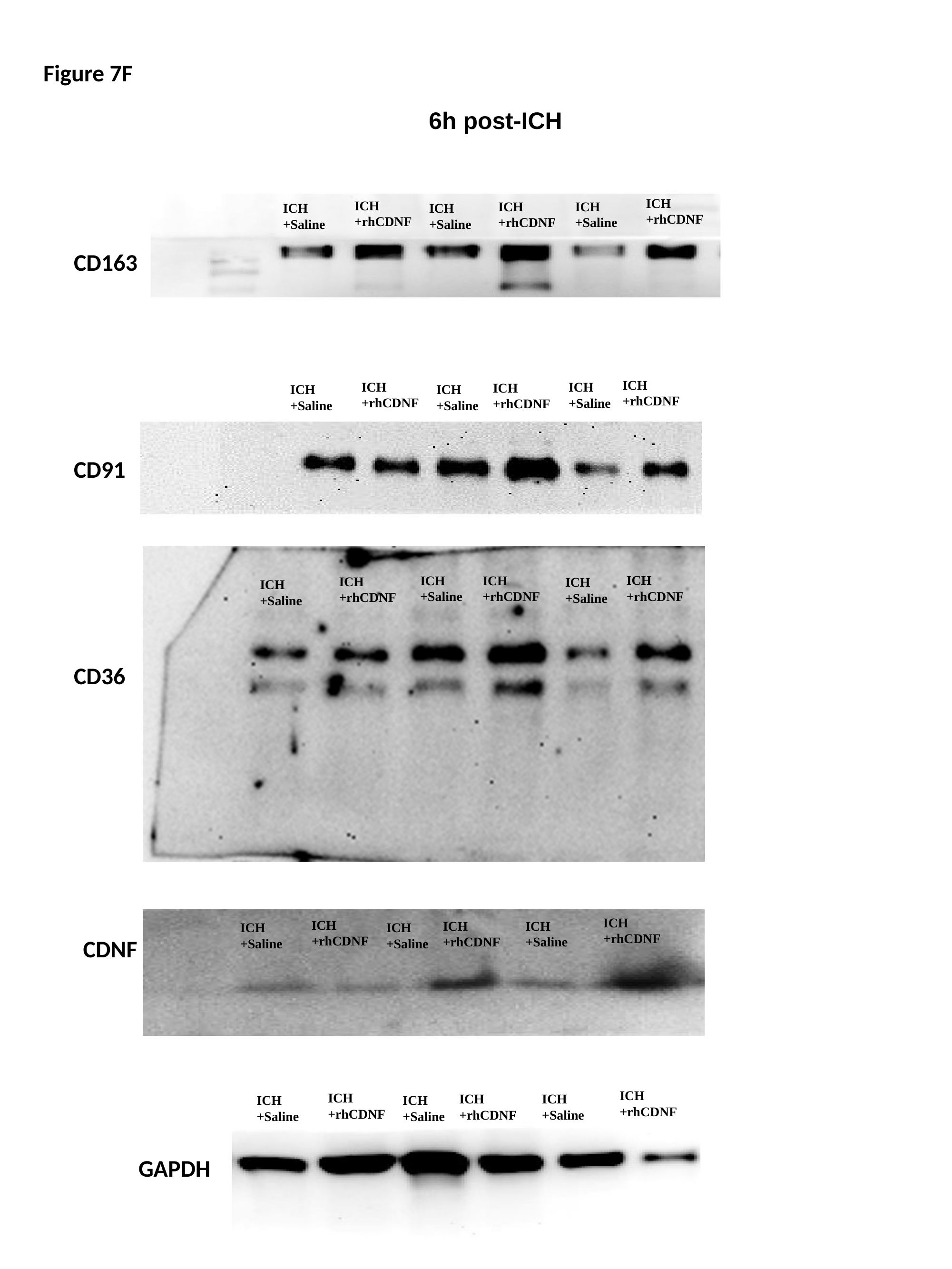

Figure 7F
6h post-ICH
ICH
+rhCDNF
ICH
+rhCDNF
ICH
+rhCDNF
ICH
+Saline
ICH
+Saline
ICH
+Saline
CD163
ICH
+rhCDNF
ICH
+rhCDNF
ICH
+Saline
ICH
+rhCDNF
ICH
+Saline
ICH
+Saline
CD91
ICH
+rhCDNF
ICH
+rhCDNF
ICH
+Saline
ICH
+rhCDNF
ICH
+Saline
ICH
+Saline
CD36
ICH
+rhCDNF
ICH
+rhCDNF
ICH
+rhCDNF
ICH
+Saline
ICH
+Saline
ICH
+Saline
CDNF
ICH
+rhCDNF
ICH
+rhCDNF
ICH
+rhCDNF
ICH
+Saline
ICH
+Saline
ICH
+Saline
GAPDH
